# Redox Active N-Heterocyclic Carbenes in Oxidative NHC Catalysis

**DOI:** 10.1021/acs.orglett.4c00731

**Published:** 2024-03-29

**Authors:** Sara Bacaicoa, Simon Stenkvist, Henrik Sundén

**Affiliations:** University of Gothenburg, Medicinaregatan 19, 413 90 Gothenburg, Sweden

## Abstract

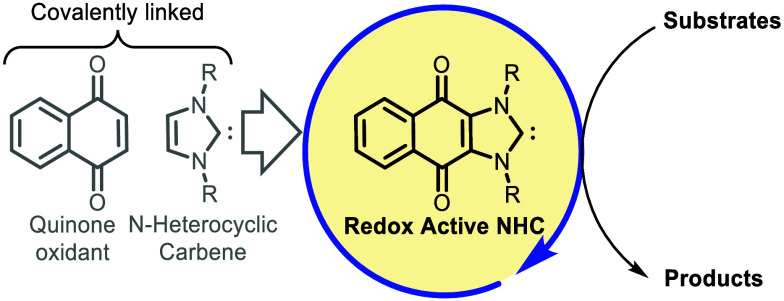

An N-heterocyclic carbene (NHC) covalently linked to a quinone introduces a novel avenue for internal oxidations within oxidative NHC catalysis. The deployment of this hybrid NHC class promotes intramolecular electronic flow in the oxidation of the Breslow intermediate to acyl azolium. The use of the redox active NHC as a catalyst is facilitated by employing aerobic regeneration, yielding carboxylic esters with efficiencies of ≤99%, while generating water as the sole byproduct.

In recent years, the field of oxidative N-heterocyclic carbene catalysis has garnered a considerable amount of attention, driven by its remarkable ability to convert simple starting materials into highly sophisticated and functionalized products.^1−5^ A notable focal point within oxidative NHC catalysis is the conversion of aldehydes into the acyl azolium intermediate, which can lead to a diverse set of products.^6−9^ The traditional approach for accessing the acyl azolium is performing an external oxidation of the Breslow intermediate utilizing a stoichiometric amount of a high-molecular weight oxidant (Scheme 1a). In our attempt to optimize these reactions, we speculated that enhancing the efficiency of electron transfer in the oxidation process might be achieved by covalently linking the oxidant to the Breslow intermediate. This strategic refinement is envisioned through the utilization of a catalyst that combines NHC and redox functionalities. Covalent attachment of this hybrid catalyst to the substrate in the Breslow intermediate introduces the possibility of an internal electron transfer event originating from the quinone moiety of the catalyst to the substrate, resulting in the formation of the acyl azolium (Scheme 1b).

**Scheme 1:**
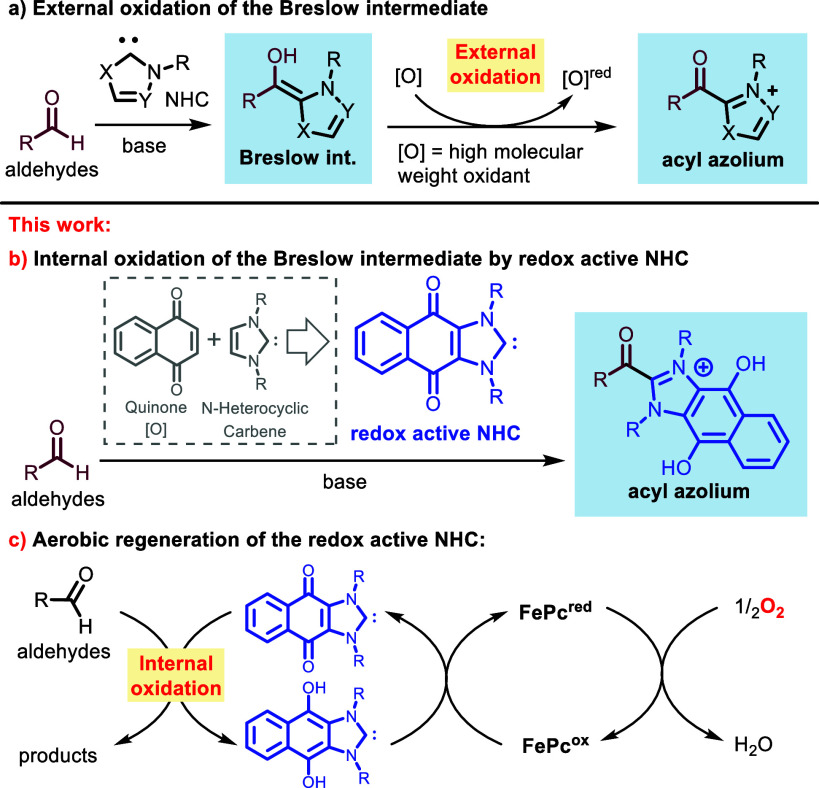
Internal Oxidation by a Hybrid NHC Catalyst This work reports a new strategy for accessing the acyl azolium intermediate by utilizing a redox active NHC catalyst.

Here we demonstrate that a redox active NHC **4a**,^10^ originally synthesized by Bielawski and co-workers,^11^ can exhibit proficiency in various chemical transformations that typically necessitate the addition of a stoichiometric high-molecular weight external oxidant. Furthermore, we demonstrate the efficient *in situ* reoxidation of the redox active NHC at room temperature, leveraging atmospheric oxygen as the terminal oxidant^12−14^ in the presence of an iron-based co-catalyst (Scheme 1c).

Our study commenced by investigating the potential of **4a** serving both as an NHC catalyst and as an oxidant in the oxidative esterification of aldehydes. As it turns out, when **4a** was employed in a stoichiometric amount it was able to transform *trans*-cinnamaldehyde (**1a**) into methyl cinnamate (**3aa**) in 90% yield (Table 1, entry 1) (see the Supporting Information for optimization).

**Table 1:**
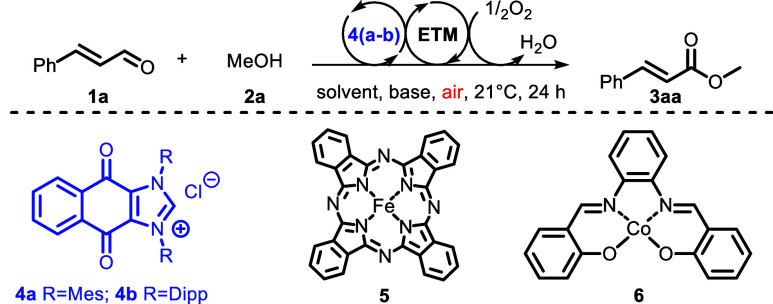
Optimization of the Reaction Conditions^a^

entry	pre-NHC (mol %)	ETM (mol %)	solvent	base (mol %)	yield of **3aa**^b^ (%)
1^c^	**4a** (110)	–	THF	DBU (120)	90
2	**4a** (20)	–	EtOAc	K_2_CO_3_ (50)	33
3	**4a** (20)	**5** (3)	MeCN	DBU (25)	82
4	**4a** (20)	**5** (3)	2-Me-THF	DBU (25)	74
5	**4a** (20)	**5** (3)	CHCl_3_	DBU (25)	62
6	**4a** (20)	**5** (3)	DCM	DBU (25)	69
7	**4a** (20)	**5** (3)	MeOH	DBU (25)	91^d^
8	**4a** (20)	**5** (3)	toluene	DBU (25)	64
9	**4a** (20)	**5** (3)	EtOAc	DBU (25)	75
10	**4a** (20)	**5** (3)	EtOAc	K_2_CO_3_ (25)	83
11	**4a** (20)	**5** (3)	EtOAc	K_2_CO_3_ (50)	90^e^
12	**4a** (20)	**6** (3)	MeCN	DBU (25)	90
13^f^	**4a** (20)	**5** (3)	EtOAc	K_2_CO_3_ (50)	25
14	**4a** (15)	**5** (3)	EtOAc	K_2_CO_3_ (50)	76
15	**4b** (20)	**5** (3)	EtOAc	K_2_CO_3_ (50)	78

a General conditions: 0.25 mmol of **1a**, 500 μL of solvent, 24 h, 21 °C. ETM is an electron transfer mediator.

b GC-FID yield.

c N_2_ atmosphere, 5 h, 0.1 mmol of **1a**, 300 μL of dry THF, 21 °C.

d Reaction time of 6 h.

e Isolated yield.

f With 4 equiv of methanol.

The effectiveness of our internal oxidation was compared to that of the equivalent external oxidation by separately employing 1,3-bis(mesityl)imidazolium chloride as NHC precursor **8** and 1,4-naphthoquinone as oxidant **7** (Figure 1). The conversion to product **3aa** was analyzed by GC-FID using dodecane as the internal standard and sampling aliquots every 30 min from both reactions.

**Figure 1:**
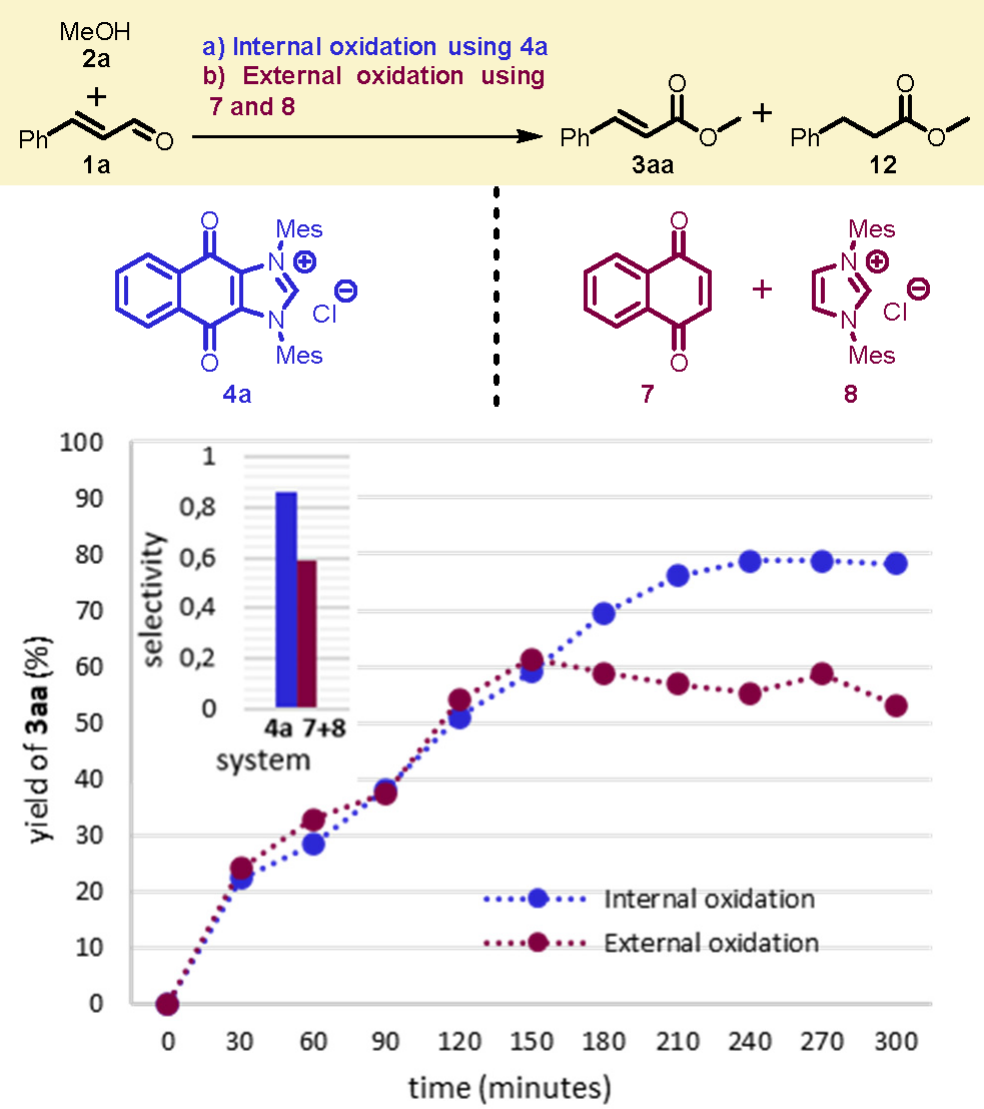
Selectivity of internal oxidation vs external oxidation. Comparison of reaction profiles and selectivity between internal oxidation using redox active NHC **4a** and external oxidation combining **7** and **8**. Selectivity (300 min) = (yield of **3aa**/**1a** consumed) × 100.

As it turns out, the performance of internal oxidation with redox active **4a** is better than that of external oxidation using a combination of an NHC precatalyst (**8**) and an oxidant (**7**). Judging by the kinetic profile (Figure 1), external oxidation stops after 150 min at 60% conversion due to the formation of side products This was confirmed by NMR analysis of the crude reaction mixture in which saturated ester **12** could be identified as the major side product. Conversely, saturated ester **12** could not be found in the NMR analysis of the crude reaction mixture corresponding to the internal oxidation using **4a**. NHC-catalyzed formation of saturated esters from α,β-unsaturated aldehydes is known and can be used as an indication of inefficient oxidation of the homoenolate intermediate.^9^ In light of these data, we can conclude that we have a more selective reaction and a more efficient oxidation using redox active hybrid **4a** than in the reaction using the equivalent separated system with **7** and **8**.

Having verified that a stoichiometric amount of **4a** can perform the oxidative esterification of aldehydes, and to avoid the reduced form of **4a** as a stoichiometric byproduct, we were prompted to investigate whether **4a** could be used in substoichiometric amounts in combination with oxygen as the terminal oxidant. To achieve this, we performed the reaction under an open atmosphere aiming to directly reoxidize **4a** with oxygen, finding that this process is inefficient, producing **3aa** in only 33% yield in 24 h (Table 1, entry 2). In previous studies, we^15−21^ and others^22−24^ have demonstrated that the addition of a co-catalyst is beneficial to the outcome of the reaction. Therefore, **4a** was combined with 3 mol % iron(II) phthalocyanine (FePc, **5**) or Co(II) salophen (**6**).

Optimization of the aerobic oxidative esterification of aldehydes revealed that redox active NHC **4a** catalyzed the esterification of **1a** and generally works well using oxygen as the terminal oxidant. Our optimization commenced by investigating the role of the reaction solvent (Table 1, entries 3–9; see the Supporting Information for complete optimization data). The reaction performs well in polar aprotic solvents such as 2-methyl tetrahydrofuran, chloroform, dichloromethane, and ethyl acetate (Table 1, entries 3–6 and 9, respectively), where acetonitrile and ethyl acetate gave the best yields (82% and 75%, respectively). The nonpolar solvent toluene was also compatible with aerobic oxidation; however, the yield of product **3aa** is lower [64% (Table 1, entry 8)]. When the acylation was performed neat in methanol, the reaction time could be reduced to 6 h, affording a 91% yield of **3aa** (Table 1, entry 7), which is most likely affected by the higher concentration of the nucleophile. The influence of the base was also investigated (for further optimization data, see the Supporting Information), revealing that the reaction performed well with 0.25 equiv of potassium carbonate with the yield being optimal upon addition of 0.5 equiv of the same base (Table 1, entries 10 and 11).

The Co(II) salophen complex (**6**) was also tested as a redox active catalyst for this aerobic reaction, but despite the good result, we were inclined to continue by using **5** because it is a greener alternative (Table 1, entry 12). Furthermore, reducing the amount of methanol to 4 equiv was detrimental to the reaction performance, providing **3aa** in 25% yield (Table 1, entry 13). To optimize the amount of **4a**, the reaction conditions were challenged by using 15 mol % **4a**, resulting in a decreased yield (Table 1, entry 14). Finally, a more sterically hindered redox active NHC **4b** was tested (Table 1, entry 15), but the yield was lower than that using **4a** under the same conditions (Table 1, entry 11).

With our optimized reaction conditions in hand, we tested the compatibility of our method with a variety of hydroxyl nucleophiles and aldehydes (Table 2). Halogens on the aromatic ring of the α,β-unsaturated aldehyde were appropriate candidates for this reaction. For example, *p*-Cl, *p*-F, and *o-*Br gave the corresponding halogenated methyl esters in 87%, 84%, and 83% yields, respectively (**3ba–3da**, respectively). Electron-rich α,β-unsaturated aldehydes with a methoxy at the *para* position of the aromatic ring were well tolerated, affording methyl ester **3ea** in 62% yield and amiloxate (**3eb**) in 51% yield. The presence of a nitro group at the *ortho* position of the aromatic ring of the α,β-unsaturated aldehyde positively affected the reaction, providing **3fa** in 98% yield. In addition, different hydroxyl group containing molecules were reacted with the α,β-unsaturated cinnamaldehyde to assess the compatibility of the method with a diverse set of nucleophiles. Phenols and methoxyphenols turned out to be excellent nucleophiles for this method, providing the corresponding phenyl esters in 94% and 66% yields (**3ac** and **3ad**, respectively). Glycerol 1,2-carbonate (**2e**) was also a suitable nucleophile for the reaction, affording ester **3ae** in 78% yield. Furthermore, the bicyclic natural product myrtenol (**2f**) could be reacted with **1a** to afford 74% of the corresponding ester **3af**. Further investigating the substrate scope on the aldehyde moiety, we found the heterocyclic nicotinic aldehyde could be reacted with **2a** to afford product **3ga** in 79% yield. Extended aromatics on the aldehyde moiety are also compatible with the reaction, finding an example in product **3ha** that was isolated in 63% yield. Reaction with halogen-substituted benzaldehydes gave the corresponding ester congeners **3ia–3ka** in high to excellent yields (≤98%). Moreover, disubstituted benzaldehyde **1l** was compatible with the method, affording product **3la** in 85% yield. Electron-withdrawing groups on the benzaldehyde are well tolerated by the reaction, and the highest yield was achieved with cyano *para*-substituted benzaldehyde, resulting in a yield of 99% (**3ma**).

**Table 2:**
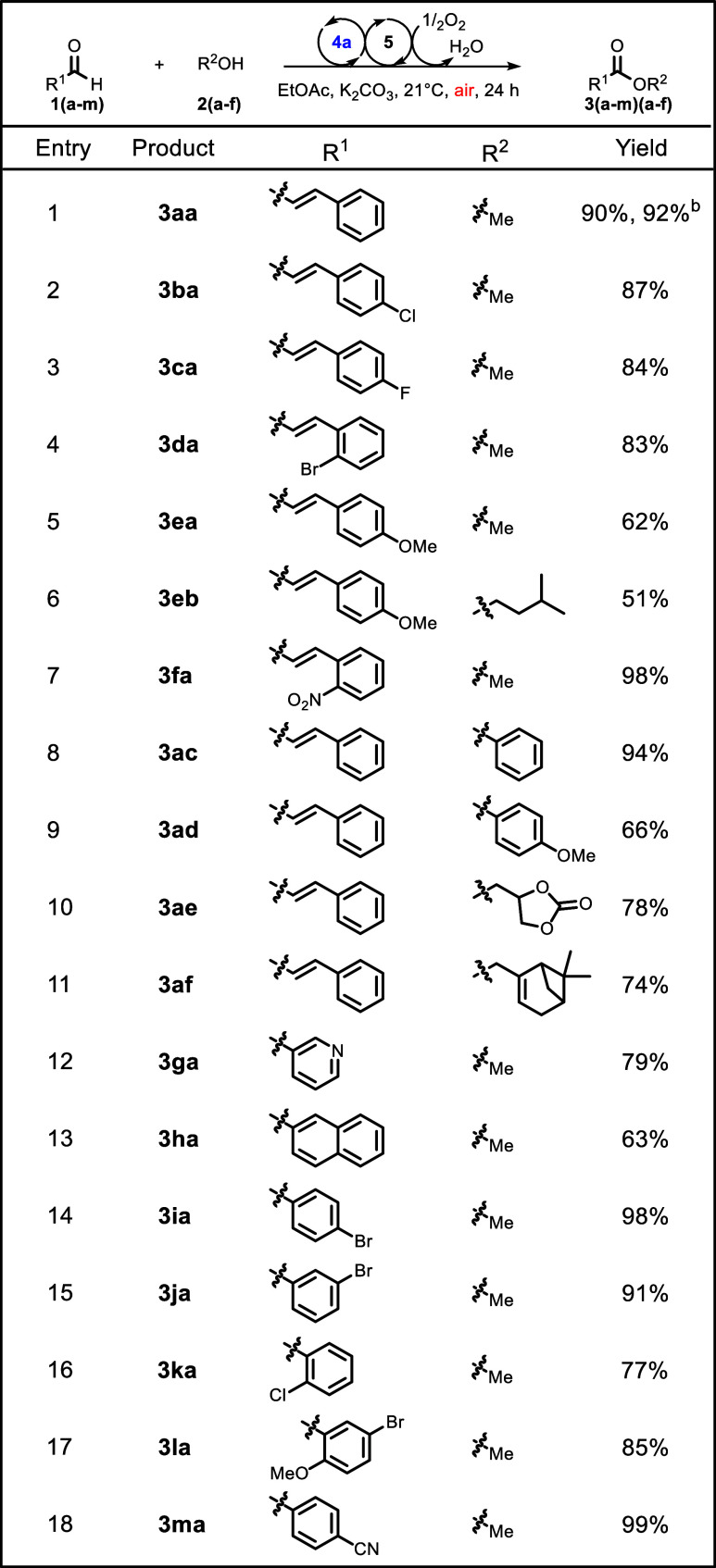
Substrate Scope of the Aerobic Redox Active N-Heterocyclic Carbene-Catalyzed Esterification^a^

a Substrate scope determined under the optimal conditions from Table 1.

b Isolated yield from a 1 mmol scale experiment.

It is also possible to utilize hybrid catalyst **4a** for the oxidative amidation of aldehydes. For example, the synthesis of amide **9** was accomplished by employing a stoichiometric amount of **4a** in combination with **1a** and pyrrolidine (Scheme 2).^25^ It was also possible to use 2-oxazolidinone as a nucleophile, rendering compound **11** in 54% yield.

**Scheme 2:**
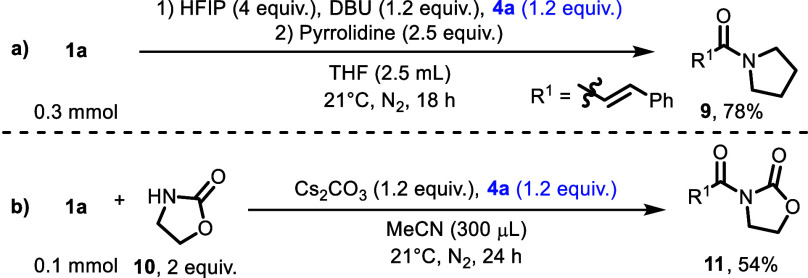
Amide Synthesis with Stoichiometric Redox Active N-Heterocyclic Carbene HFIP = hexafluoroisopropanol. DBU = 1,8-diazabicyclo[5.4.0]undec-7-ene. N_2_ = nitrogen atmosphere.

Additionally, our aerobic method can also be applied in the synthesis of polymer precursors such as diesters of type **3na** or oligomers like **3ng** (Scheme 3). Both products **3na** and **3ng** are interesting as they can be used in the synthesis of polyethylene terephthalate.^24,26^ For other reactions that we have tested with **4a** as a catalyst, see the Supporting Information.

**Scheme 3:**
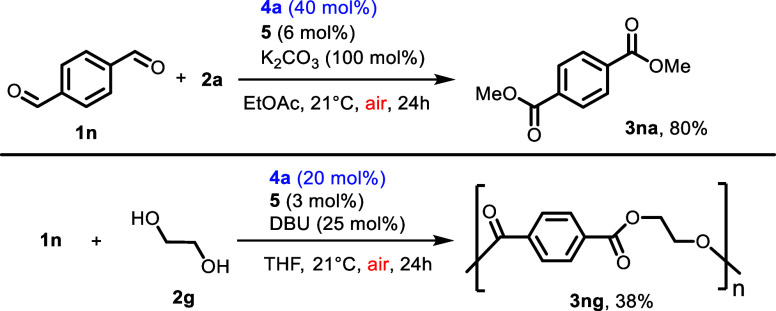
Aerobic Synthesis of the Terephthalate Oligomer Oligomer synthesis by aerobic redox active NHC-catalyzed acylation. See the Supporting Information for the experimental synthetic procedures. DBU = 1,8-diazabicyclo[5.4.0]undec-7-ene.

We propose a mechanism for the internal oxidative esterification of aldehydes (Scheme 4) in which redox active NHC **4a**^**ox**^ is created *in situ* by deprotonation of **4a**. Then, Breslow intermediate **I** is formed after a nucleophilic attack by NHC **4a**^**ox**^ on the acyl position of an aldehyde. Successively, the electrons rearrange, reducing the quinone moiety of the NHC by subtracting electrons from the enamine of the NHC–aldehyde adduct as indicated in **I** and **II**, leading to an acyl azolium **III**. Upon nucleophilic attack by a nucleophile on acyl azolium **III**, the product is formed, generating carbene **4a**^**red**^, which is later reoxidized to **4a**^**ox**^ by oxygen with FePc as an intermediate in electron transfer.

**Scheme 4:**
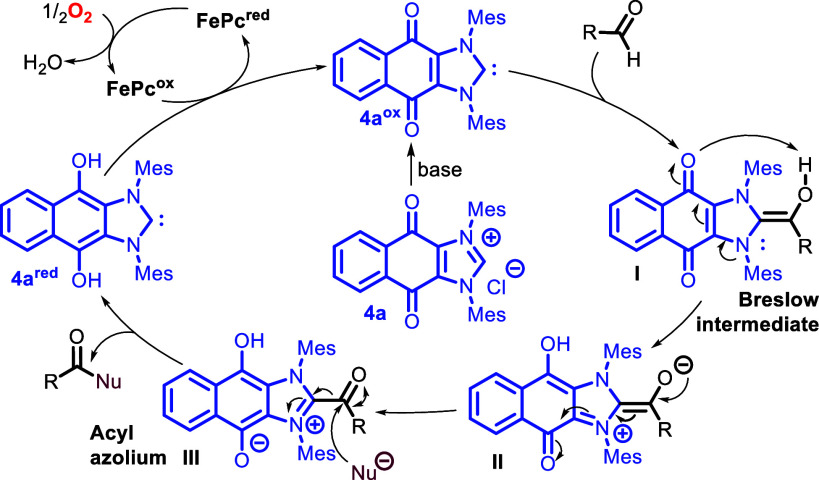
Proposed Catalytic Cycle

In summary, a novel internal oxidation strategy has been conceptualized by a hybrid redox active NHC within oxidative NHC catalysis that can perform acylation and amide bond formation reactions. Esters and amides could be efficiently synthesized via aerobic regeneration of **4a** in a single step, in yields of ≤99% for esters and ≤78% for amides. We have conceptualized the possibility of internal oxidations in oxidative NHC catalysis by employing a hybrid redox active NHC catalyst, which offers a new perspective for the future development of efficient coupling reactions with aldehydes.

## Data Availability

The data underlying this study are available in the published article and its Supporting Information.

## Supporting Information Available

General experimental procedures, additional optimization data, characterization data, and copies of ^1^H and ^13^C NMR spectra (PDF)
