# Supplementary material for: Redox Active N-Heterocyclic Carbenes in Oxidative NHC Catalysis
Source: Org Lett. 2024 Mar 29;26(15):3114–8. doi: 10.1021/acs.orglett.4c00731 (PMC11040713; doi:10.1021/acs.orglett.4c00731)

Supporting Information for

Redox Active N-Heterocyclic Carbenes in Oxidative NHC Catalysis

Sara Bacaicoa, Simon Stenkvist, Henrik Sundén*

Chemistry and Molecular Biology, University of Gothenburg, Kemivägen 10, 41296 Gothenburg, Sweden

henrik.sunden@chem.gu.se

Table of contents

1. General information **3**

1.1 Procedure for GC-FID studies *3*

1.2. Procedure for reaction profile experiments and selectivity measurement *3*

2. Additional optimization data **4**

3. Synthetic procedures **5**

3.1. General synthetic procedure for aerobic oxidative acylation using redox active NHC 4a *5*

3.2. Redox active NHC catalyzed amide synthesis *5*

4. 1 mmol scale synthesis of 3aa **5**

5. General procedure for the synthesis of redox active N-heterocyclic carbenes **6**

6. Additional NHC catalyzed reactions **6**

6.1. Aerobic [3+3] cyclization using 4a *6*

6.2. Aerobic [2+4] cyclization using 4a *7*

7. NMR characterization of compounds **7**

8. References **12**

1. General information

Compounds **1b**, **1d-1m**, **2a-2g** and **7**, **8**, **10** were purchase from Sigma Aldrich Sweden AB, compounds **1c** and **5** were purchased from TCI Europe, and **1a** was purchased from Fisher Scientific. All solvents were purchased from VWR. All moisture-sensitive reactions were performed under nitrogen with standard Schlenk techniques. THF and toluene were retrieved from the SPS brand inert, Model number PS-MD-5/7 for anhydrous reactions. ^{1}H NMR (400, 600 MHz), ^{13}C NMR (101,151 MHz) and ^{19}F NMR (376 MHz) spectra were recorded on 400 MHz Bruker, 600 MHz Bruker Avance NEO, 5 mm QCI-P cryoprobe. Chemical shifts (δ) are reported referenced to the residual solvent peak of $CDCl_3$ (^{1}H = 7.26 ppm, singlet; ^{13}C = 77.16 ppm, triplet) or DMSO (^{1}H = 2.50 ppm, singlet; ^{13}C = 40.45 ppm, septet). Reaction progress was monitored by thin layer chromatography (TLC) with Merck TLC plates precoated with silica gel 60 F254 (0.25 mm thickness) and was visualized with UV-light (254 nm). Automated column chromatography was performed on Biotage Isolera^{TM} Spektra One. GC-FID spectra were recorded on Agilent Technologies 7820A GC System. For collecting HRMS data the equipment used was Agilent QTOF 6520 equipped with an electrospray interface and TOF mass analyzer operated in positive ionization mode.

1.1 Procedure for GC-FID studies

The yield of the optimization studies and kinetic studies was measured by GC-FID, using dimethyl sulfone as internal standard. Aliquots of 20 µL were taken from the reaction, diluted with ethyl acetate (1.5 mL) and analyzed using GC-FID from Agilent Technologies. GC-FID spectra were recorded on Agilent Technologies 7820A GC System and nitrogen was used as carrier gas. GC-FID method used: 50 °C for 0.2 minutes, 50 °C/min to 220 °C, 10 °C/min to 250, 40 °C/min to 300 °C. Ret. time (dimethylsulfone) = 2.65 min.

The response factors (F) were calculated using the following relationship:

$$\frac{A_x}{[X]} = F\frac{A_S}{[S]}$$

Ax = Area of the peak corresponding to the analyte.

[X] = Concentration of the analyte.

F = Response factor

As = Area of the peak corresponding to the internal standard

[S] = Concentration of the internal standard

1.2. Procedure for reaction profile experiments and selectivity measurement

The quantification of **3aa** and **1a** during the development of the reactions (Figure 1, a and b) were measured by GC-FID, using dodecane as internal standard. Aliquots of 20 µL were taken from the reactions each 30 minutes, diluted with ethyl acetate (1.5 mL) and analyzed using GC-FID from Agilent Technologies. GC-FID spectra were recorded on Agilent Technologies 7820A GC System and nitrogen was used as carrier gas. GC-FID method used: 50 °C for 0.2 minutes, 50 °C/min to 220 °C, 10 °C/min to 250, 40 °C/min to 300 °C. Ret. time (dodecane) = 3.66 min.

As general experimental conditions, **1a** (0.3 mmol, 1 equiv.), **2a** (97.3 mL, 8 equiv.), Cs_2CO_3 (117.4 mg, 1.2 equiv.), dry and degassed tetrahydrofuran (2ml) where mixed, with inert atmosphere at a temperature of 21°C. For the internal oxidation reaction profile (Figure 1, a), **4a** (169.7 mg, 1.2 equiv.) was added to the reaction**.** For the external oxidation profile (Figure 1, b), **7** (57.0 mg, 1.2 equiv.) and **8** (122.9 mg, 1.2 equiv.) were added to the reaction mixture.

2. Additional optimization data

Optimization of the acylation using stoichiometric redox active NHC 4a

*Table S1. Optimization of the acylation using redox active NHC **4a***

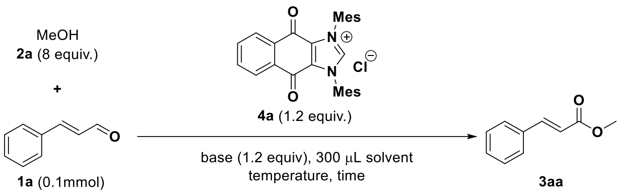

Entry	Deviation from standard conditions[a]	Yield **3aa**[b]
1	No change	90%
2	Open atmosphere	73%
3	Acetonitrile as solvent	64%[c]
4	Dichloromethane as solvent	58%
5	Toluene as solvent	57%
6	Without base	n.r.
7	Triethylamine (1.2 equiv.) instead of DBU	66%
8	TBD (1.2 equiv.) instead of DBU	66%
9	Sodium acetate (1.2 equiv.) instead of DBU	80%
10	Potassium tert-butoxide (1.2 equiv.) instead of DBU	43%
11	Cs_2CO_3 (1.2 equiv.) instead of DBU, 4 hours	86%
12	Cs_2CO_3 (1.2 equiv.) instead of DBU, 40°C, 3 hours	72%
13	Cs_2CO_3 (1.2 equiv.) instead of DBU, 50°C, 2 hours	91%

[a]Standard conditions: Cinnamaldehyde **1a** (0.1 mmol), redox active NHC **4a** (1.2 equiv.), DBU (1.2 equiv.), methanol (8 equiv.), dry and degassed tetrahydrofuran (300 mL), 21°C, inert atmosphere, 6 hours. [b]GC-FID yield using dodecane as internal standard. [c]Used dimethylsulfone as internal standard. TBD: Triazabicyclodecene. DBU: 1,8-diazabicyclo(5.4.0)undec-7-ene. [a]GC-FID yield. n.r.: No reaction.

Base screening for the aerobic acylation reaction using redox active NHC 4a

Table S2. Additional optimization data; base screening.

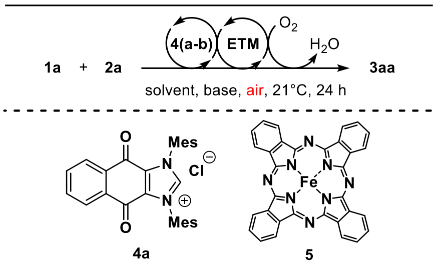

Entry	ETM (mol%)	Base (mol%)	Yield **3aa**[a]
1	**5** (3)	DBU (50)	66%
2	**5** (3)	Et_3N (50)	74%
3	**5** (3)	TBD (50)	44%
4	**5** (3)	Na_2CO_3 (50)	55%
5	**5** (6)	K_2CO_3(50)	70%
6	**5** (8)	K_2CO_3(50)	82%

General conditions: 0.25mmol **1a**, 8 equiv. **2a**, 20 mol% **4a**, 500 μL EtOAc, 50 mol% base, 24 hours, 21°C. [a]GC-FID yield using dimethylsulfone as internal standard.

3. Synthetic procedures

3.1. General synthetic procedure for aerobic oxidative acylation using redox active NHC 4a

To a 1 ml V-vial equipped with a stir-bar, aldehyde (0.25 mmol, 1 equiv.), Iron (II) phthalocyanine (0.0075 mmol, 4.3 mg, 3 mol%), redox NHC **4a** (0.05 mmol, 23.6 mg, 20 mol%), potassium carbonate (0.1255 mmol, 17.4 mg, 0.5 equiv.), alcohol (8 equiv.) and 0.5 ml of ethyl acetate were added. The vial was equipped with a lid with two holes (2 mm in diameter). The mixture was allowed to stir at room temperature (21°C) for 24 hours. The crude product was purified with an automated column chromatography and dried under reduced pressure. All esters in the substrate scope were synthesized using this general procedure.

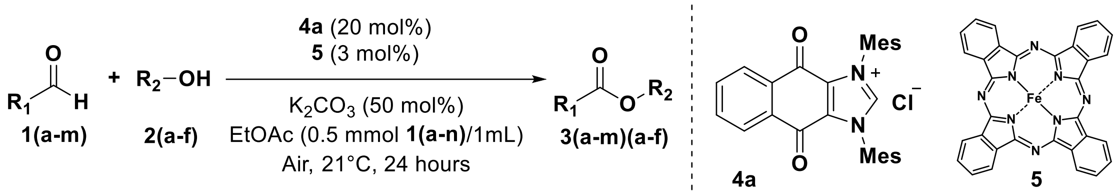

3.2. Redox active NHC catalyzed amide synthesis

3.2.1. Synthesis of pyrrolidinyl cinnamate **9**

To a 5 ml V-vial equipped with a stir-bar, cinnamaldehyde (0.30 mmol, 1 equiv.), redox NHC **4a** (0.36 mmol, 169.7 mg, 1.2 equiv.), 1,8-diazabicyclo[5.4.0]undec-7-ene (0.36 mmol, 54.9 mg, 1.2 equiv.), hexafluoro isopropanol (1.20 mmol, 218.7 mg, 4 equiv.) and 2.5 mL of dry and degassed THF were added, leaving the reaction to stir for overnight. The next day, pyrrolidine (0.75 mmol, 53.4 mg, 2.5 equiv.) was added and the mixture was allowed to stir at room temperature (21°C) for 18 hours. The crude product was evaporated under reduced pressure and redissolved in 10 mL of ethyl acetate, extracting with saturated NH_4Cl (10mL x 2). After the separation, the organic layer was dried over anhydrous sodium sulphate, evaporated under reduced pressure, and purified with an automated column chromatography (pentane/EtOAc, 2:3), affording 47 mg of compound **9**, 78% yield.

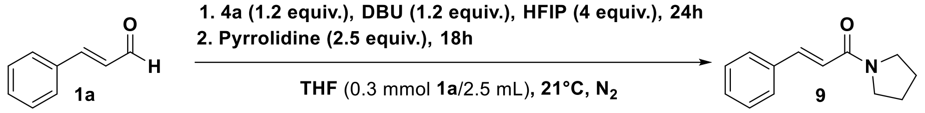

3.2.2. Synthesis of 3-cinnamoyloxazolidin-2-one **11**

To a 1 ml V-vial equipped with a stir-bar, cinnamaldehyde (0.10 mmol, 13.2 mg, 1 equiv.), redox NHC **4a** (0,12 mmol, 56.6 mg, 1.2 equiv.), caesium carbonate (0,12 mmol, 39.1 mg, 1.2 equiv.), 2-oxazolidinone (0.20 mmol, 17.8 mg, 2 equiv.) and 0.3 mL of dry and degassed acetonitrile were added. The mixture was allowed to stir at room temperature (21°C) for 24 hours. The crude product was purified with an automated column chromatography (pentane/EtOAc, 2:3), affording 11.7 mg of the product **11**, 54% yield.

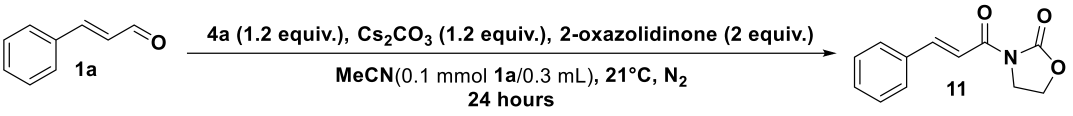

4. 1 mmol scale synthesis of 3aa

To a 5 ml V-vial equipped with a stir-bar, cinnamaldehyde (1.00 mmol, 132.6 mg, 1 equiv.), Iron (II) phthalocyanine (0.03 mmol, 17.1 mg, 3 mol%), redox NHC **4a** (0.20 mmol, 94.6 mg, 20 mol%), potassium carbonate (0.50 mmol, 69.4 mg, 0.5 equiv.), alcohol (8 equiv.) and 2 ml of ethyl acetate were added. The vial was equipped with a lid with two holes (2 mm in diameter). The mixture was allowed to stir at room temperature (21°C) for 24 hours. The crude product was purified with an automated column chromatography (pentane/EtOAc, 9:1), evaporated under reduced pressure, and dried under vacuum affording 148.9 mg of **3aa**, corresponding to a 92% yield.

5. General procedure for the synthesis of redox active N-heterocyclic carbenes

Redox active NHCs were prepared by the method described by Grubbs et al[1] and Glorius et al.[2]

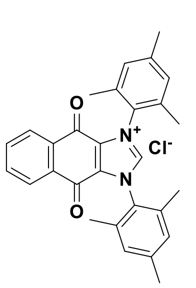

Redox NHC precursor **4a**: N,N'-Bis(2,4,6-trimethylphenyl)formamidine (4.0 mmol, 1.12 g, 2 equiv.) and 2,3- Dichloro-1,4-naphthoquinone (2.0 mmol, 454.1 mg, 1 equiv.) was mixed with 10 ml of acetonitrile in a 25 mL microwave vial equipped with a stir-bar and closed with a pressure lid. The mixture was heated to 110°C on a heating block on top of a heating plate for 48 hours. Subsequently, after the mixture had cooled, $NaHCO_3$ (2.0 mmol, 168.0 mg, 1 equiv.) was added and then heated to 60°C for 24 hours. After cooling, the acetonitrile was evaporated under reduced pressure and the crude was redissolved in dichloromethane. The mixture was filtered over celite and evaporated under reduced pressure until a minimal amount of dichloromethane was remaining in the flask before there is precipitate. To the dissolved mixture, diethyl ether was added to precipitate the catalyst. The precipitate was filtered and dried under vacuum to give the precatalyst **4a** as a yellow solid (780 mg, 83% yield). ^{1}H NMR (400 MHz, $CDCl_3$) δ = 12.68 (s, 1H), 8.15 (dd, *J* = 5.7, 3.3 Hz, 2H), 7.88 (dd, *J* = 5.7, 3.3 Hz, 2H), 7.08 (s, 4H), 2.37 (s, 6H), 2.19 (s, 12H). ^{13}C{^{1}H} NMR (101 MHz, $CDCl_3$) δ = 173.5, 148.0, 141.9, 135.8, 133.7, 131.8, 130.9, 130.2, 129.0, 127.9, 21.4, 18.1.

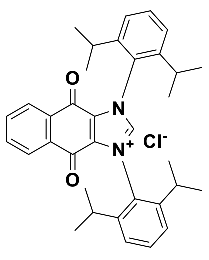

Redox NHC precursor **4b**: N,N'-Bis(2,6-diisopropylphenyl)formamidine (4.0 mmol, 1.45 g, 2 equiv.), 2,3-Dichloro-1,4-naphthoquinone (2.0 mmol, 452.3 mg, 1 equiv.) was mixed with 10 ml of acetonitrile in a 25 mL microwave vial equipped with a stirring bar and closed with a pressure lid**.** The mixture was heated to 110°C on a heating block on top of a heating plate for 48 hours. Subesquently, after the mixture had cooled, bicarbonate (2.0 mmol, 168.0 mg, 1 equiv.) was added and then heated to 60°C for 24 hours. After cooing, the acetonitrile was evaporated under reduced pressure and the crude was redissolved in dichloromethane. The mixture was filtered over celite and evaporated under reduced pressure until a minimal amount of dichloromethane to dissolve the crude was remaining in the flask. To the dissolved mixture, diethyl ether was added to precipitate the catalyst. The precipitate was filtered and dried under vacuum to give the precatalyst **4b** as a yellow solid (900 mg, 81%). ^{1}H NMR (800 MHz, $CDCl_3$) δ = 13.86 (s, 1H), 8.20 – 8.16 (m, 2H), 7.91 – 7.87 (m, 2H), 7.63 (t, J = 7.8 Hz, 2H), 7.40 (d, J = 7.8 Hz, 4H), 2.37 (septet, J = 6.9 Hz, 4H), 1.37 (d, J = 6.9 Hz, 12H), 1.19 (d, J = 6.9 Hz, 12H). ^{13}C{^{1}H} NMR (201 MHz, $CDCl_3$) δ = 173.5, 144.3, 135.8, 132.5, 132.0, 131.5, 128.5, 128.0, 124.9, 30.0, 24.9, 23.4.

6. Other NHC catalyzed reactions

6.1. Aerobic [3+3] cyclization using 4a

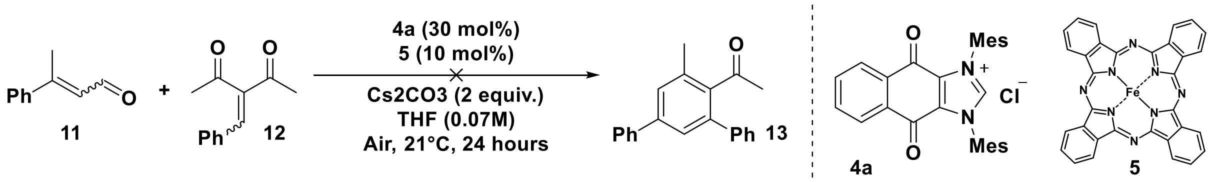

To a 5 ml V-vial equipped with a stir-bar, **12** (0.2 mmol, 37.6 mg, 1 equiv.), **5** (0.02 mmol, 11.4 mg, 10 mol%), redox NHC **4a** (0.06 mmol, 28.3 mg, 30 mol%), cesium carbonate (0.4 mmol, 130.3 mg, 2 equiv.), **11** (0.4 mmol, 58.5 mg, 2 equiv.) and 3 mL of tetrahydrofuran were added. The mixture was allowed to stir at room temperature (21°C) for 24 hours. The reaction did not proceed under the described conditions.

6.2. Aerobic [2+4] cyclization using 4a

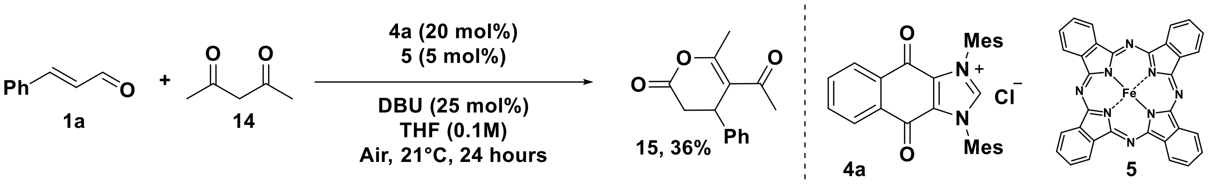

To a 5 ml V-vial equipped with a stir-bar, **1a** (0.3 mmol, 39.7 mg, 1 equiv.), **5** (0.015 mmol, 8.5 mg, 5 mol%), redox NHC **4a** (0.06 mmol, 28.3 mg, 20 mol%), DBU (0.075 mmol, 11.4 mg, 0.25 equiv.), **14** (0.42mmol, 42.1 mg, 1.4 equiv.) and 3 mL of tetrahydrofuran were added. The mixture was allowed to stir at room temperature (21°C) for 24 hours. The yield was quantified by performing a quantitative NMR experiment in $CDCl_3$ and with dimethyl sulfone as internal standard, giving 36% yield of **15**.

7. NMR characterization of compounds

Methyl cinnamate (**3aa**)[3]

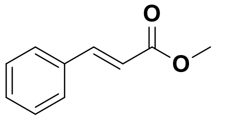

3aa

White solid (36.9 mg, 90%). Purified with automated column chromatography (pentane/EtOAc, 9:1). ^{1}H NMR (600 MHz, $CDCl_3$) δ = 7.70 (d, J = 16.0 Hz, 1H), 7.55 – 7.49 (m, 2H), 7.40 – 7.35 (m, 3H), 6.45 (d, J = 16.0 Hz, 1H), 3.81 (s, 3H). ^{13}C{^{1}H} NMR (151 MHz, $CDCl_3$) δ = 167.5, 145.0, 134.5, 130.4, 129.0, 128.2, 117.9, 51.8.

Methyl 4-chlorocinnamate (**3ba**)[3]

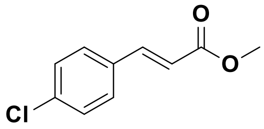

3ba

White solid (43 mg, 87%). Purified with automated column chromatography (pentane/EtOAc, 9:1). ^{1}H NMR (600 MHz, $CDCl_3$) δ = 7.63 (d, *J* = 16.0 Hz, 1H), 7.44 (d, *J* = 8.5 Hz, 2H), 7.34 (d, *J* = 8.5 Hz, 1H), 6.40 (d, *J* = 16.0 Hz, 1H), 3.80 (s, 3H). ^{13}C{^{1}H} NMR (151 MHz, $CDCl_3$) δ = 167.3, 143.5, 136.3, 133.0, 129.3, 129.3, 118.5, 51.9.

Methyl 4-fluorocinnamate (**3ca**)[3]

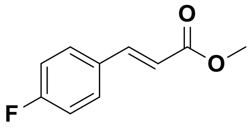

3ca

White solid (37.7 mg, 84%). Purified with automated column chromatography (pentane/EtOAc, 9:1). ^{1}H NMR (600 MHz, $CDCl_3$) δ = 7.64 (d, *J* = 16.0 Hz, 1H), 7.52 – 7.47 (m, 2H), 7.11 – 7.03 (m, 2H), 6.35 (d, *J* = 16.0 Hz, 1H), 3.79 (s, 3H). ^{13}C{^{1}H} NMR (151 MHz, $CDCl_3$) δ = 167.4, 164.0 (d, $^1J_{C-F}$ = 251.4 Hz), 143.6, 130.7, 130.0 (d, $^3J_{C-F}$ = 8.4 Hz), 117.7, 116.1 (d, $^2J_{C-F}$ = 21.9 Hz), 51.8. ^{19}F NMR (282 MHz, $CDCl_3$) δ = 109.9 ppm.

Methyl 2-Bromocinnamate (**3da**)[4]

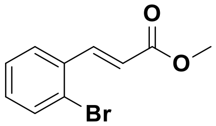

3da

Colorless oil (50 mg, 83%). Purified using automated column chromatography (pentane/EtOAc, 9:1). ^{1}H NMR (700 MHz, $CDCl_3$) δ = 8.04 (d, *J* = 15.9 Hz, 1H), 7.62 – 7.57 (m, 2H), 7.35 – 7.30 (m, 1H), 7.23 – 7.20 (m, 1H), 6.38 (d, *J* = 15.9 Hz, 1H), 3.82 (s, 3H). ^{13}C{^{1}H} NMR (176 MHz, $CDCl_3$) δ = 166.9, 143.3, 134.6, 133.5, 131.3, 127.9, 127.8, 125.4, 120.8, 52.0.

Methyl 4-methoxycinnamate (**3ea**)[3]

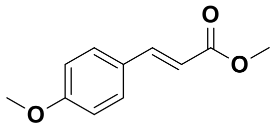

3ea

Pale yellow solid (29.8 mg, 62%). Purified with automated column chromatography (pentane/EtOAc, 9:1). ^{1}H NMR (600 MHz, $CDCl_3$) δ = 7.65 (d, *J* = 16.0 Hz, 1H), 7.47 (d, *J* = 8.7 Hz, 2H), 6.90 (d, *J* = 8.8 Hz, 1H), 6.31 (d, *J* = 16.0 Hz, 1H), 3.83 (s, 3H), 3.79 (s, 3H).^{13}C{^{1}H} NMR (151 MHz, $CDCl_3$) δ = 167.9, 161.5, 144.6, 129.8, 127.2, 115.4, 114.4, 55.5, 51.7.

Isoamyl 4-methoxycinnamate (**3eb**)[3]

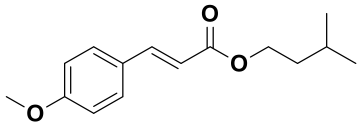

3eb

Yellow oil (31.6 mg, 51%). Purified using automated column chromatography (pentane/EtOAc, 9:1). ^{1}H NMR (600 MHz, $CDCl_3$) δ = 7.63 (d, J = 16.0 Hz, 1H), 7.47 (d, J = 8.7 Hz, 2H), 6.90 (d, J = 8.7 Hz, 2H), 6.30 (d, J = 16.0 Hz, 1H), 4.22 (t, J = 6.9 Hz, 2H), 3.83 (s, 3H), 1.80 – 1.70 (m, 1H), 1.59 (q, J = 6.9 Hz, 2H), 0.95 (d, J = 6.7 Hz, 6H ^{13}C{^{1}H} NMR (151 MHz, $CDCl_3$) δ = 167.6, 161.4, 144.3, 129.8, 127.3, 115.9, 114.4, 63.2, 55.5, 37.6, 25.2, 22.6.

Methyl 2-nitrocinnamate (**3fa**)[3]

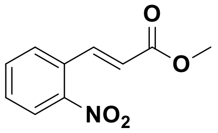

3fa

White solid (51 mg, 98%). Purified using automated column chromatography (pentane/EtOAc, 9:1). ^{1}H NMR (600 MHz, $CDCl_3$) δ = 8.10 (d, J = 15.8 Hz, 1H), 8.04 – 8.01 (m, 1H), 7.67 – 7.60 (m, 2H), 7.57 – 7.51 (m, 1H), 6.35 (d, J = 15.8 Hz, 1H), 3.81 (s, 3H). ^{13}C{^{1}H} NMR (151 MHz, $CDCl_3$) δ = 166.3, 148.4, 140.2, 133.7, 130.6, 130.4, 129.2, 125.0, 122.9, 52.1.

Phenyl cinnamate (**3ac**)[5]

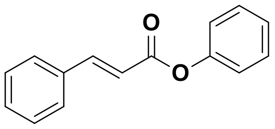

3ac

Colorless liquid (53.1 mg, 94%). Purified using automated column chromatography (pentane:EtOAc, 9:1). ^{1}H NMR (600 MHz, $CDCl_3$) δ = 7.88 (d, J = 16.0 Hz, 1H), 7.66 – 7.53 (m, 2H), 7.49 – 7.34 (m, 5H), 7.37 – 7.22 (m, 1H), 7.23 – 7.12 (m, 2H), 6.64 (d, J = 16.0 Hz, 1H). ^{13}C{^{1}H} NMR (151 MHz, $CDCl_3$) δ = 165.5, 150.9, 146.7, 134.3, 130.8, 129.6, 129.1, 128.4, 125.9, 121.8, 117.4

4-methoxyphenyl cinnamate (**3ad**)[5]

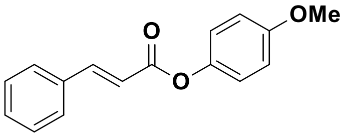

3ad

White solid (42.3 mg, 66%) Purified using automated column chromatography (pentane/EtOAc, 9:1). ^{1}H NMR (600 MHz, $CDCl_3$) δ = 7.86 (d, J = 16.0 Hz, 1H), 7.62 – 7.56 (m, 2H), 7.46 – 7.41 (m, 3H), 7.12 – 7.07 (m, 2H), 6.96 – 6.90 (m, 2H), 6.63 (d, J = 16.0 Hz, 1H), 3.82 (s, 3H). ^{13}C{^{1}H} NMR (151 MHz, $CDCl_3$) δ = 165.9, 157.4, 146.5, 144.4, 134.4, 130.8, 129.1, 128.4, 122.5, 117.5, 114.6, 55.8.

(2-oxo-1,3-dioxolan-4-yl)methyl cinnamate (**3ae**)[6]

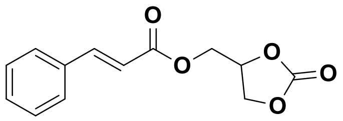

3ae

Colorless oil (49.1 mg, 78%). Purified using automated column chromatography (pentane/EtOAc, 9:1). ^{1}H NMR (600 MHz, $CDCl_3$) δ = 7.72 (d, *J* = 16.0 Hz, 1H), 7.56 – 7.50 (m, 2H), 7.44 – 7.35 (m, 3H), 6.45 (d, *J* = 16.0 Hz, 1H), 5.01 – 4.97 (m, 1H), 4.59 (m, 1H), 4.53 – 4.39 (m, 2H), 4.39 – 4.34 (m, 1H). ^{13}C{^{1}H} NMR (151 MHz, $CDCl_3$) δ = 166.3, 154.6, 146.7, 134.0, 130.9, 129.1, 128.4, 116.5, 74.0, 66.2, 63.2.

(6,6-Dimethylbicyclo(3.1.1)hept-2-en-2-yl)methyl cinnamate (**3af**)

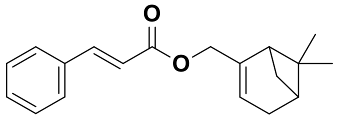

3af

Colorless oil (52.4 mg, 74%). Purified using automated column chromatography (pentane/EtOAc, 2:1). ^{1}H NMR (600 MHz, $CDCl_3$) δ = 7.69 (d, *J* = 16.0 Hz, 1H), 7.56 – 7.49 (m, 2H), 7.42 – 7.35 (m, 3H), 6.45 (d, *J* = 16.0 Hz, 1H), 5.62 (s, 1H), 4.59 (m, 2H), 2.43 (m, 1H), 2.38 – 2.23 (m, 2H), 2.18 (m, 1H), 2.15 – 2.09 (m, 1H), 1.31 (s, 3H), 1.28 – 1.20 (m, 1H), 0.86 (s, 3H). ^{13}C{^{1}H} NMR (151 MHz, $CDCl_3$) δ = 167.0, 144.8, 143.2, 134.6, 130.4, 129.0, 128.2, 121.6, 118.3, 67.2, 43.8, 40.9, 38.2, 31.6, 31.4, 26.3, 21.2. HRMS (ESI) m/z [M + Na]$^+$ Calcd for $C_{19}H_{22}O_2$ 283.1698; Found 283.1692. IR (ATR): 2915, 2328, 1711, 1636, 1577, 1559, 1469, 1449, 1366, 1304, 1251, 1201, 1157, 978, 887, 863, 802, 766, 710, 684, 572, 507, 483 cm^{-1}.

Methyl nicotinate (**3ga**)[7]

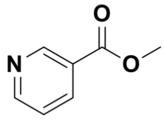

3ga

White solid (27.4 mg, 79%). Purified using automated column chromatography (pentane/Et_2O, 1:4). ^{1}H NMR (600 MHz, $CDCl_3$) δ = 9.21 (d, *J* = 1.4 Hz, 1H), 8.78 – 8.74 (m, 1H), 8.30 – 8.26 (m, 1H), 7.40 – 7.36 (m, 1H), 3.94 (s, 3H). ^{13}C{^{1}H} NMR (151 MHz, $CDCl_3$) δ = 165.9, 153.5, 151.0, 137.1, 126,1 123.4, 52.5.

Methyl 2-naphthoate (**3ha**)[8]

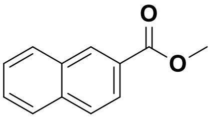

3ha

White solid (29.3 mg, 63%). Purified using automated column chromatography (pentane/EtOAc, 9:1). ^{1}H NMR (700 MHz, $CDCl_3$) δ = 8.62 (s, 1H), 8.07 (d, *J* = 8.6 Hz, 1H), 7.95 (d, *J* = 8.1 Hz, 1H), 7.88 (d, *J* = 8.4 Hz, 2H), 7.61 – 7.52 (m, 2H), 3.99 (s, 3H). ^{13}C{^{1}H} NMR (176 MHz, $CDCl_3$) δ = 167.4, 135.6, 132.6, 131.2, 129.5, 128.4, 128.3, 127.9, 127.5, 126.8, 125.3, 52.3.

Methyl 4-bromobenzoate (**3ia**)[9]

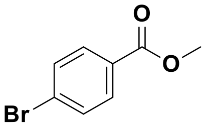

3ia

White solid (53.1 mg, 98%). Purified using automated column chromatography (EtO_2, Pentane 1:4), ^{1}H NMR (600 MHz, $CDCl_3$) δ = 7.89 (d, J = 8.5 Hz, 2H), 7.57 (d, J = 8.5 Hz, 2H), 3.91 (s, 3H). ^{13}C{^{1}H} NMR (151 MHz, $CDCl_3$) δ = 166.5, 131.8, 131.2, 129.2, 128.6, 52.4.

Methyl 3-bromobenzoate (**3ja**)[9]

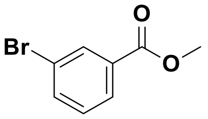

3ja

White solid (49 mg, 91%). Purified using automated column chromatography (pentane/EtOAc, 9:1) ^{1}H NMR (600 MHz, $CDCl_3$) δ = 8.18 (t, *J* = 1.8 Hz, 1H), 7.97 (dt, *J* = 7.8, 1.3 Hz, 1H), 7.68 (ddd, *J* = 7.9, 2.2, 1.1 Hz, 1H), 7.32 (t, *J* = 7.9 Hz, 1H), 3.93 (s, 3H). ^{13}C{^{1}H} NMR (151 MHz, $CDCl_3$) δ = 165.9, 136.0, 132.8, 132.2, 130.1, 128.3, 122.6, 52.6.

Methyl 2-chlorobenzoate (**3ka**)[10]

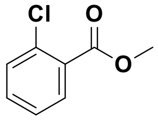

3ka

Colorless liquid (33 mg, 77%). Purified using automated column chromatography (pentane/EtOAc, 9:1). ^{1}H NMR (600 MHz, $CDCl_3$) δ = 7.82 (d, *J* = 7.8 Hz, 1H), 7.47 – 7.43 (m, 1H), 7.43 – 7.39 (m, 1H), 7.33 – 7.28 (m, 1H), 3.93 (s, 3H). ^{13}C{^{1}H} NMR (151 MHz, $CDCl_3$) δ = 166.3, 133.8, 132.7, 131.5, 131.2, 130.2, 126.7, 52.5.

Methyl 5-bromo-2-methoxybenzoate (**3la**)[11]

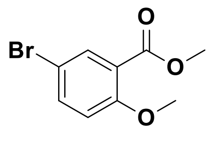

3la

White solid (52 mg, 85%). Purified using automated column chromatography (pentane/EtOAc, 9:1), ^{1}H NMR (600 MHz, $CDCl_3$) δ = 7.89 (d, *J* = 2.6 Hz, 1H), 7.53 (dd, *J* = 8.9, 2.6 Hz, 1H), 6.85 (d, *J* = 8.9 Hz, 1H), 3.87 (s, 3H), 3.87 (s, 3H). ^{13}C{^{1}H} NMR (151 MHz, $CDCl_3$) δ = 165.4, 158.3, 136.2, 134.3, 121.8, 114.0, 112.3, 56.4, 52.4.

Methyl 4-cyanobenzoate (**3ma**)[9]

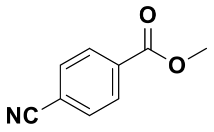

3ma

White solid (40 mg, 99%) Purified using automated column chromatography (pentane:EtOAc, 9:1). ^{1}H NMR (700 MHz, $CDCl_3$) δ = 8.14 – 8.10 (m, 2H), 7.74 – 7.71 (m, 2H), 3.94 (s, 3H). ^{13}C{^{1}H} NMR (176 MHz, $CDCl_3$) δ = 165.5, 134.0, 132.3, 130.2, 118.0, 116.5, 52.8.

Pyrrolidinyl cinnamate (**9**)[12]

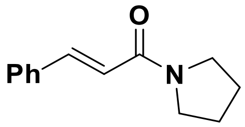

9

Beige solid (47 mg, 78%). Purified using automated column chromatography (pentane/EtOAc, 2:3). ^{1}H NMR (600 MHz, $CDCl_3$) δ = 7.71 – 7.66 (m, 1H), 7.54 – 7.50 (m, 2H), 7.38 – 7.33 (m, 2H), 6.75 – 6.70 (m, 1H), 3.64 – 3.55 (m, 4H), 2.02 – 1.95 (m, 2H), 1.91 – 1.85 (m, 2H). ^{13}C{^{1}H} NMR (151 MHz, $CDCl_3$) δ = 164.8, 141.7, 135.4, 129.6, 128.8, 127.9, 119.0, 46.7, 46.1, 26.2, 24.4.

3-cinnamoyloxazolidin-2-one (**11**)[13]

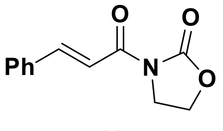

11

Beige solid (12 mg, 54%). Purified using automated column chromatography (pentane/EtOAc, 2:3). ^{1}H NMR (600 MHz, $CDCl_3$) δ = 7.94 – 7.84 (m, 2H), 7.66 – 7.59 (m, 2H), 7.43 – 7.36 (m, 4H), 4.46 (t, *J* = 8.5 Hz, 2H), 4.14 (t, *J* = 8.5 Hz, 2H). ^{13}C{^{1}H} NMR (151 MHz, $CDCl_3$) δ = 165.5, 153.7, 146.5, 134.7, 130.8, 129.0, 128.8, 116.7, 62.2, 43.0.

Dimethyl terephthalate (**3na**)[14]

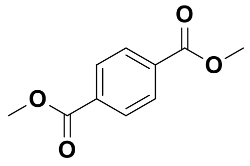

3na

To a 1 ml V-vial equipped with a stirring bar, terephthalaldehyde (0.25 mmol, 33.5 mg, 1 equiv.), Iron (II) phthalocyanine (0.015 mmol, 8.6 mg, 6 mol%.), redox NHC **4a** (0.10 mmol, 47.1 mg, 40 mol%), potassium carbonate (0.25 mmol, 34.6 mg, 1 equiv.), methanol (4.00 mmol, 128.2 mg, 16 equiv.) and 0.5 ml of ethyl acetate were added. The vial was equipped with a lid with two holes (2 mm in diameter). The mixture was allowed to stir at 21°C for 24 hours. The crude product was purified with an automated column chromatography (pentane/EtOAc, 9:1), affording 38.9 mg of the product **3na** as a white solid in 80% yield. ^{1}H NMR (600 MHz, $CDCl_3$) δ = 8.08 (s, 4H), 3.93 (s, 6H). ^{13}C{^{1}H} NMR (151 MHz, $CDCl_3$) δ = 166.4, 134.0, 129.7, 52.5.

Terephthalate oligomer (**3ng**)[15]

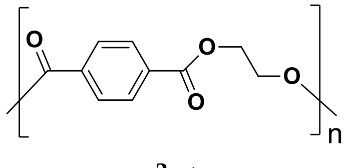

3ng

To a 5 ml V-vial equipped with a stir-bar, terephthalaldehyde (0.50 mmol, 67.1 mg, 1 equiv.), Iron (II) phthalocyanine (0.015 mmol, 8.6 mg, 6 mol%.), redox NHC **4a** (0.10 mmol, 47.1 mg, 20 mol%), 1,8-diazabicyclo[5.4.0]undec-7-ene (0.125 mmol, 19.0 mg, 0.25 equiv.), ethylene glycol (0.50 mmol, 32.7 mg, 1 equiv.) and 3mL of tetrahydrofuran were added. The vial was equipped with a lid with two holes (2 mm in diameter). The mixture was allowed to stir at 21°C for 24 hours. The crude product was purified by evaporation of the crude, followed by suspension in dichloromethane and filtering of the solids washing with dichloromethane on the frit filter. The solid was dissolved in dichloromethane and trifluoroacetic acid (4:1, v/v) and precipitated from methanol at 0°C. Filtration followed by washing with cold methanol and drying

under vacuum afforded 36.8 mg of the product **3ng** as a beige solid in 38% yield. NMR (600 MHz, $CDCl_3$:TFA (4:1)) δ = 8.15 (s, 4H, Ar), 4.82 (m, 4H, -OCH_2CH_2O-), 4.62 (bs, 0.02H, terminal $COOCH_2$), 4.19 (bs, 0.02H, terminal CH_2OH). $^{13}C\{^1H\}$ NMR (151 MHz, $CDCl_3$:TFA (4:1)) δ = 168.1, 133.7, 130.4, 65.5 (terminal COO$\underline{C}$H2), 64.3, 63.6 (Terminal $\underline{C}$H2OH).

8. References

1. Kuhn, K. M.; Grubbs, R. H., A Facile Preparation of Imidazolinium Chlorides. *Org. Lett.* **2008,** *10*, 2075-2077.
2. Hirano, K.; Urban, S.; Wang, C.; Glorius, F., A modular synthesis of highly substituted imidazolium salts. *Org. Lett.* **2009,** *11*, 1019-1022.
3. Ta, L.; Axelsson, A.; Sundén, H., Attractive aerobic access to the α, β-unsaturated acyl azolium intermediate: oxidative NHC catalysis via multistep electron transfer. *Green Chem.* **2016,** *18*, 686-690.
4. Yuan, Y.-Q.; Guo, S.-R., Remarkably facile Heck reactions in aqueous two-phase system catalyzed by reusable Pd/C under ligand-free conditions. *Synth. Comm.* **2012,** *42*, 1059-1069.
5. Ruiz, D. M.; Romanelli, G. P.; Vázquez, P. G.; Autino, J. C., Preyssler catalyst: An efficient catalyst for esterification of cinnamic acids with phenols and imidoalcohols. *Appl. Catal.* **2010,** *374*, 110-119.
6. Axelsson, A.; Antoine-Michard, A.; Sundén, H., Organocatalytic valorisation of glycerol via a dual NHC-catalysed telescoped reaction. *Green Chem.* **2017,** *19*, 2477-2481.
7. An, J. H.; Kim, K. D.; Lee, J. H., Highly Chemoselective Deoxygenation of N-Heterocyclic N-Oxides Using Hantzsch Esters as Mild Reducing Agents. *The Journal of Org. Chem.* **2021,** *86*, 2876-2894.
8. Ismael, A.; Gevorgyan, A.; Skrydstrup, T.; Bayer, A., Renewable solvents for palladium-catalyzed carbonylation reactions. *Org. Process Res. Dev.* **2020,** *24*, 2665-2675.
9. Ghosh, T.; Chandra, P.; Mohammad, A.; Mobin, S. M., Benign approach for methyl-esterification of oxygenated organic compounds using TBHP as methylating and oxidizing agent. *Appl. Catal.* **2018,** *226*, 278-288.
10. Kaganovsky, L.; Gelman, D.; Rueck-Braun, K., Trans-chelating ligands in palladium-catalyzed carbonylative coupling and methoxycarbonylation of aryl halides. *J. Organomet. Chem.* **2010,** *695*, 260-266.
11. Nishii, Y.; Ikeda, M.; Hayashi, Y.; Kawauchi, S.; Miura, M., Triptycenyl sulfide: A practical and active catalyst for electrophilic aromatic halogenation using N-halosuccinimides. *J. Am. Chem. Soc.* **2019,** *142*, 1621-1629.
12. De Sarkar, S.; Studer, A., Oxidative Amidation and Azidation of Aldehydes by NHC Catalysis. *Org. Lett.* **2010,** *12*, 1992-1995.
13. Ta, L.; Axelsson, A.; Sundén, H., N-Acylation of Oxazolidinones via Aerobic Oxidative NHC Catalysis. *J. Org. Chem.* **2018,** *83*, 12261-12268.
14. Agrawal, M. K.; Adimurthy, S.; Ghosh, P. K., Oxidative esterification of benzaldehyde and deactivated aromatic aldehydes with N-bromosuccinimide-pyridine. *Synth. Comm.* **2012,** *42*, 2931-2936.
15. Ragno, D.; Di Carmine, G.; Brandolese, A.; Bortolini, O.; Giovannini, P. P.; Fantin, G.; Bertoldo, M.; Massi, A., Oxidative NHC-Catalysis as Organocatalytic Platform for the Synthesis of Polyester Oligomers by Step-Growth Polymerization. *Chem. Eur. J.* **2019,** *25*, 14701-14710.

^{1}H-NMR (600 MHz, $CDCl_3$) of **4a**

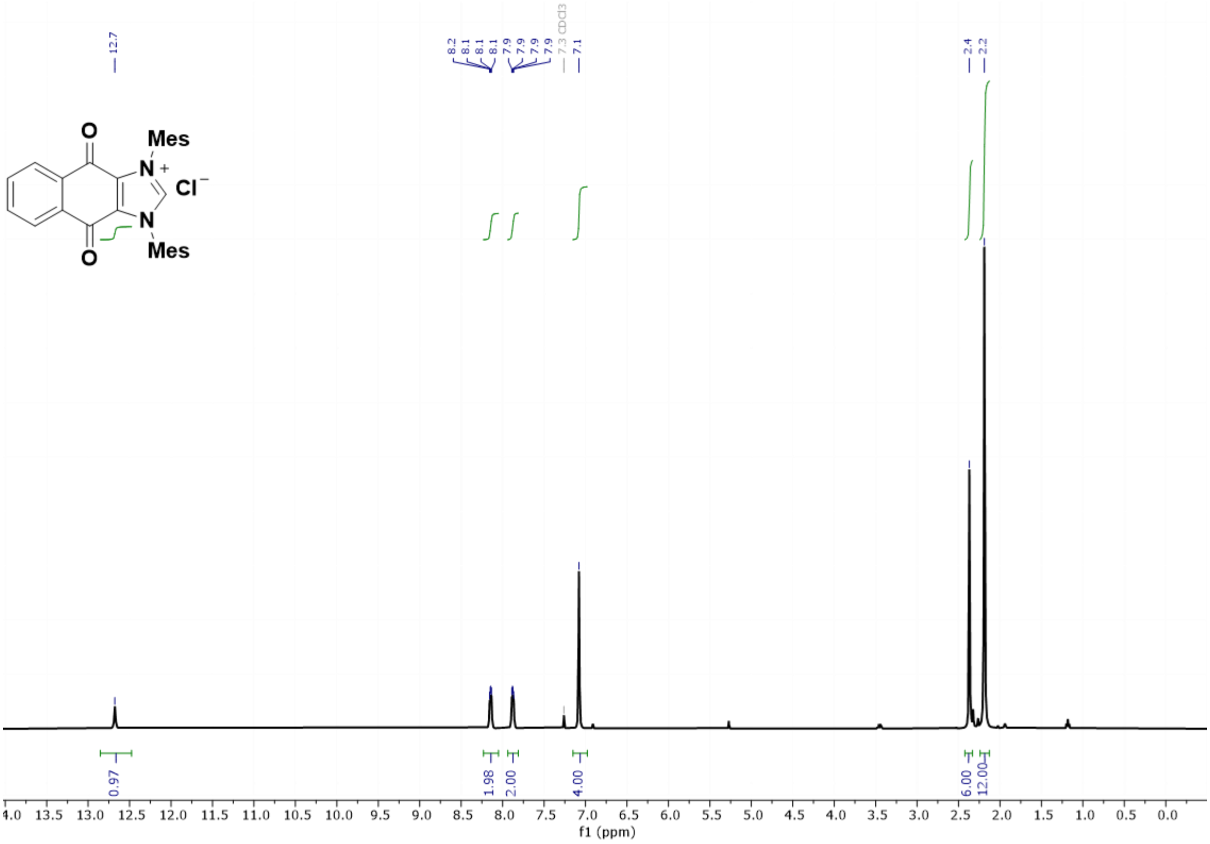

^{13}C-NMR (151 MHz, $CDCl_3$) of **4a**

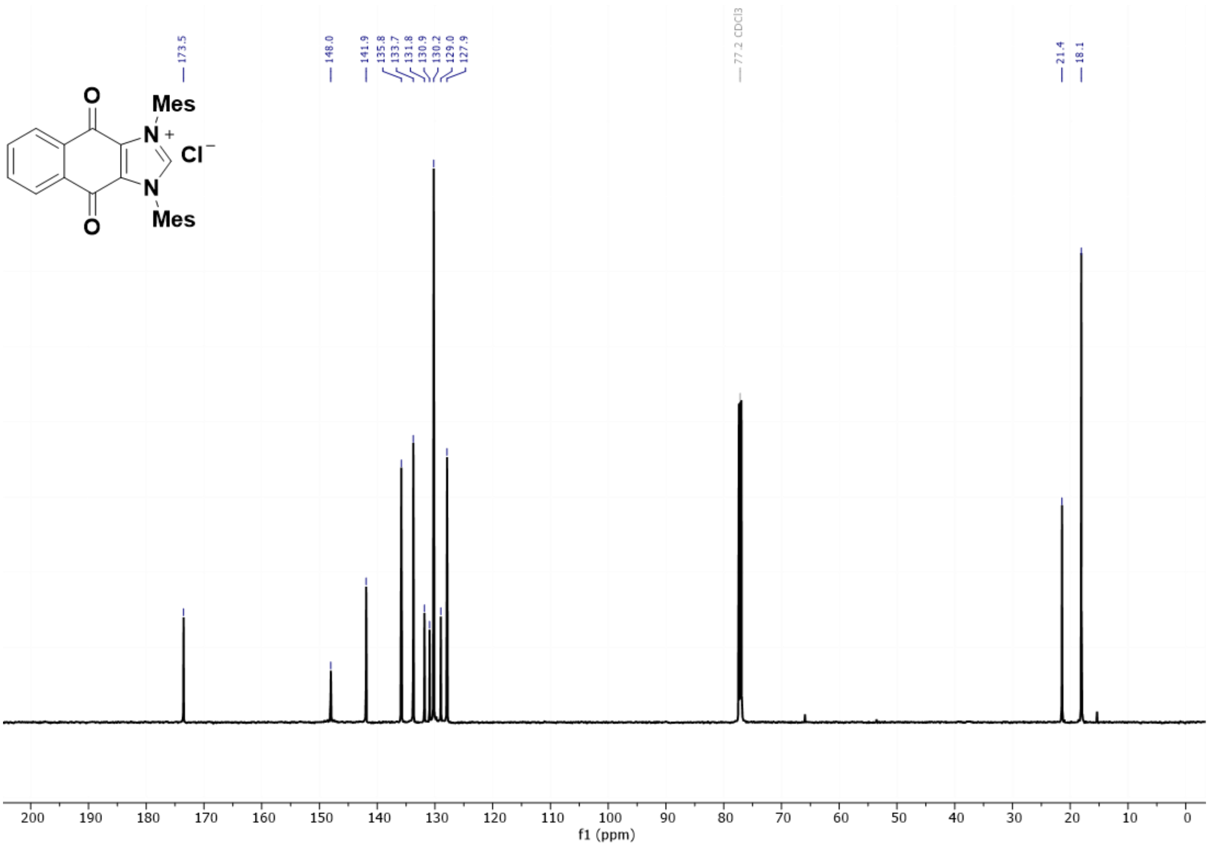

^{1}H-NMR (600 MHz, $CDCl_3$) of **4b**

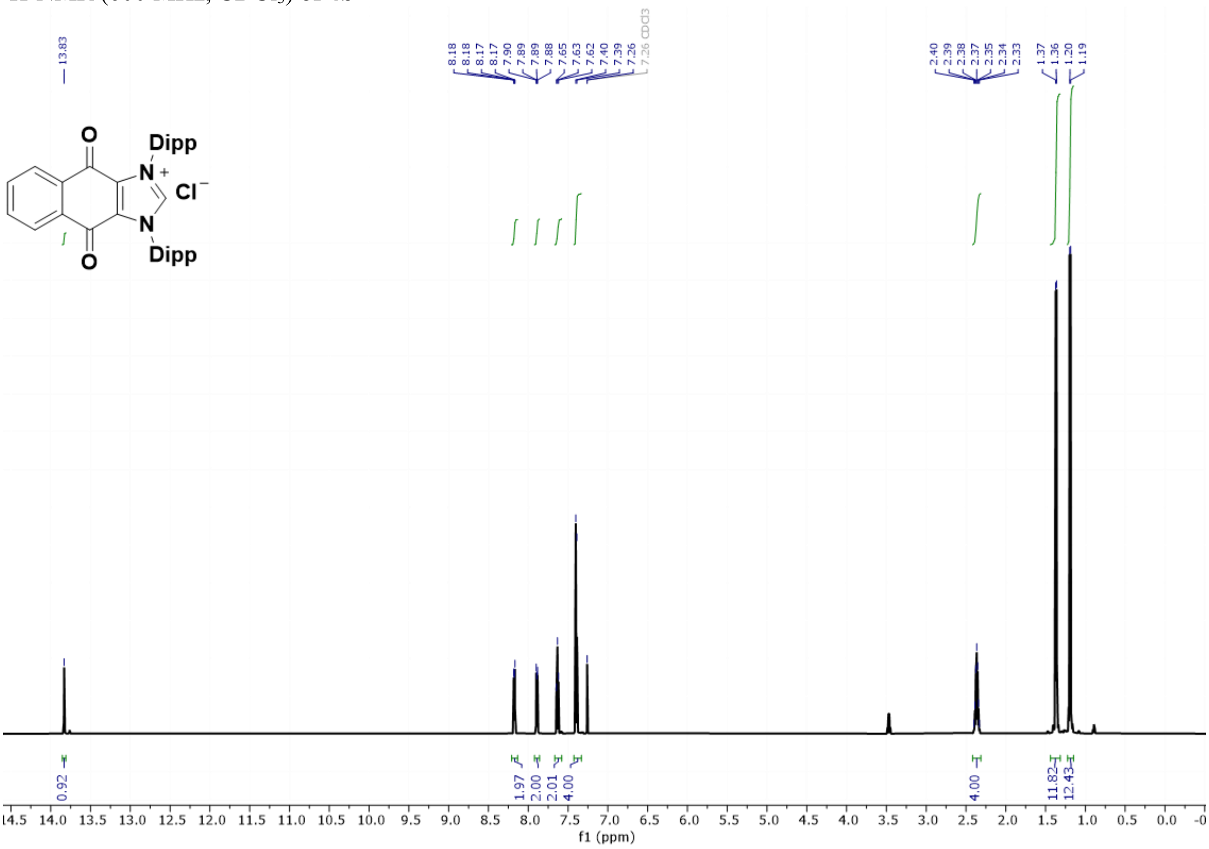

^{13}C-NMR (151 MHz, $CDCl_3$) of **4b**

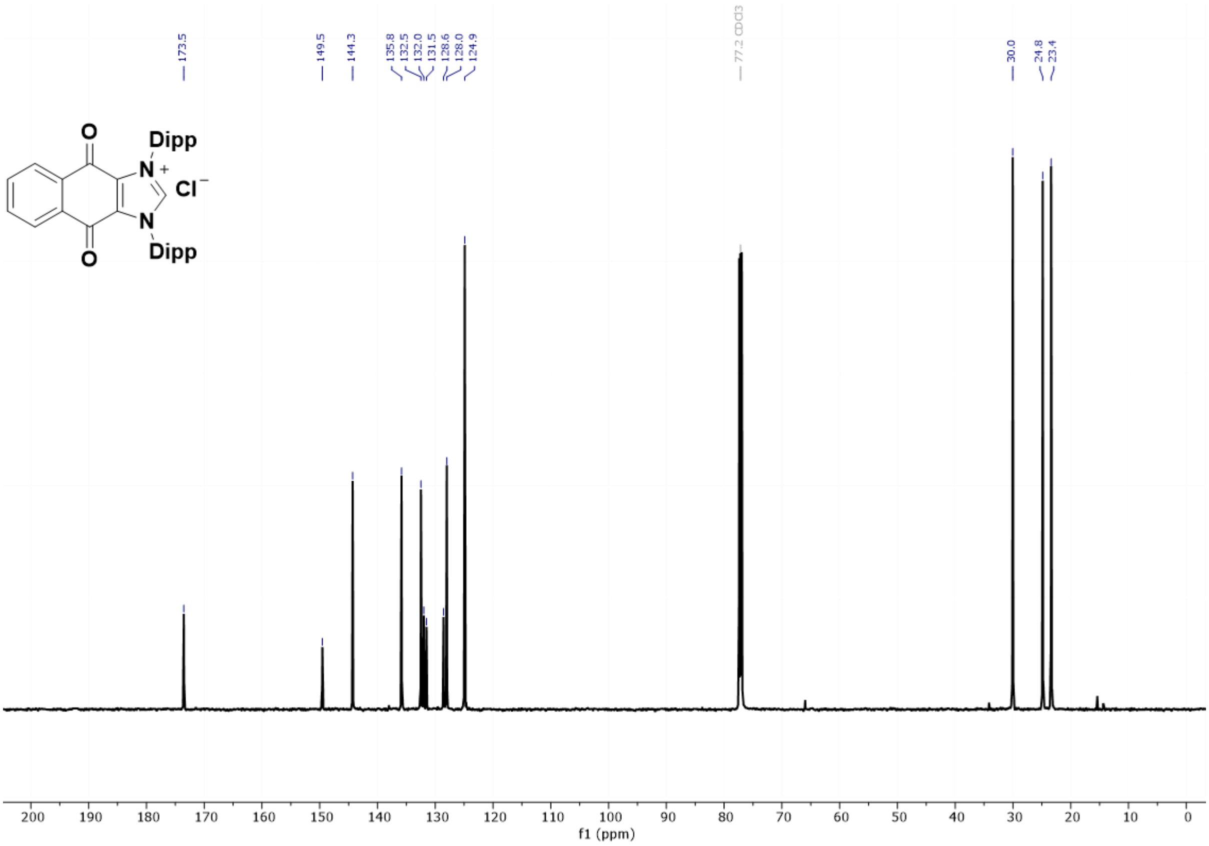

^{1}H-NMR (600 MHz, $CDCl_3$) of **3aa**

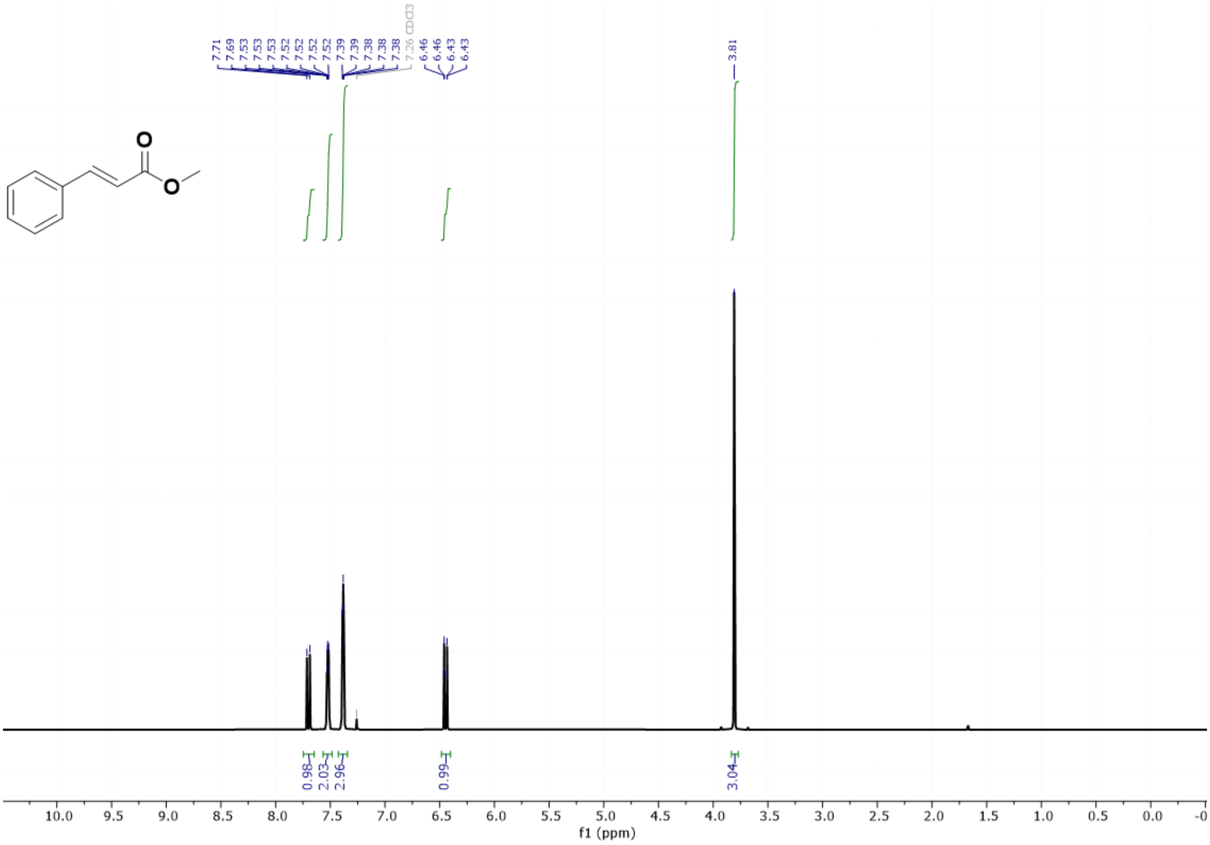

^{13}C-NMR (151 MHz, $CDCl_3$) of **3aa**

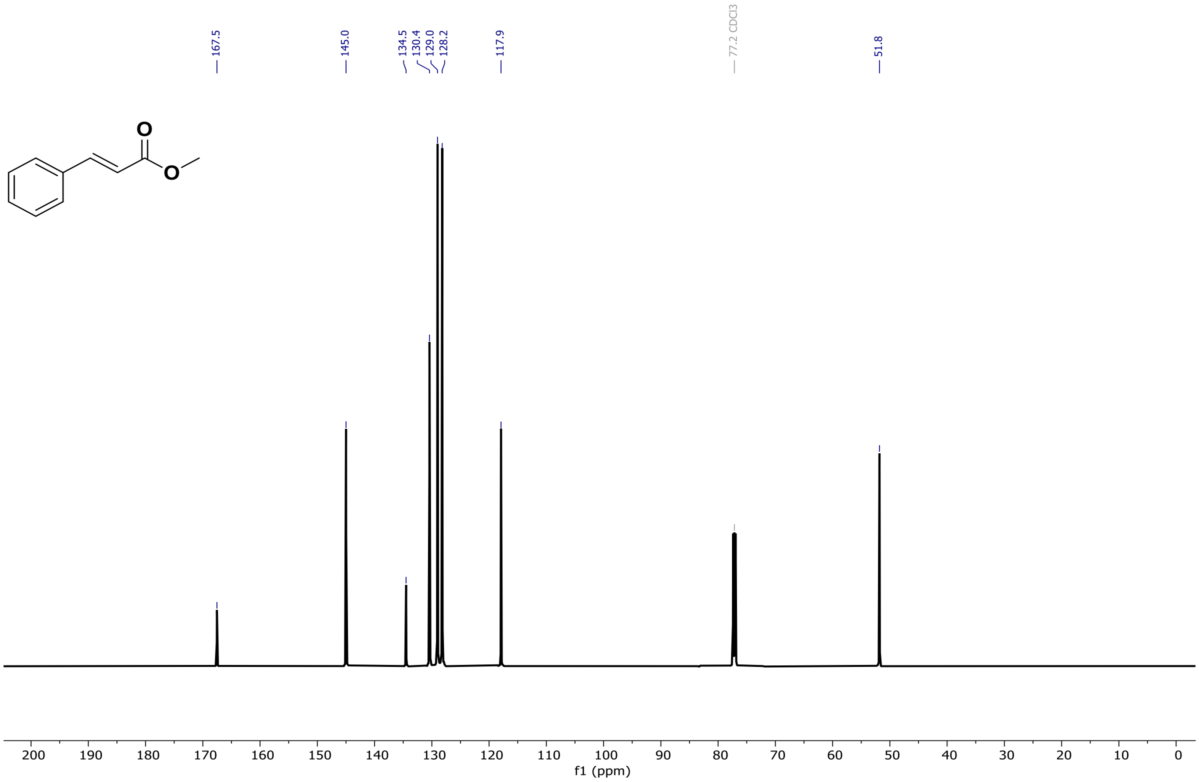

^{1}H-NMR (600 MHz, $CDCl_3$) of **3ba**

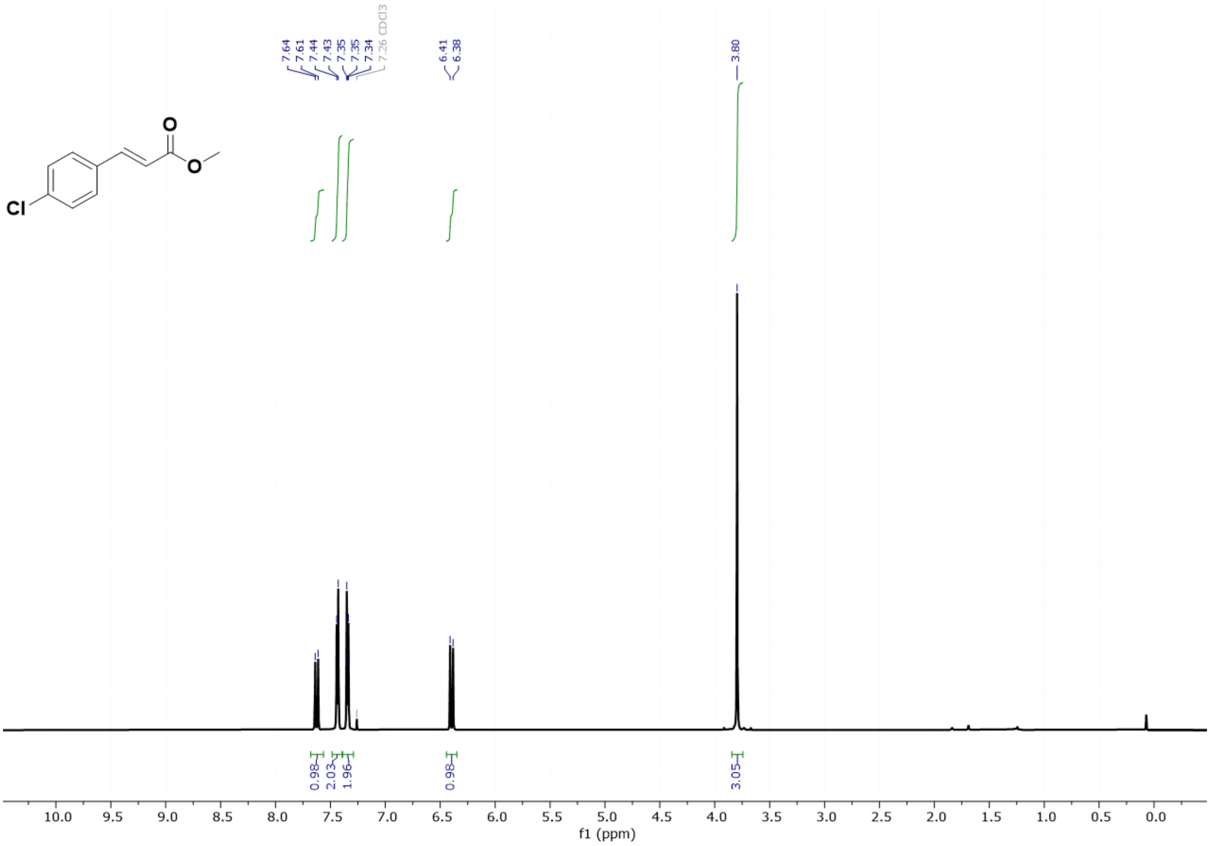

^{13}C-NMR (151 MHz, $CDCl_3$) of **3ba**

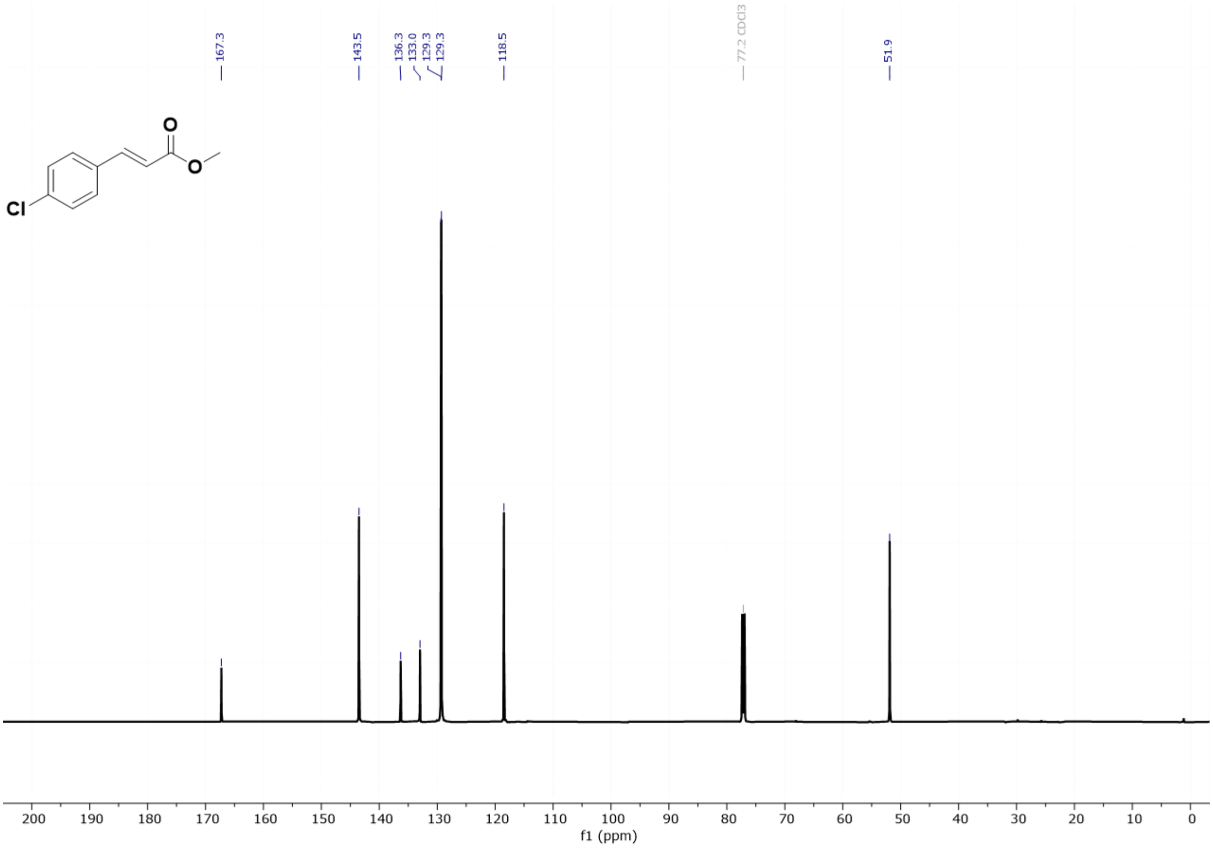

^{1}H-NMR (600 MHz, $CDCl_3$) of **3ca**

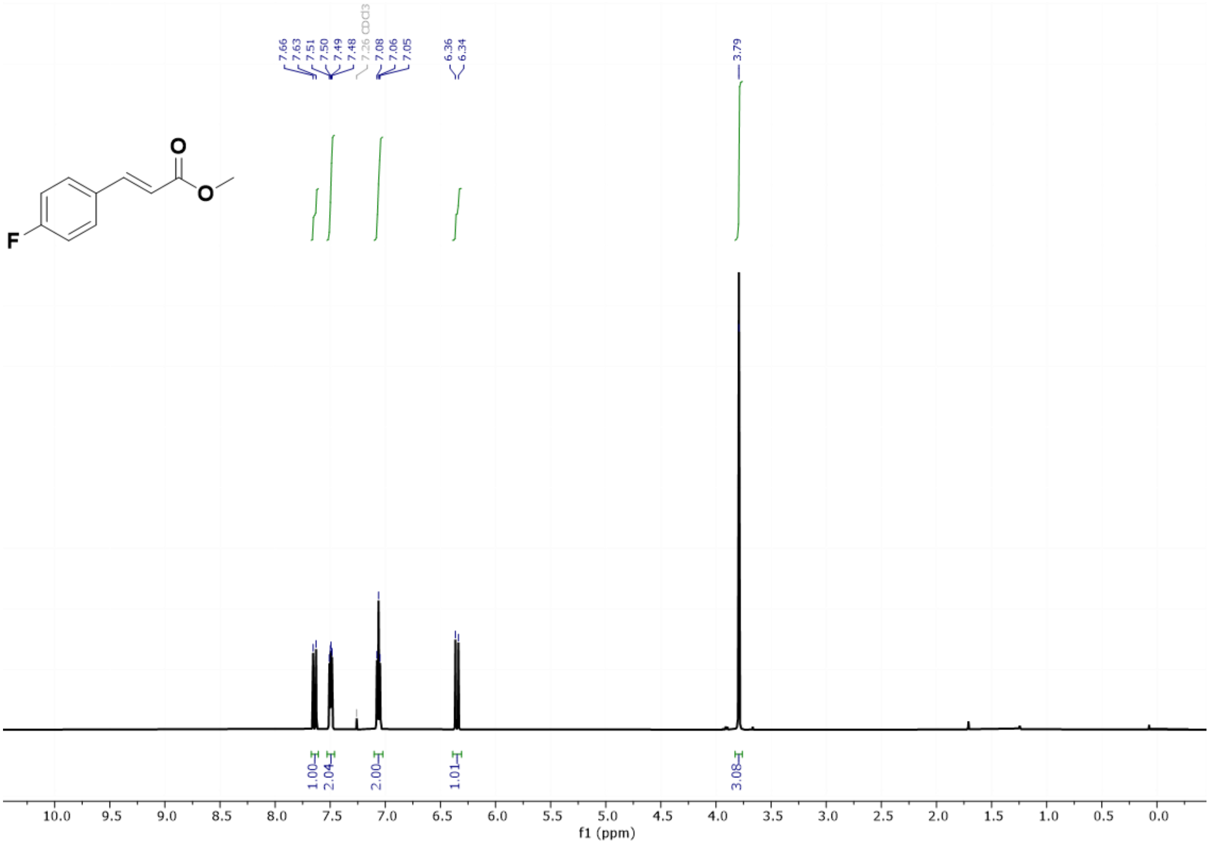

^{13}C-NMR (151 MHz, $CDCl_3$) of **3ca**

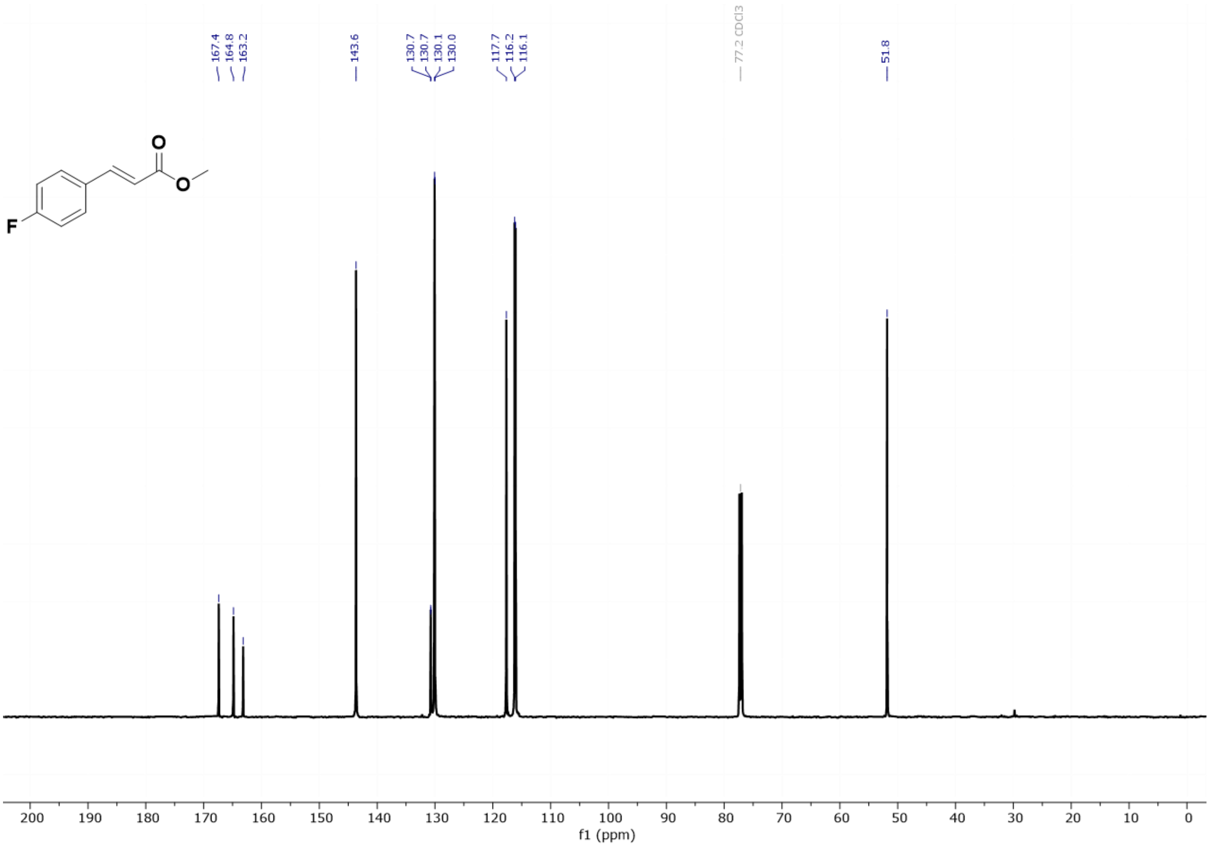

^{1}H-NMR (700 MHz, $CDCl_3$) of **3da**

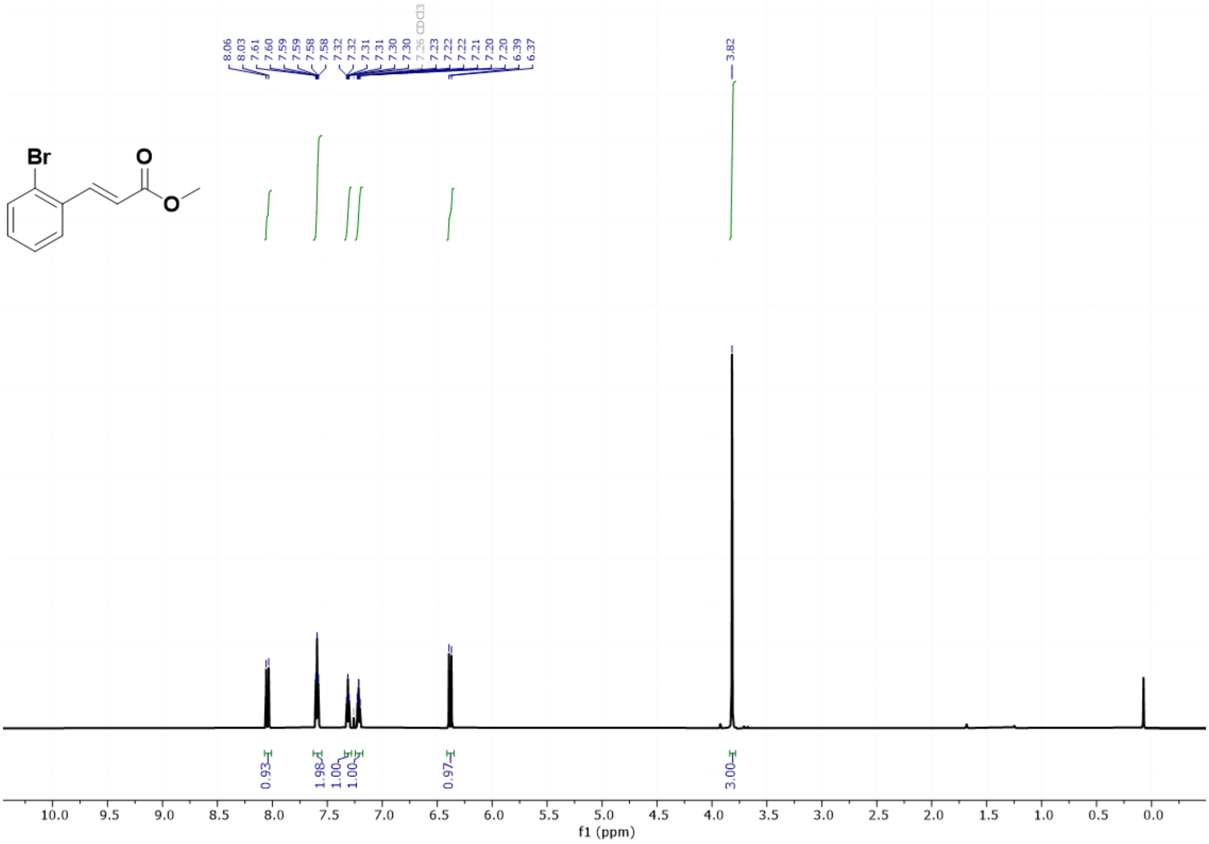

^{13}C-NMR (176 MHz, $CDCl_3$) of **3da**

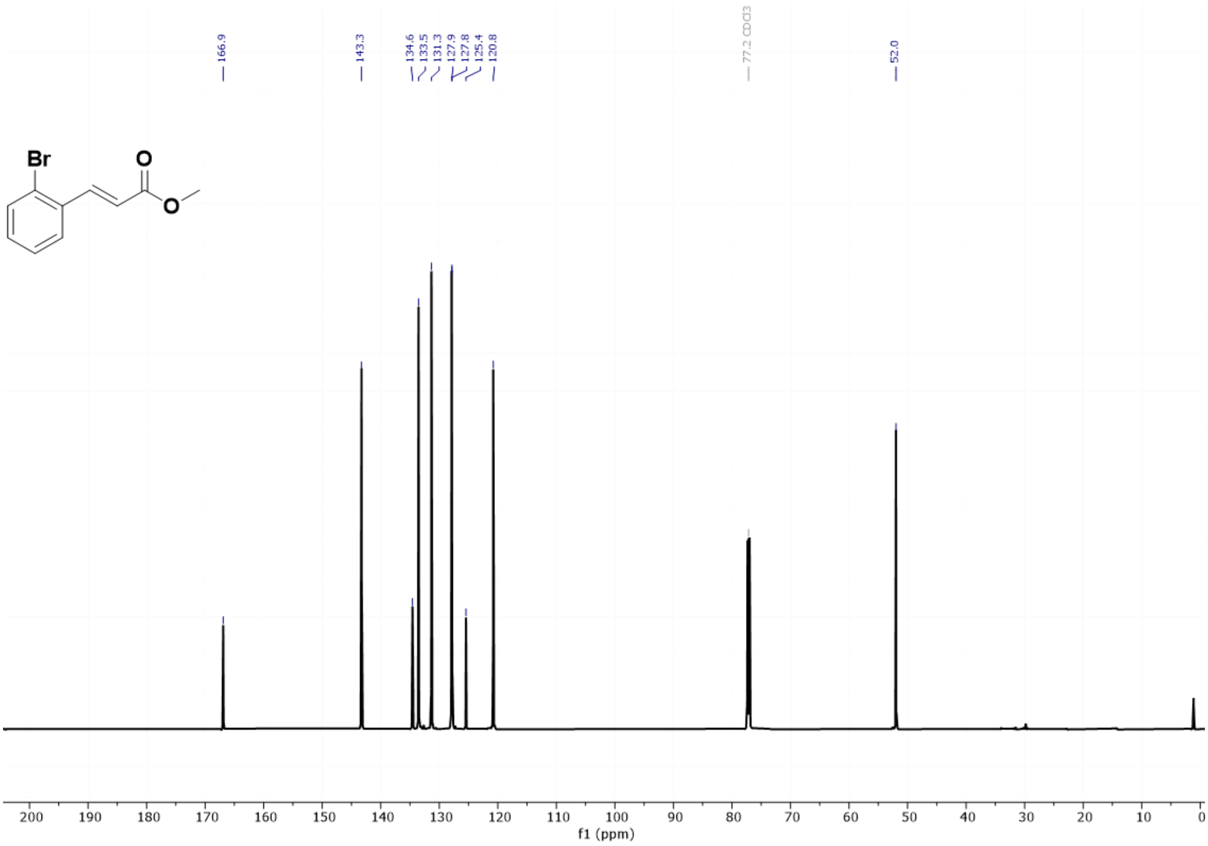

^{1}H-NMR (600 MHz, $CDCl_3$) of **3ea**

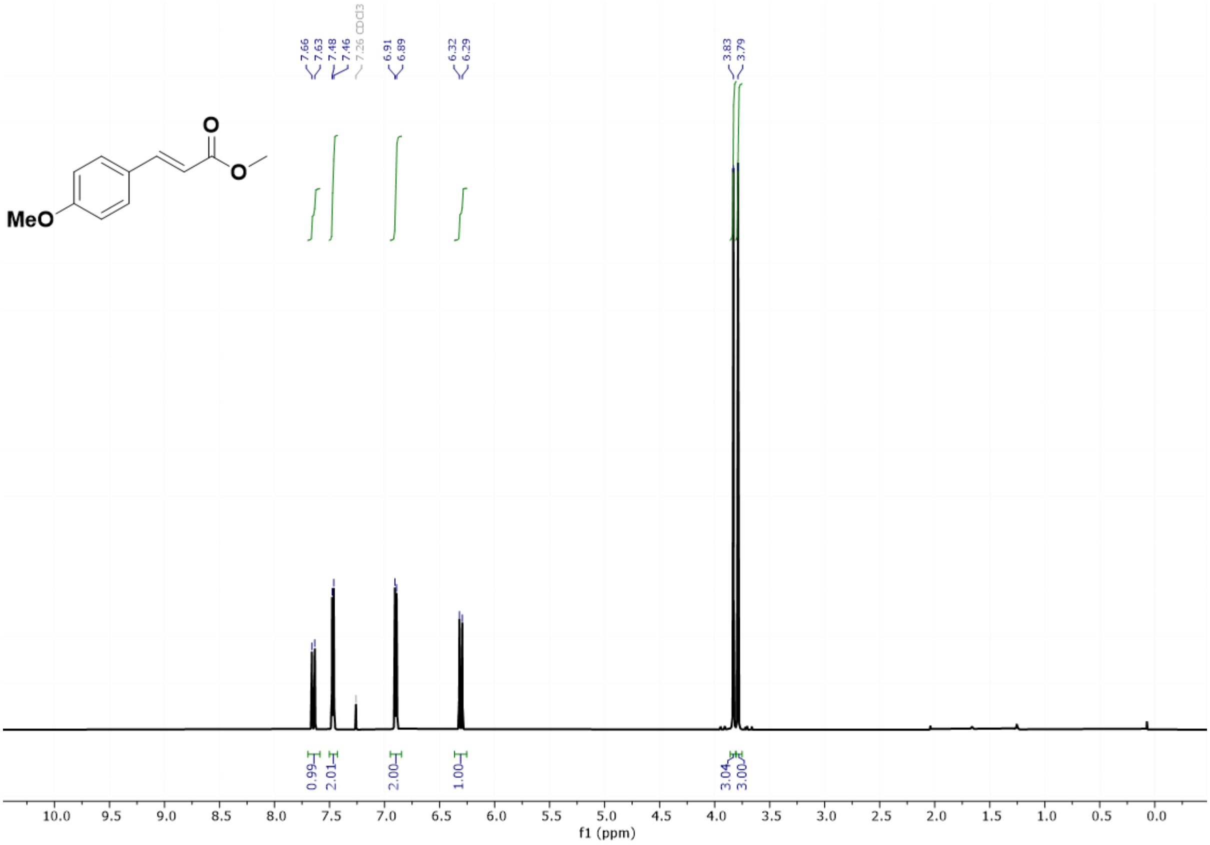

^{13}C-NMR (151 MHz, $CDCl_3$) of **3ea**

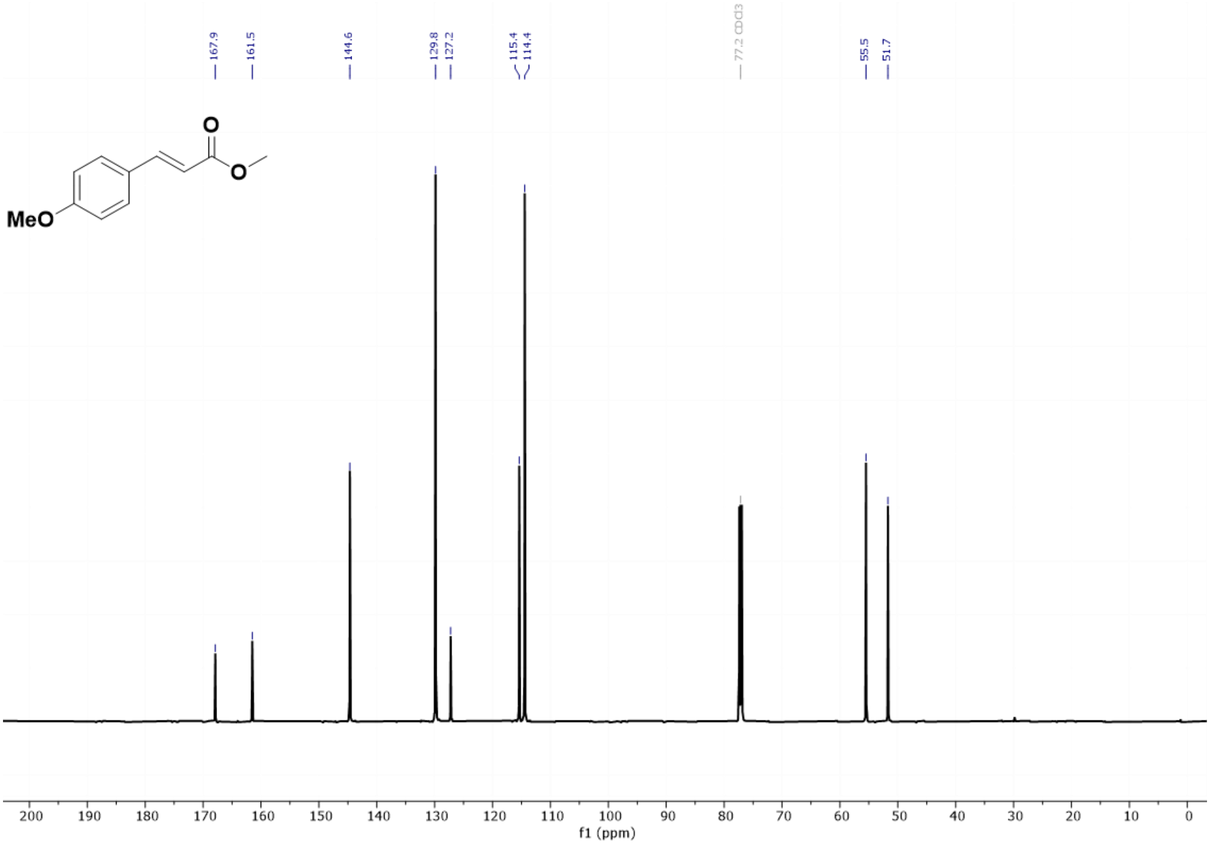

^{1}H-NMR (600 MHz, $CDCl_3$) of **3fb**

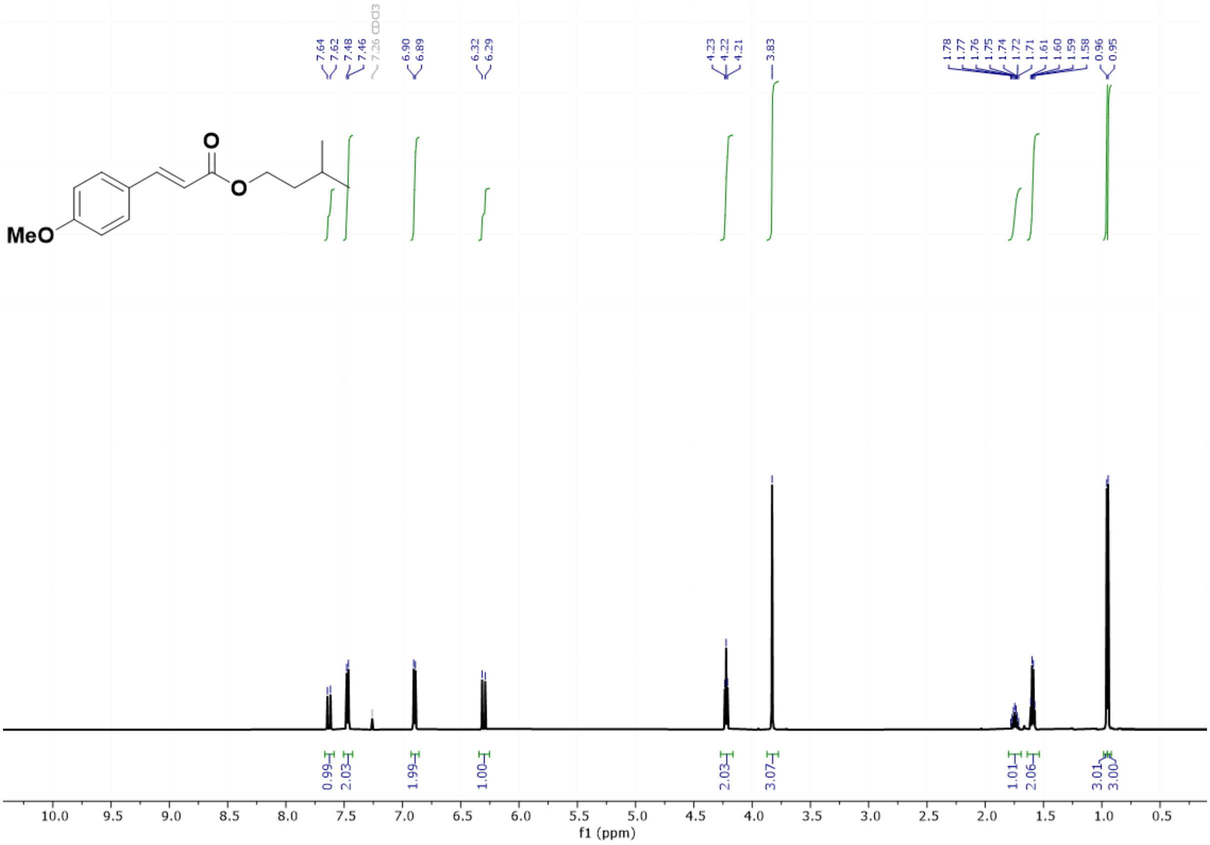

^{13}C-NMR (151 MHz, $CDCl_3$) of **3fb**

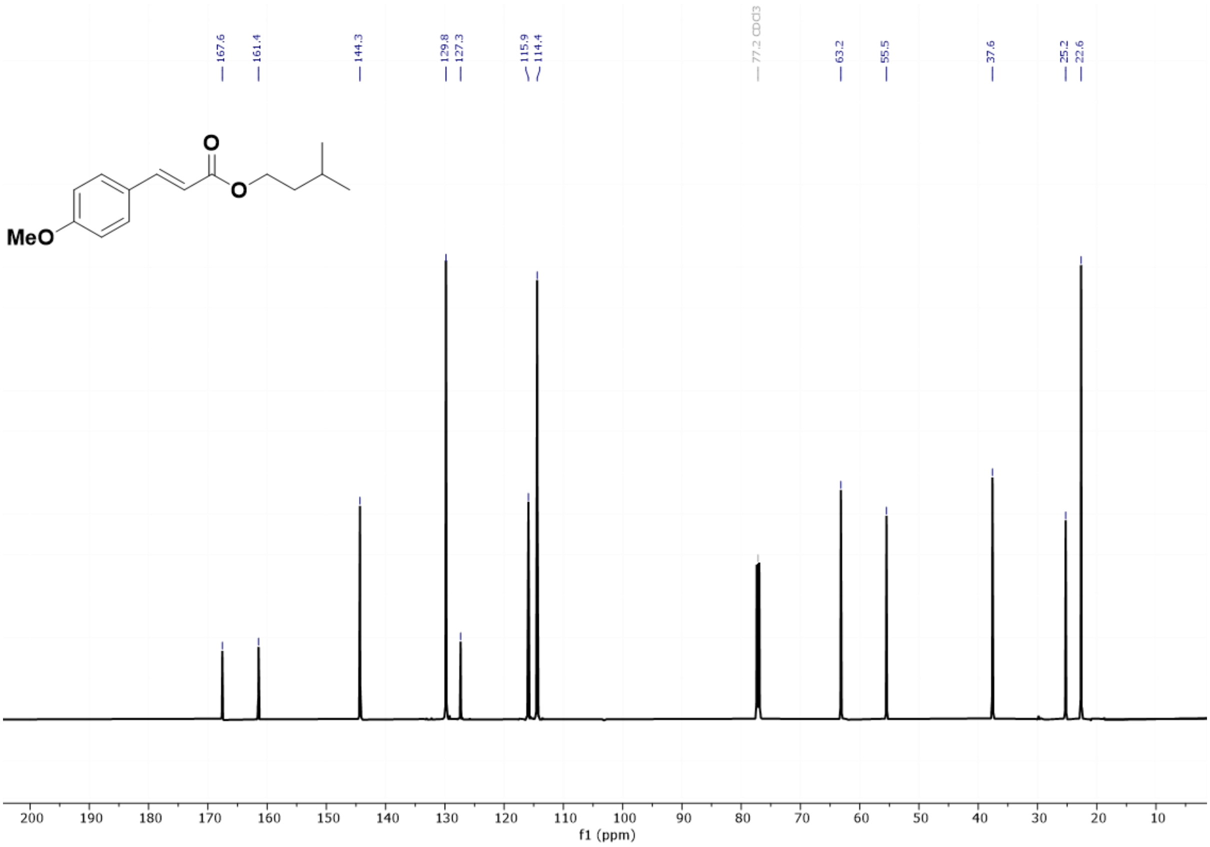

^{1}H-NMR (600 MHz, $CDCl_3$) of **3ga**

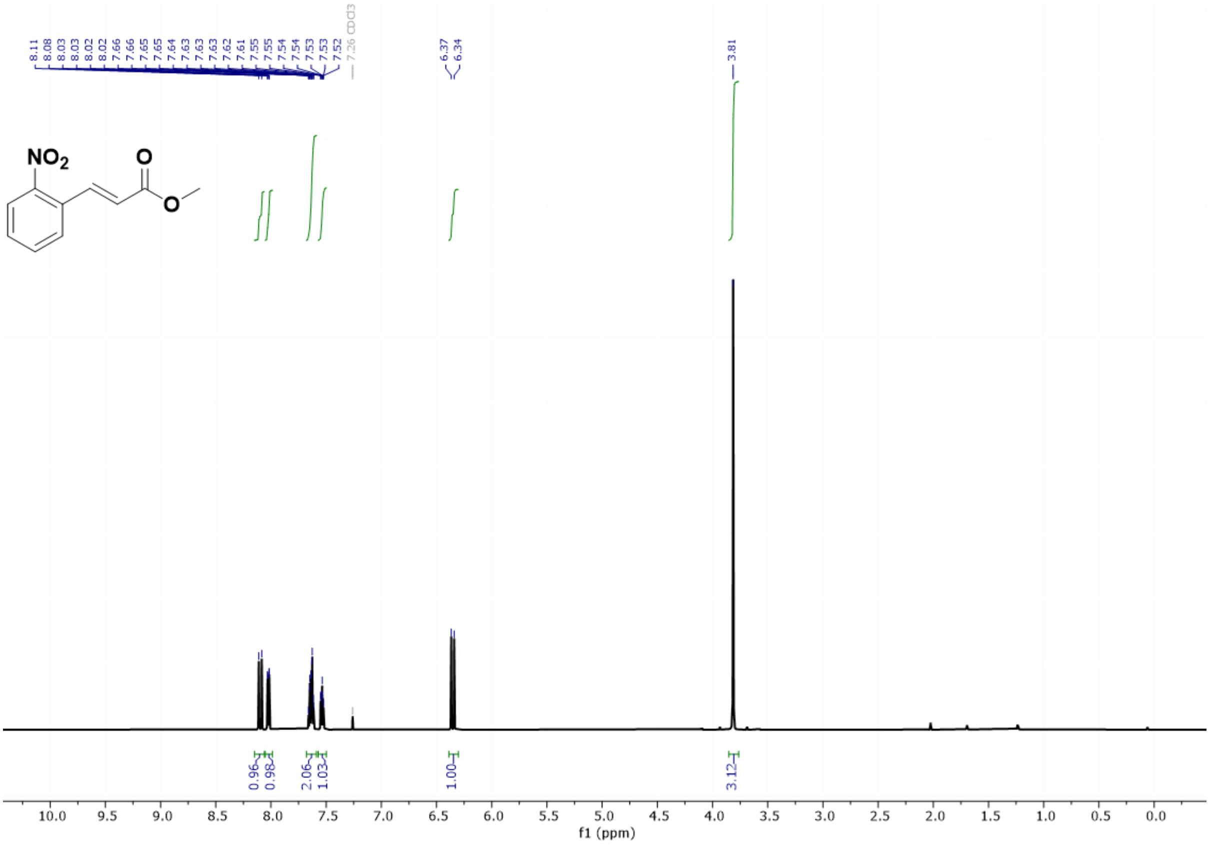

^{13}C-NMR (151 MHz, $CDCl_3$) of **3ga**

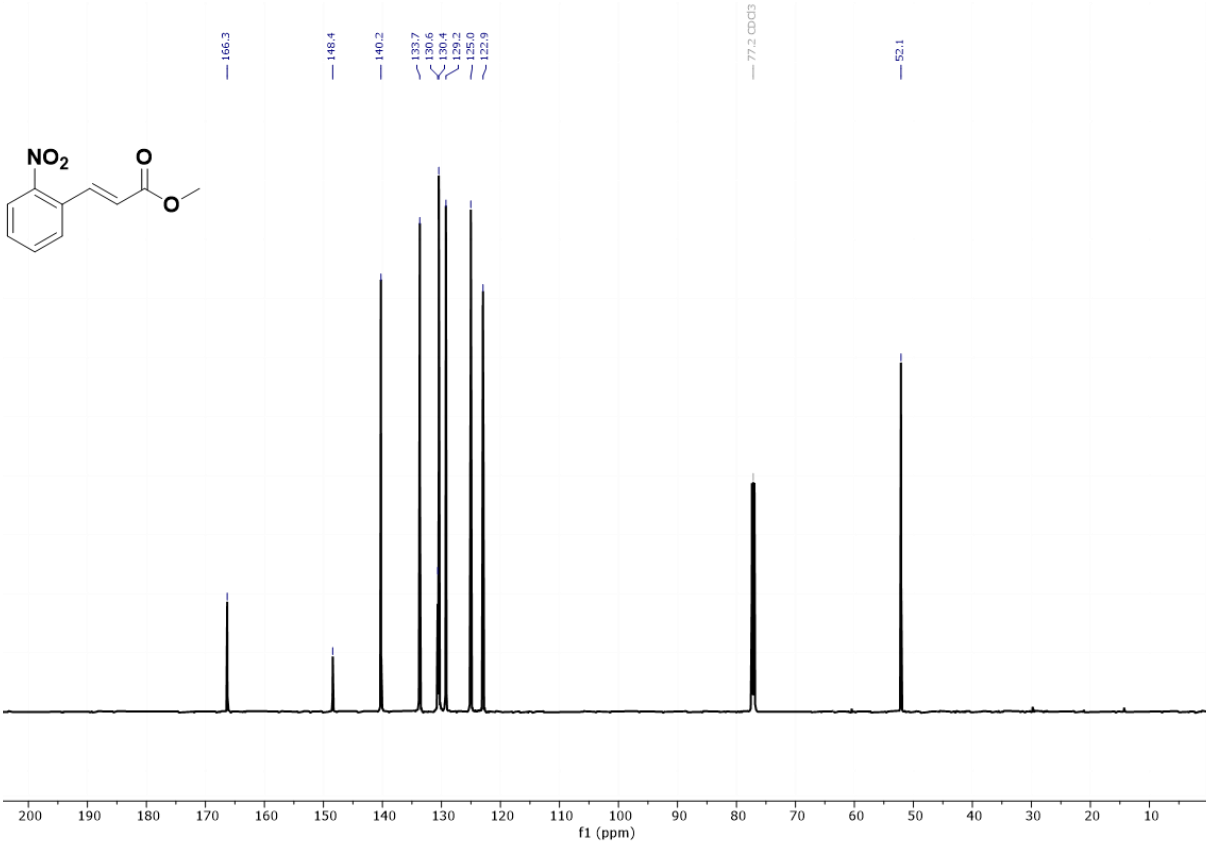

^{1}H-NMR (600 MHz, $CDCl_3$) of **3ac**

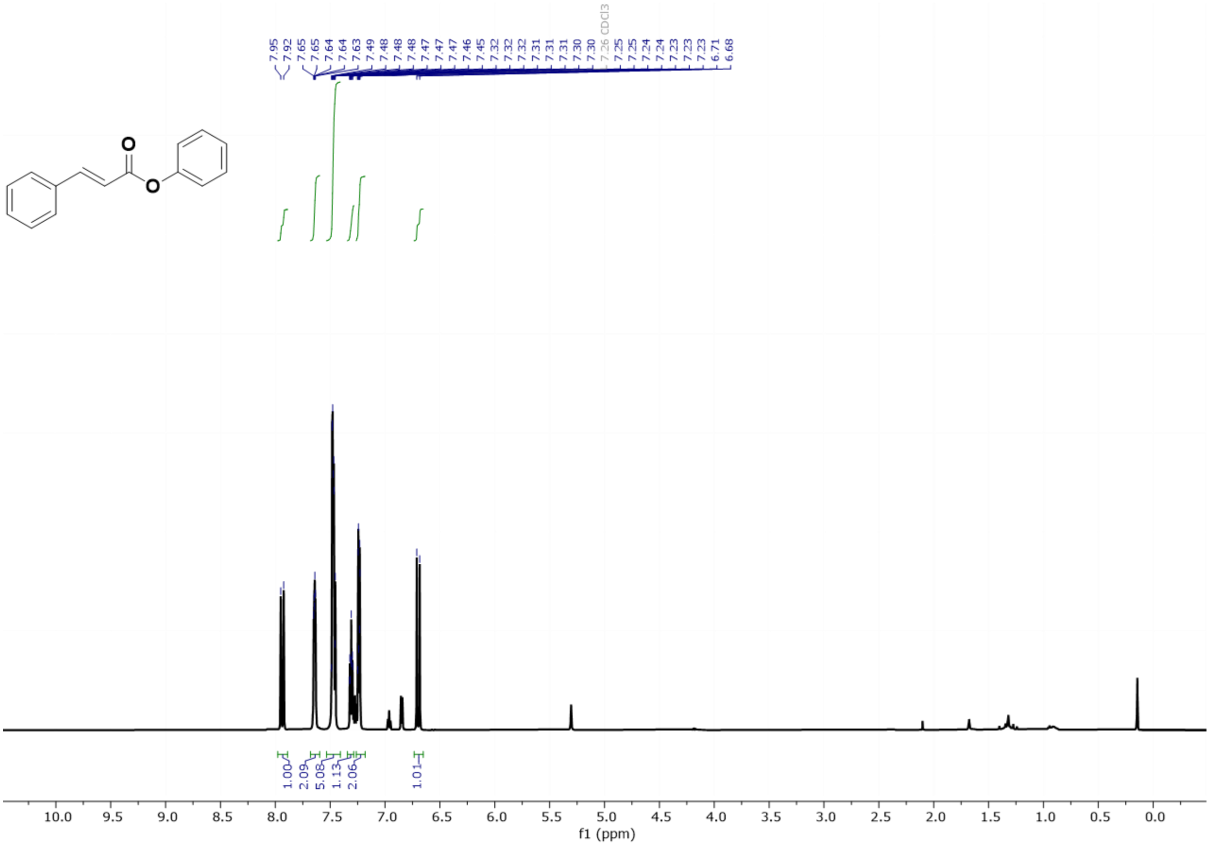

^{13}C-NMR (151 MHz, $CDCl_3$) of **3ac**

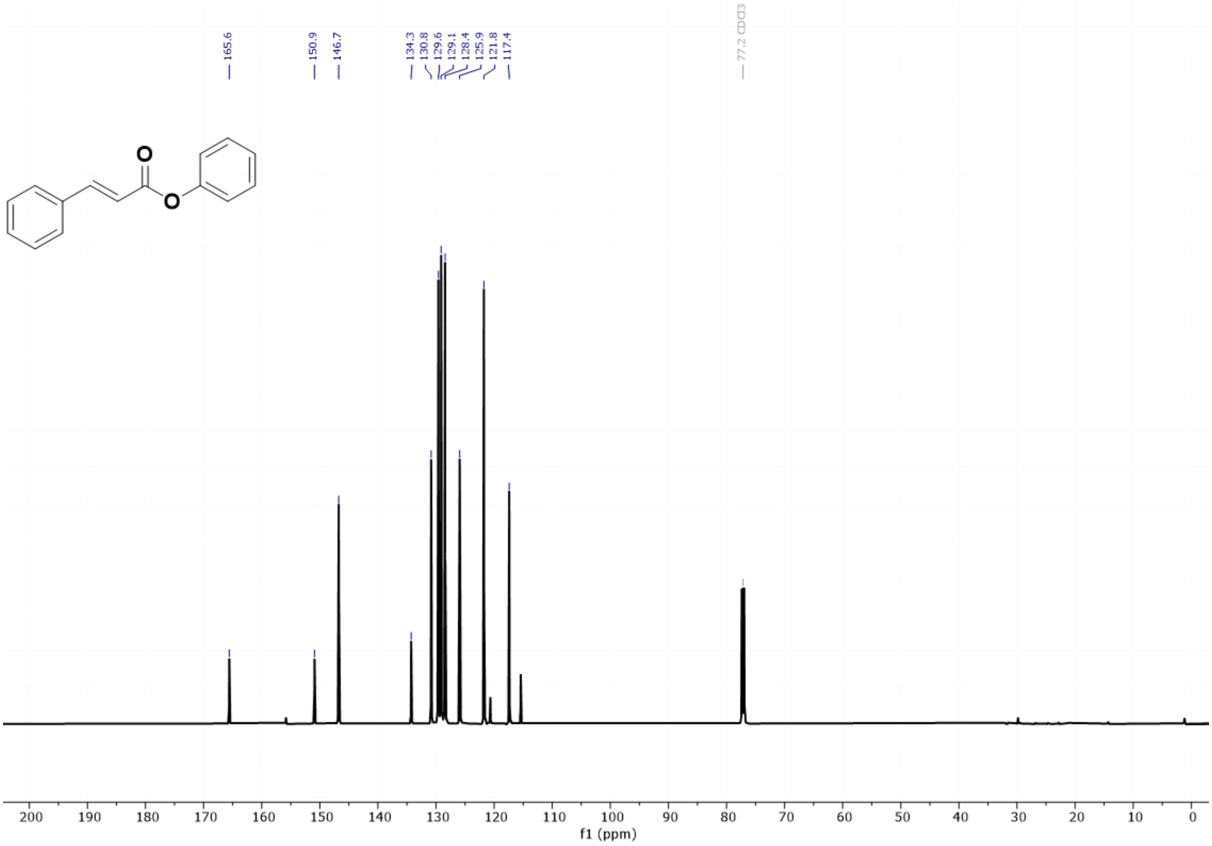

^{1}H-NMR (600 MHz, $CDCl_3$) of **3ad**

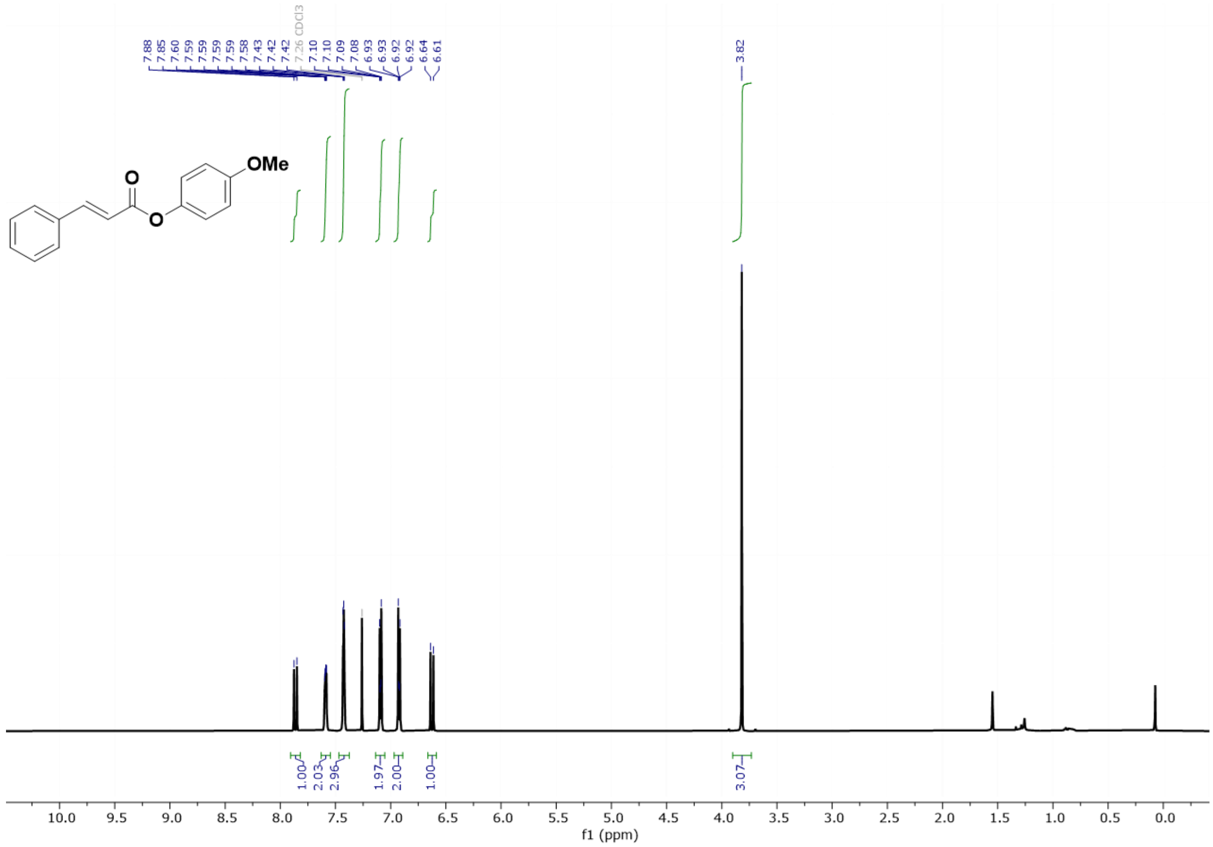

^{13}C-NMR (151 MHz, $CDCl_3$) of **3ad**

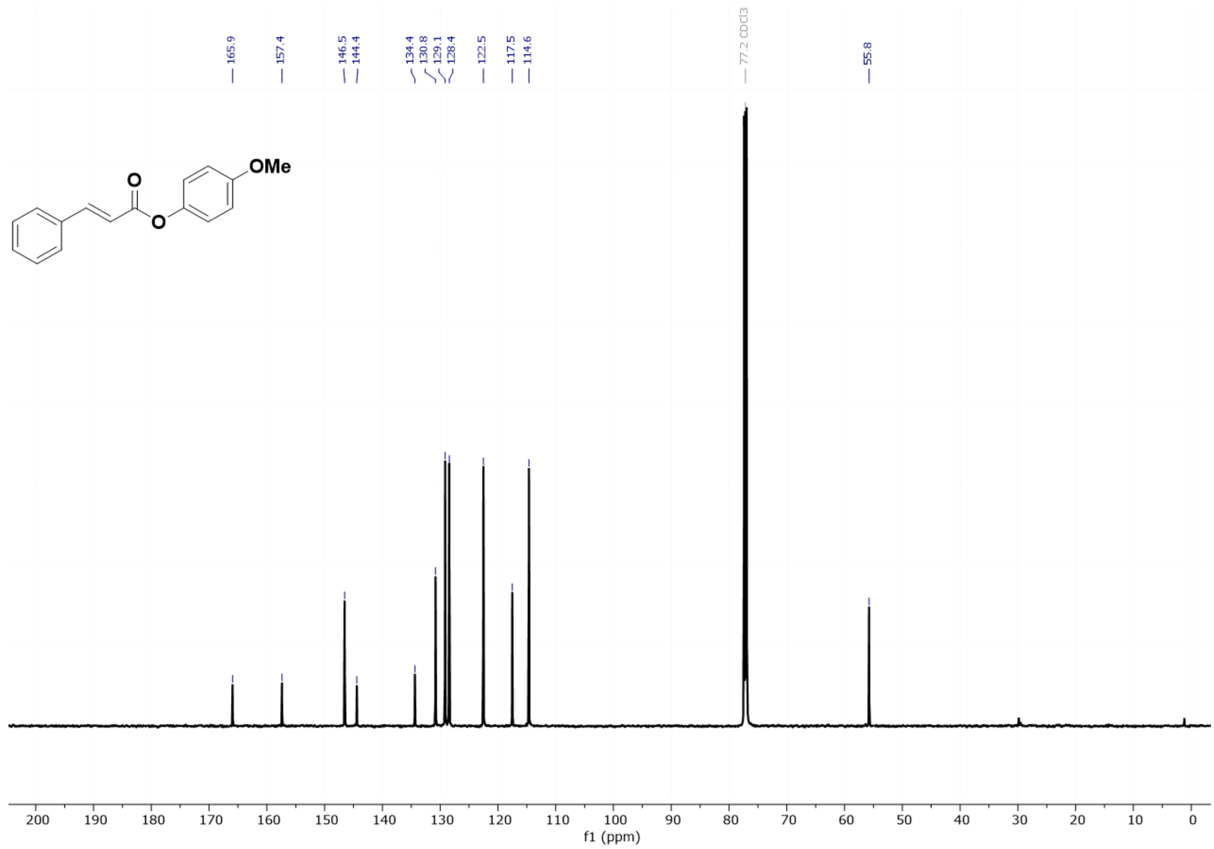

^{1}H-NMR (600 MHz, $CDCl_3$) of **3ae**

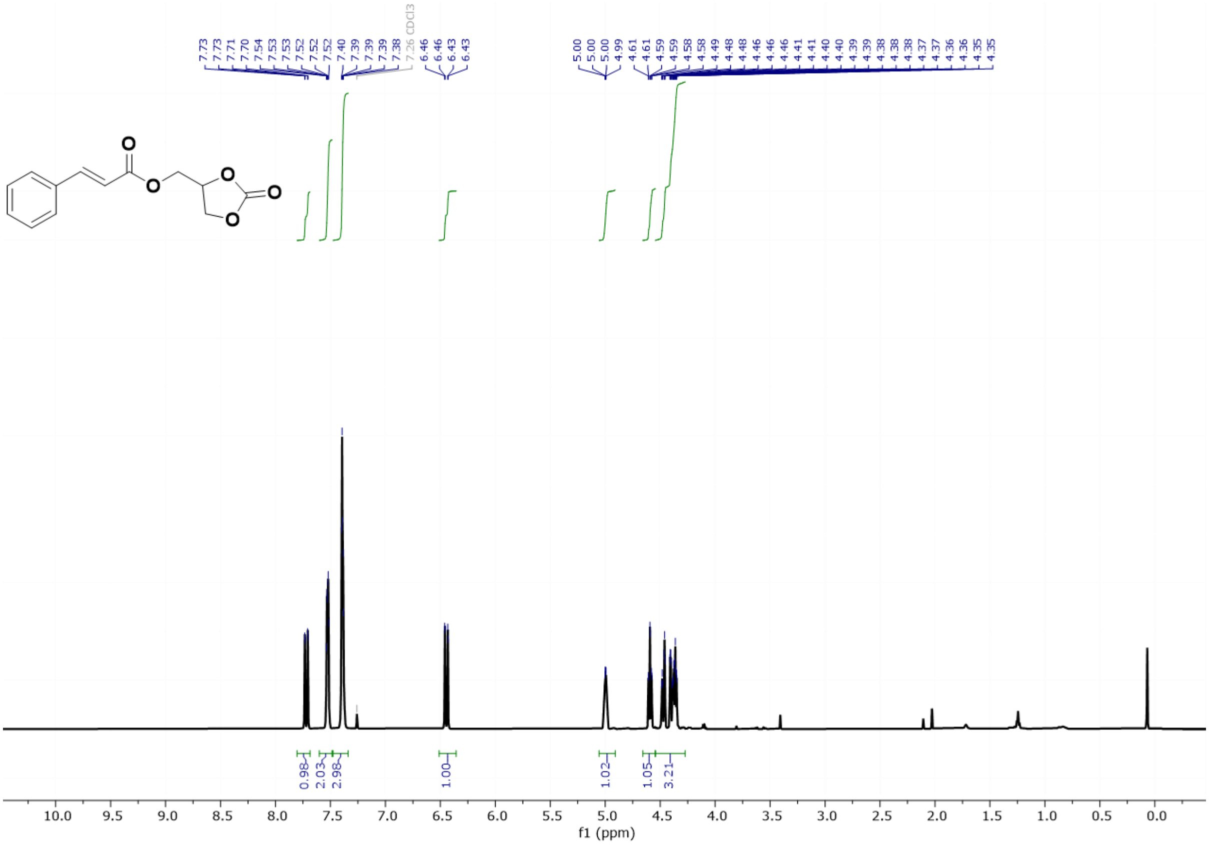

^{13}C-NMR (151 MHz, $CDCl_3$) of **3ae**

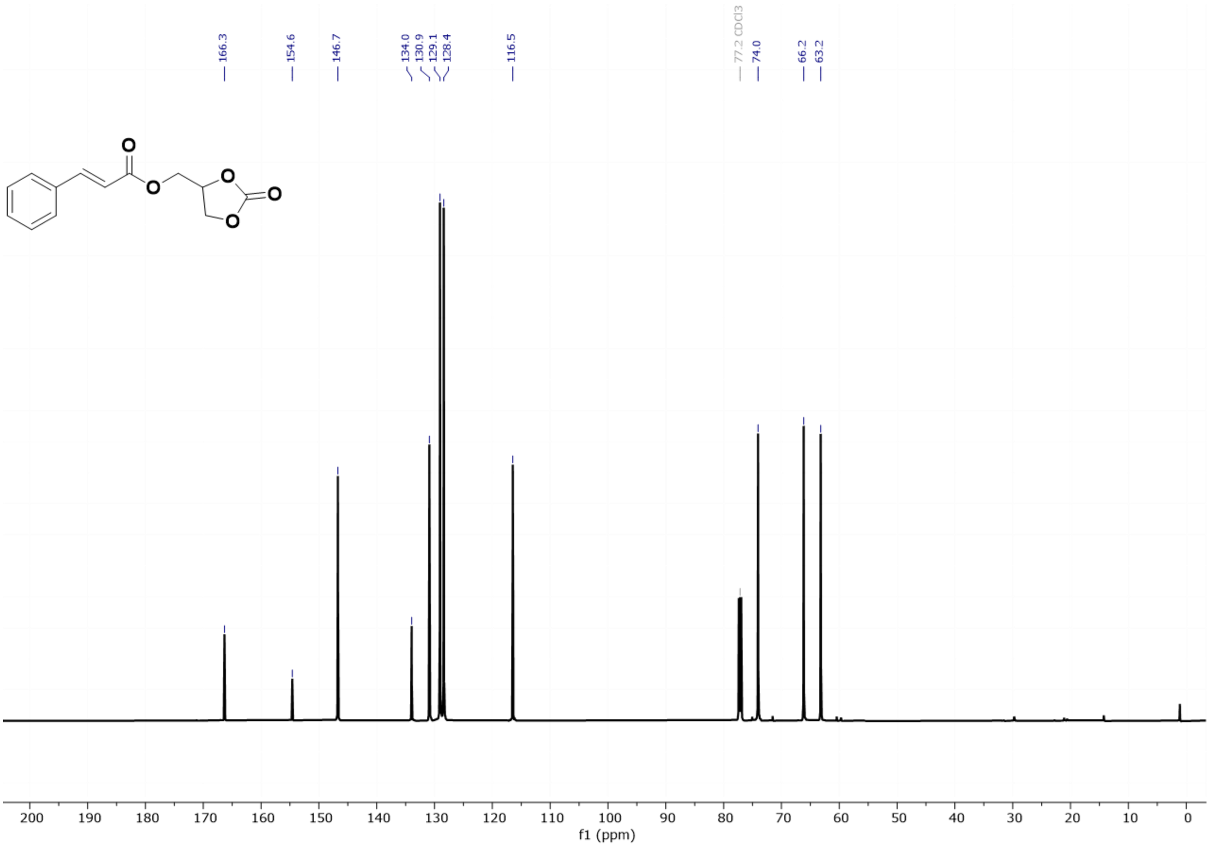

^{1}H-NMR (600 MHz, $CDCl_3$) of **3af**

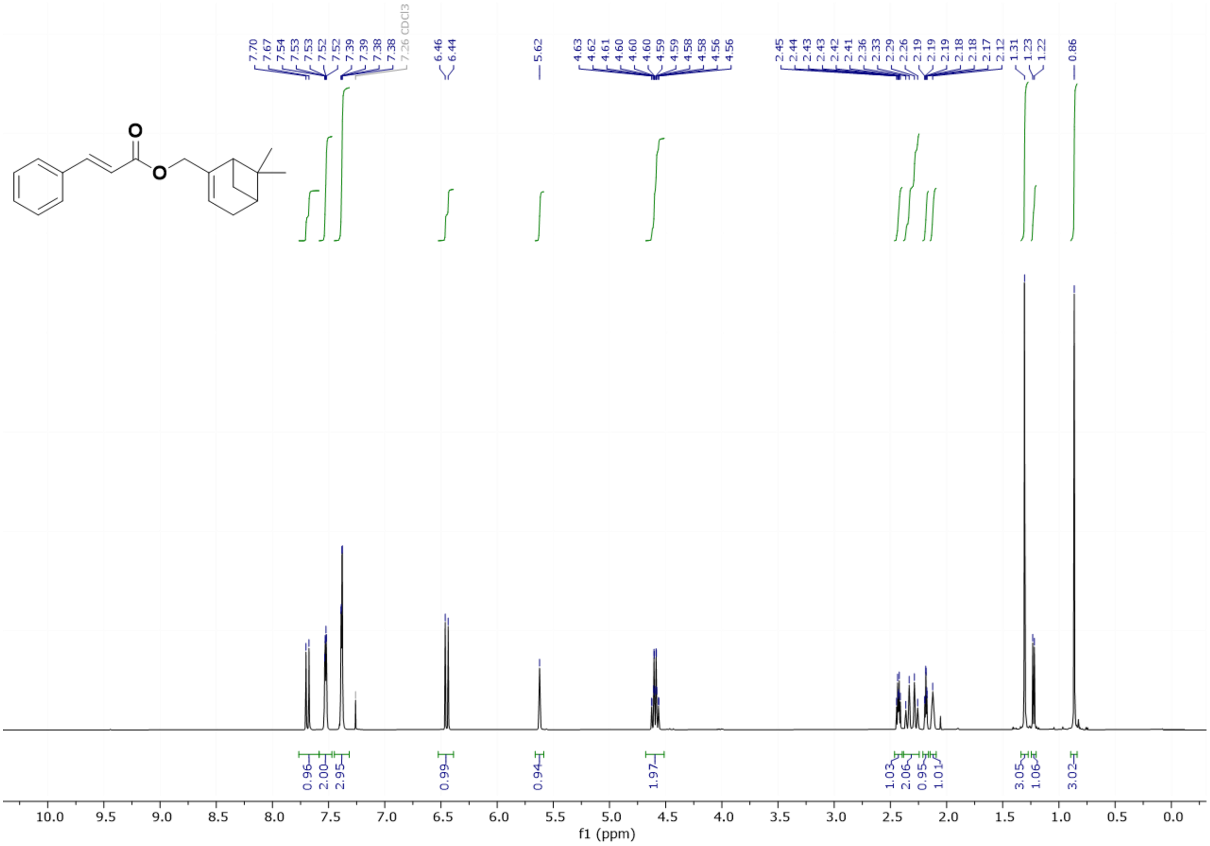

^{13}C-NMR (151 MHz, $CDCl_3$) of **3af**

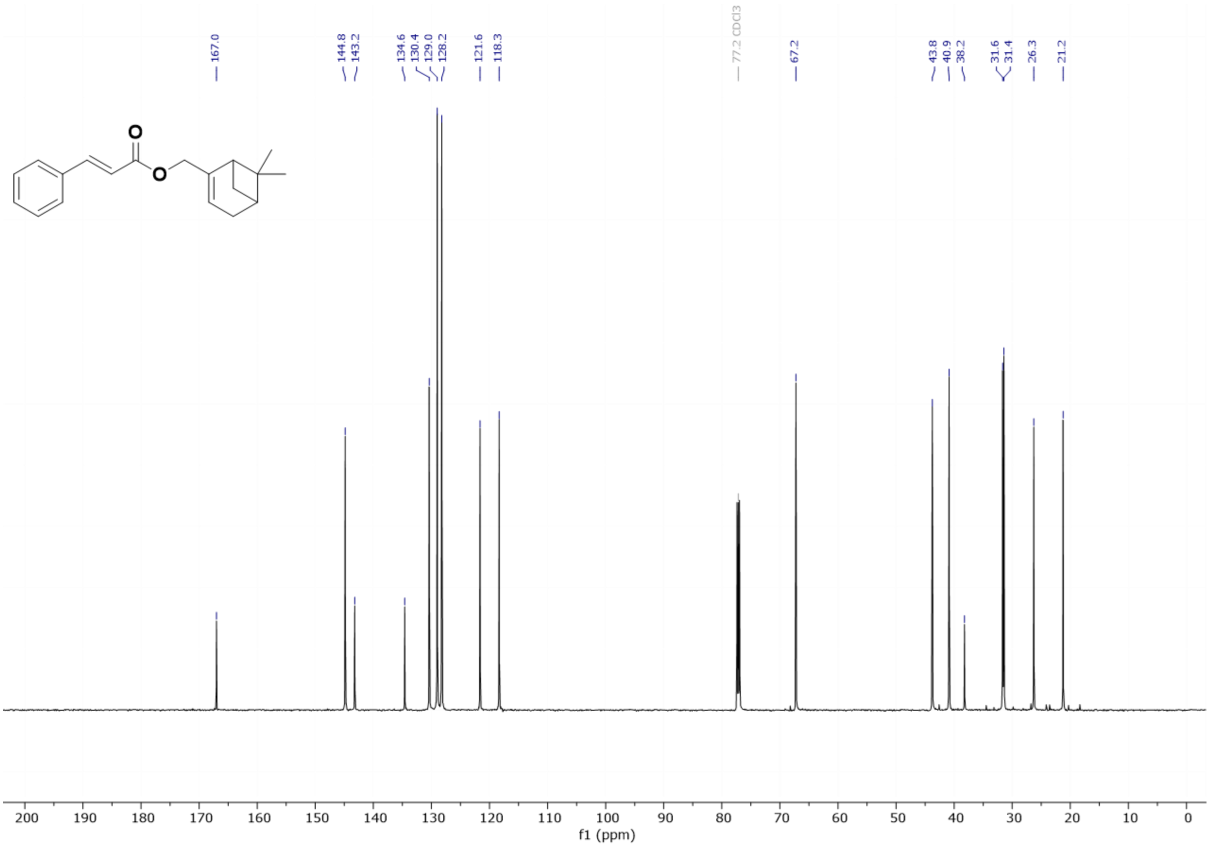

^{1}H-NMR (600 MHz, $CDCl_3$) of **3ha**

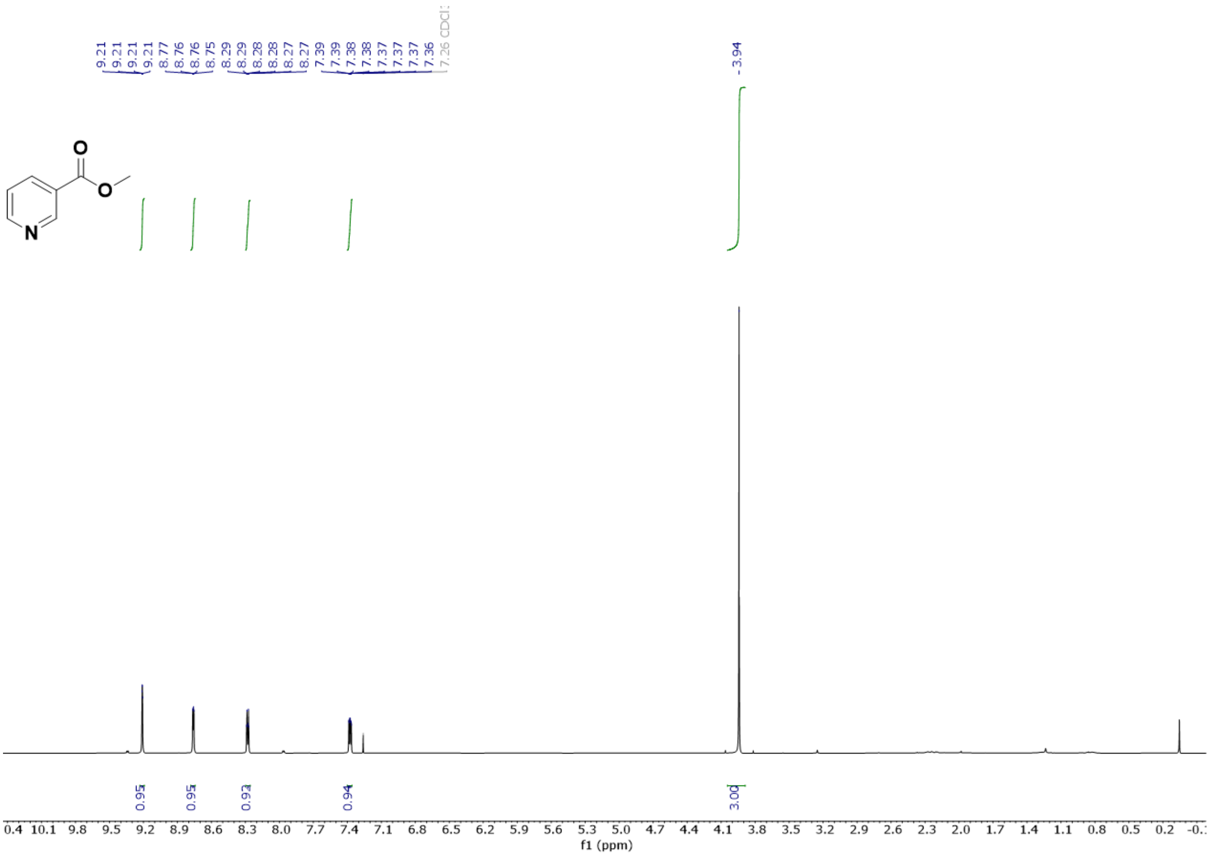

^{13}C-NMR (151 MHz, $CDCl_3$) of **3ha**

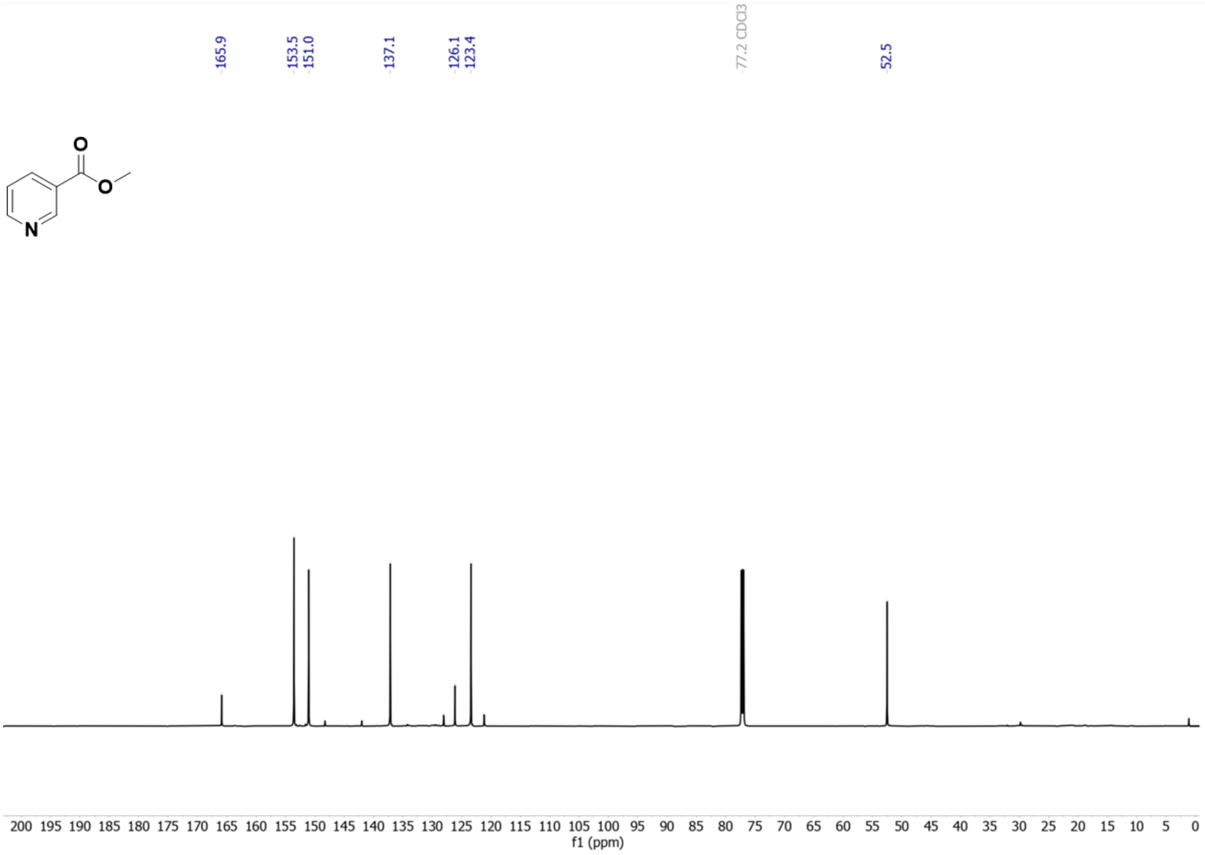

^{1}H-NMR (700 MHz, $CDCl_3$) of **3ia**

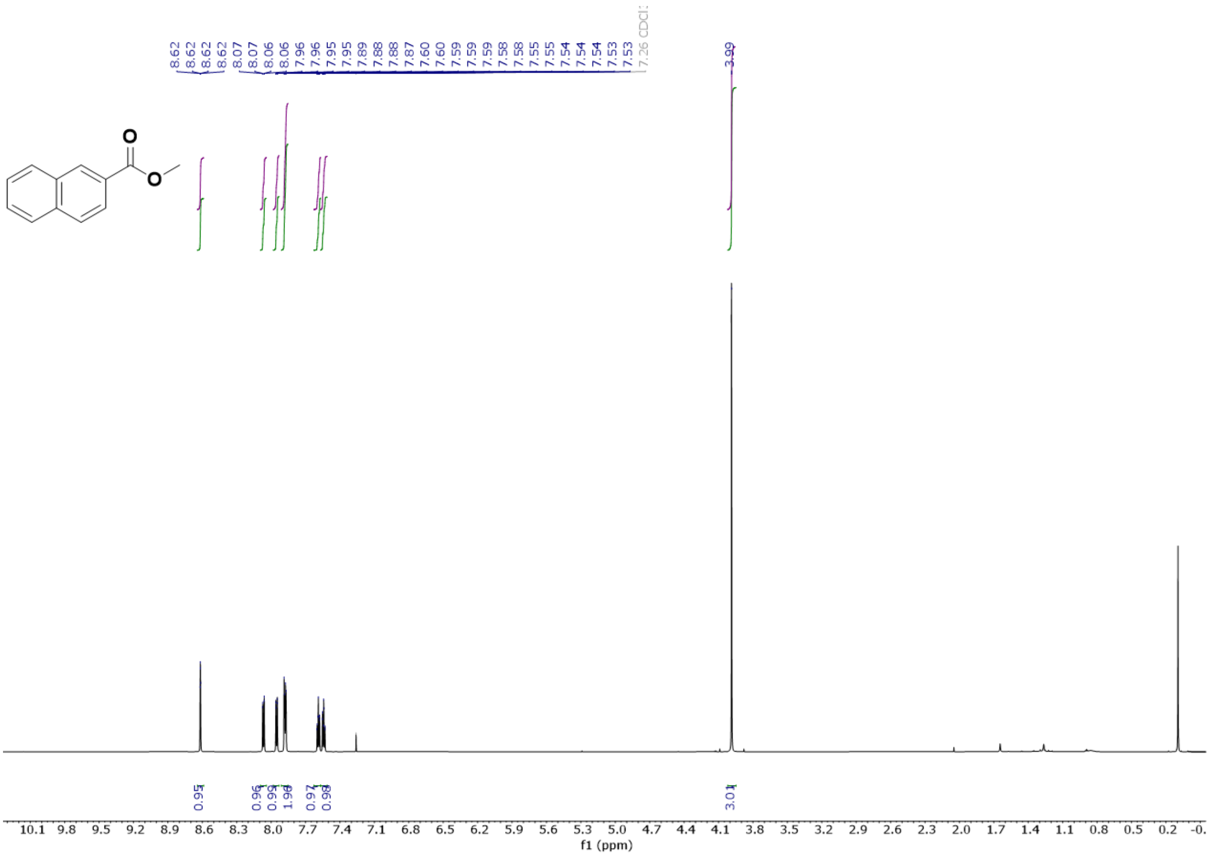

^{13}C-NMR (176 MHz, $CDCl_3$) of **3ia**

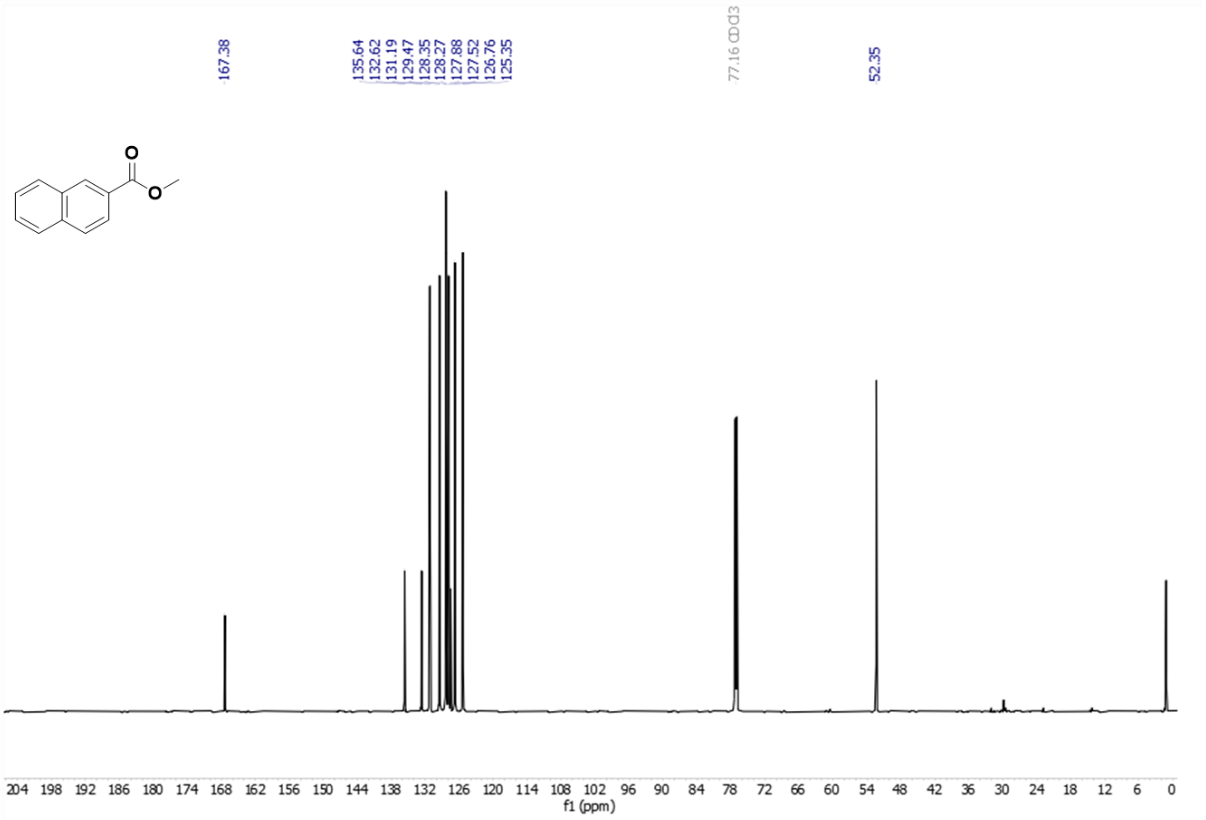

^{1}H-NMR (600 MHz, $CDCl_3$) of **3ja**

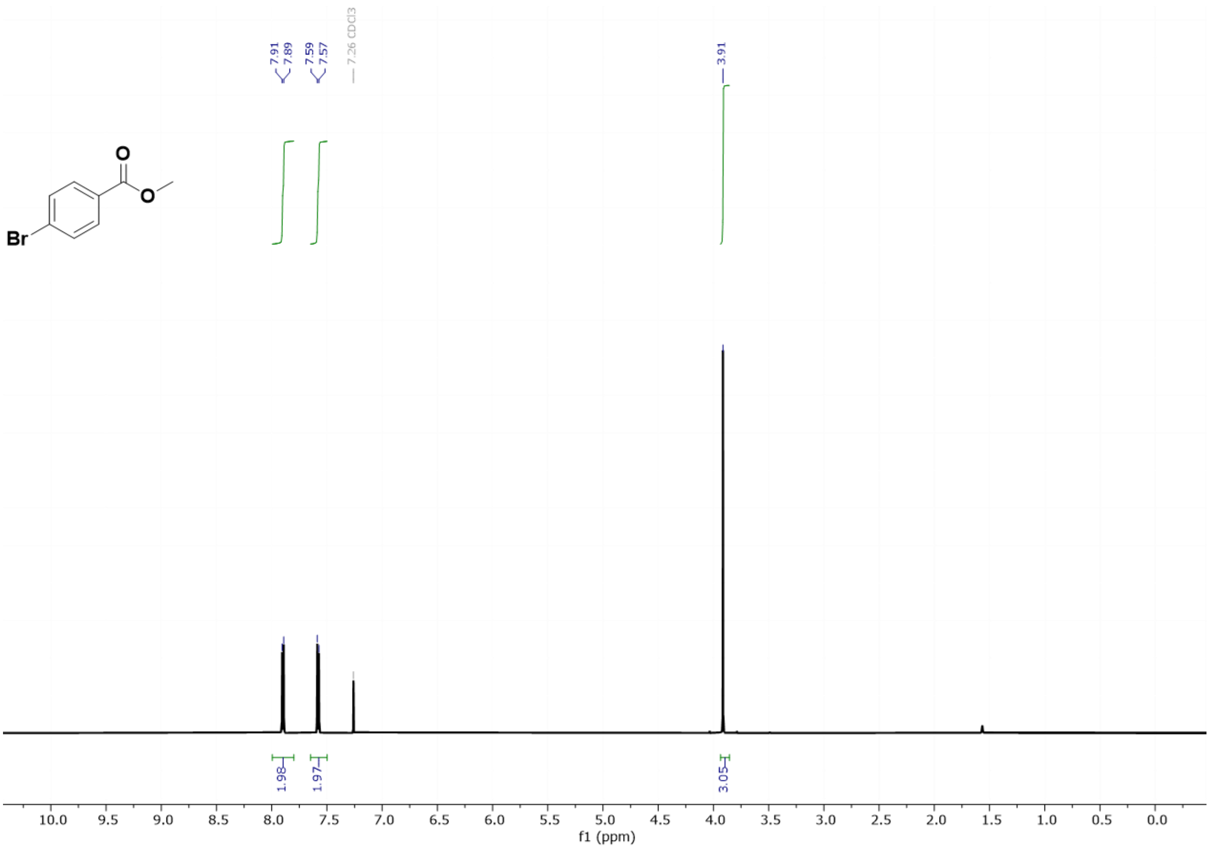

^{13}C-NMR (151 MHz, $CDCl_3$) of **3ja**

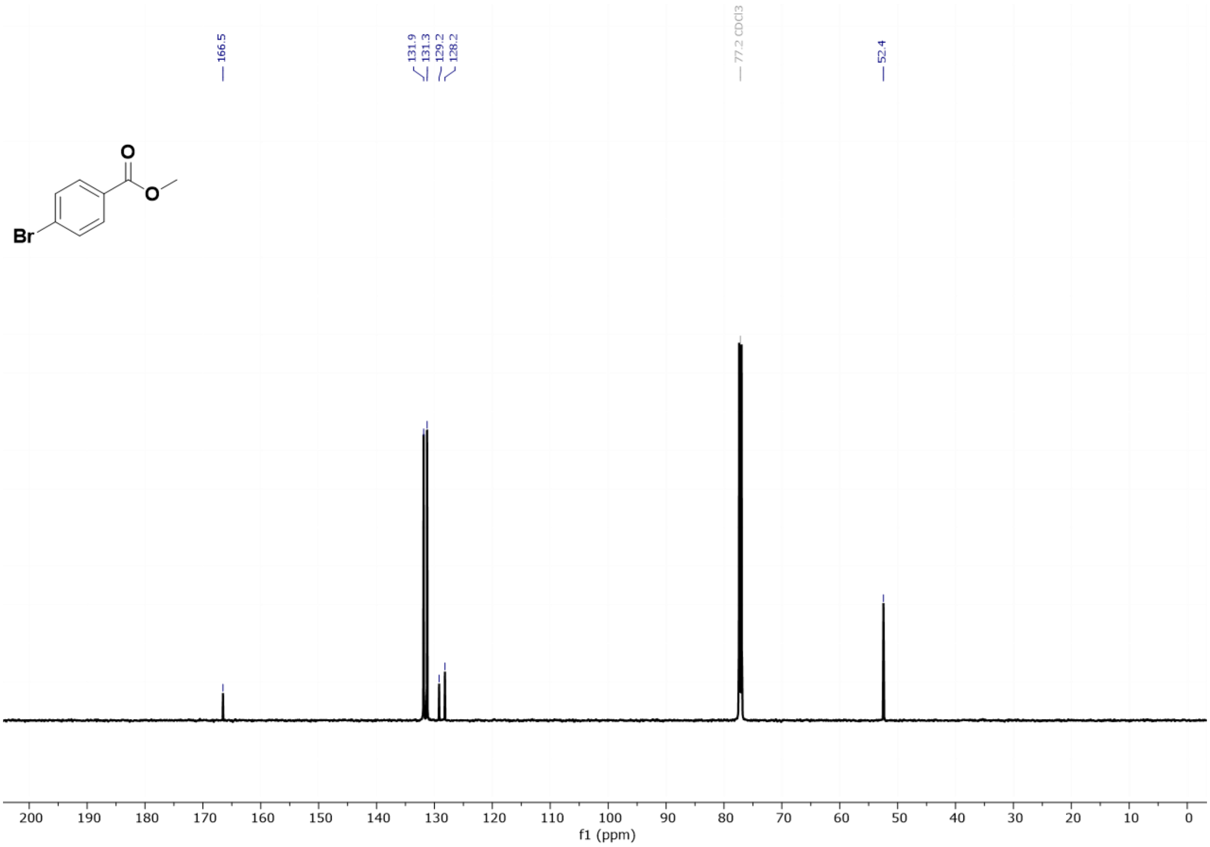

^{1}H-NMR (600 MHz, $CDCl_3$) of **3ka**

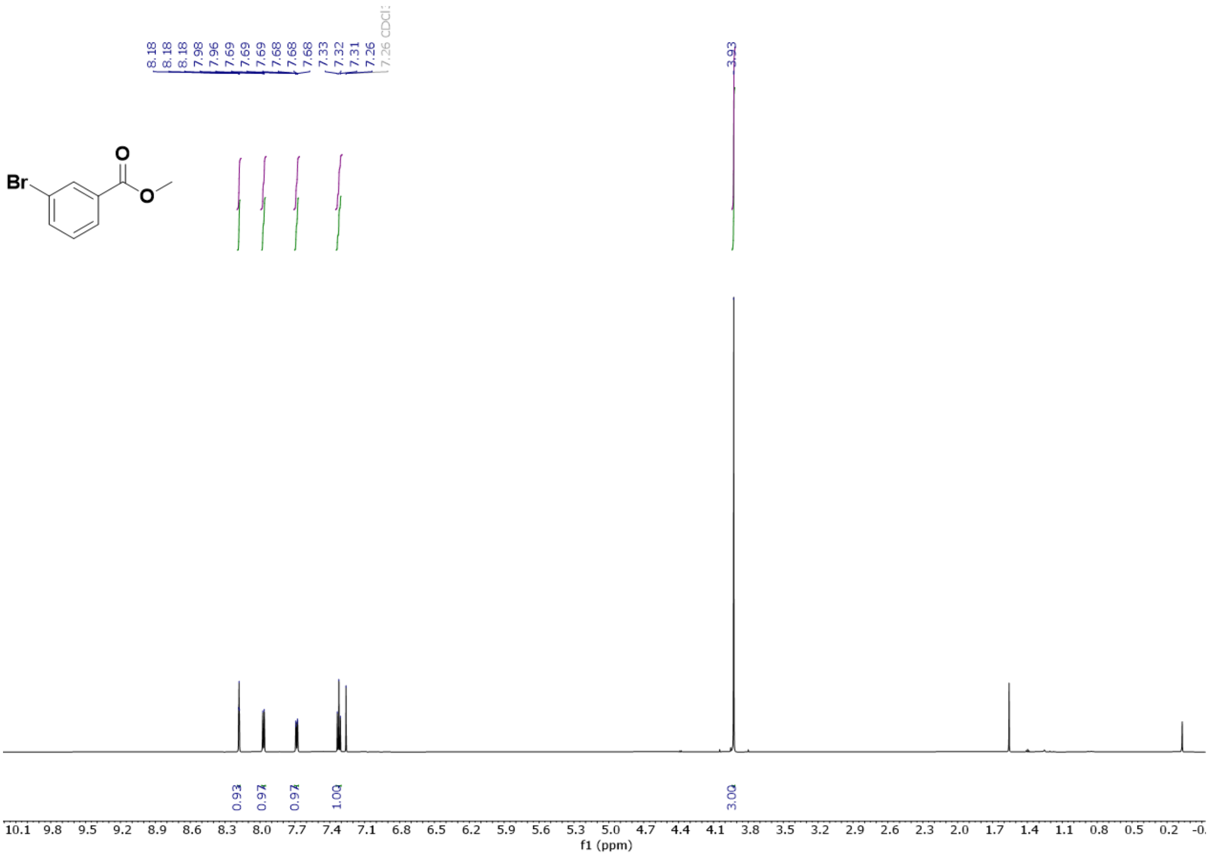

^{13}C-NMR (151 MHz, $CDCl_3$) of **3ka**

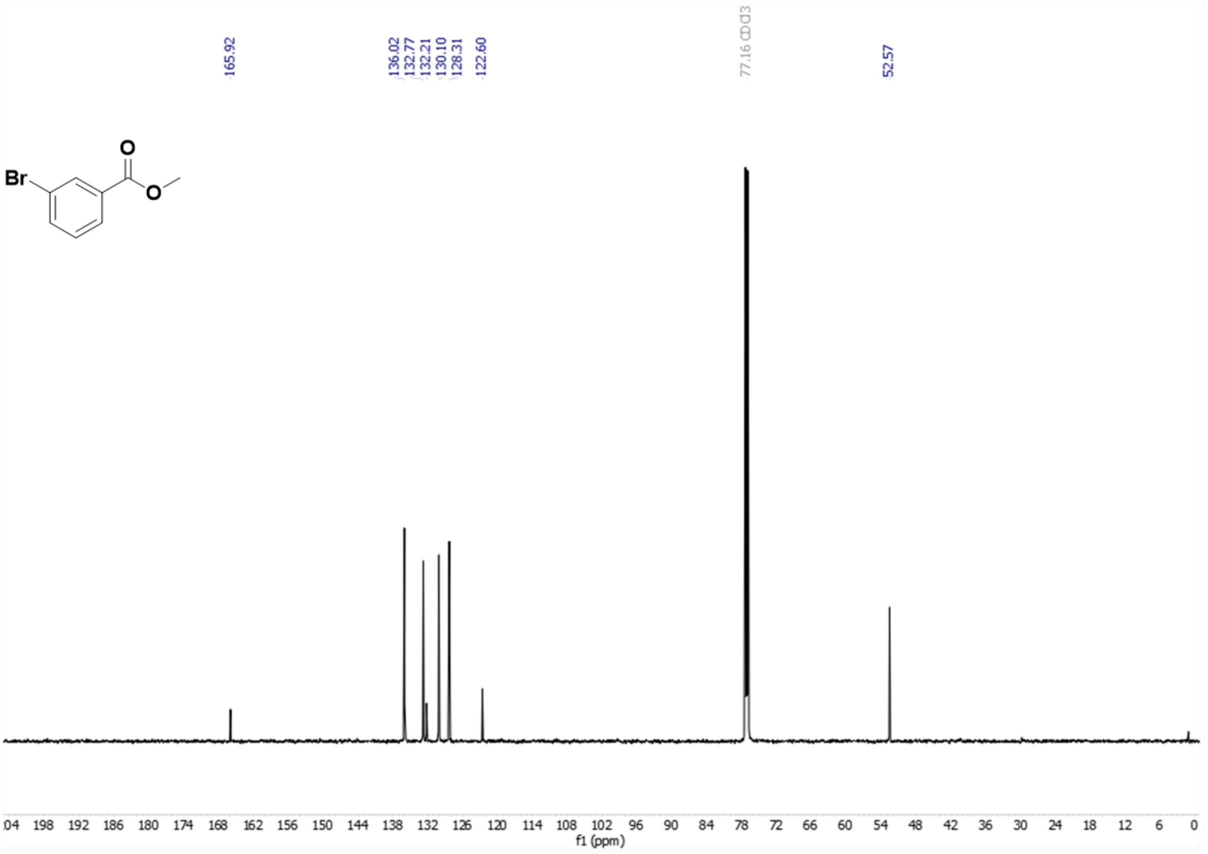

^{1}H-NMR (600 MHz, $CDCl_3$) of **3la**

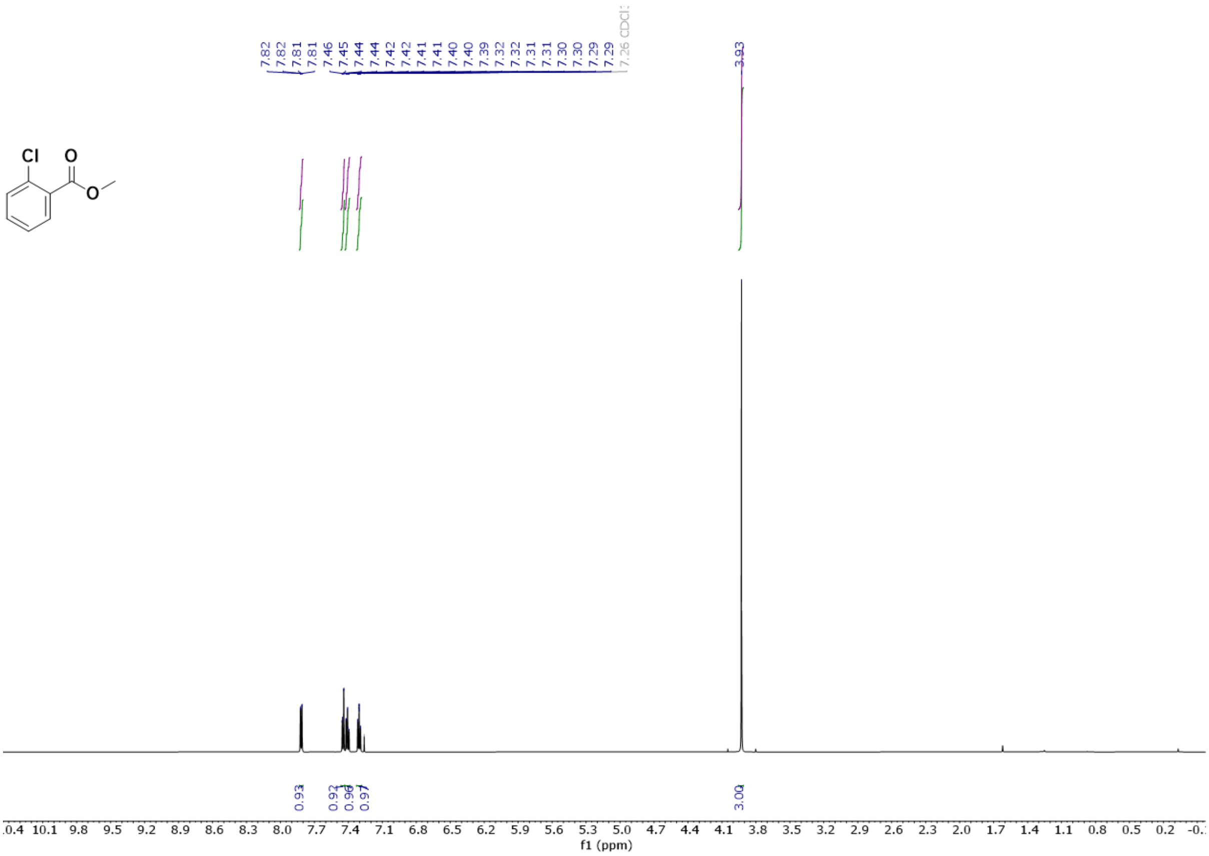

^{13}C-NMR (151 MHz, $CDCl_3$) of **3la**

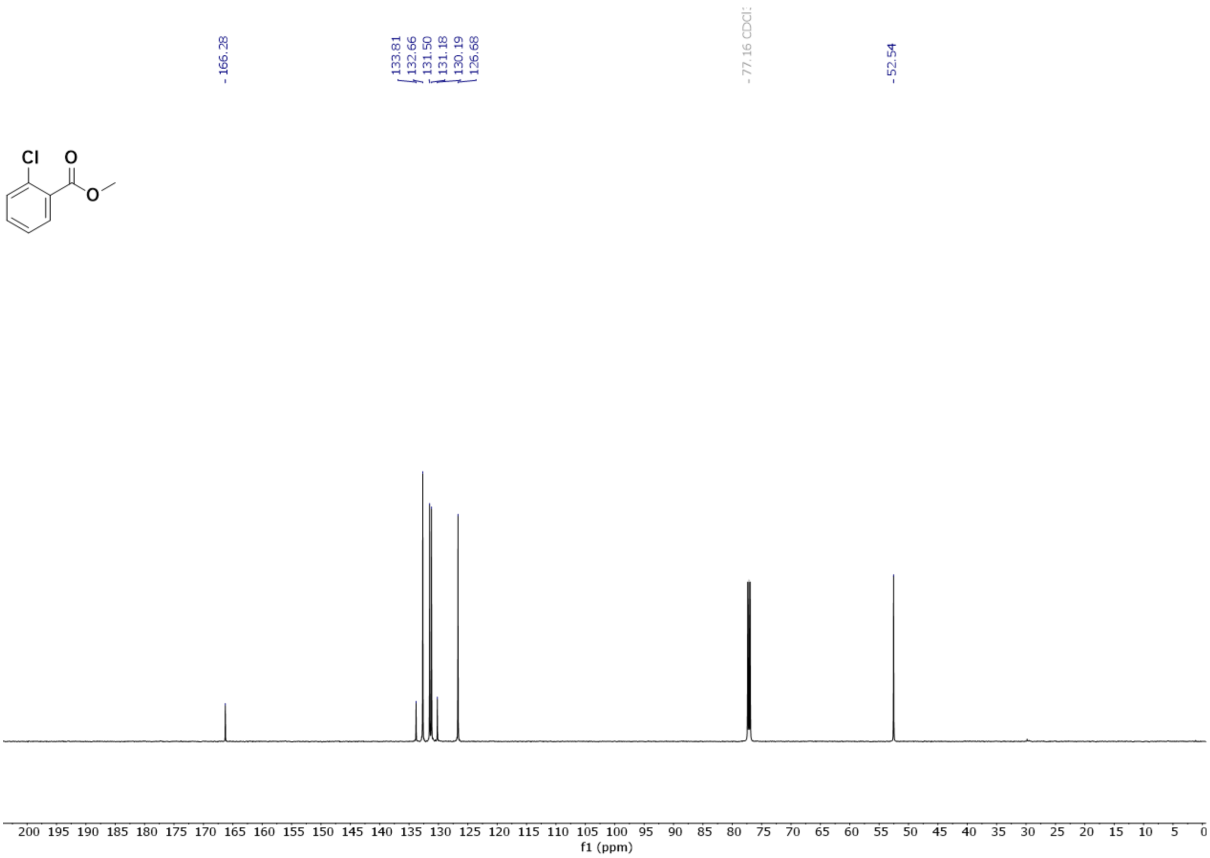

^{1}H-NMR (600 MHz, $CDCl_3$) of **3ma**

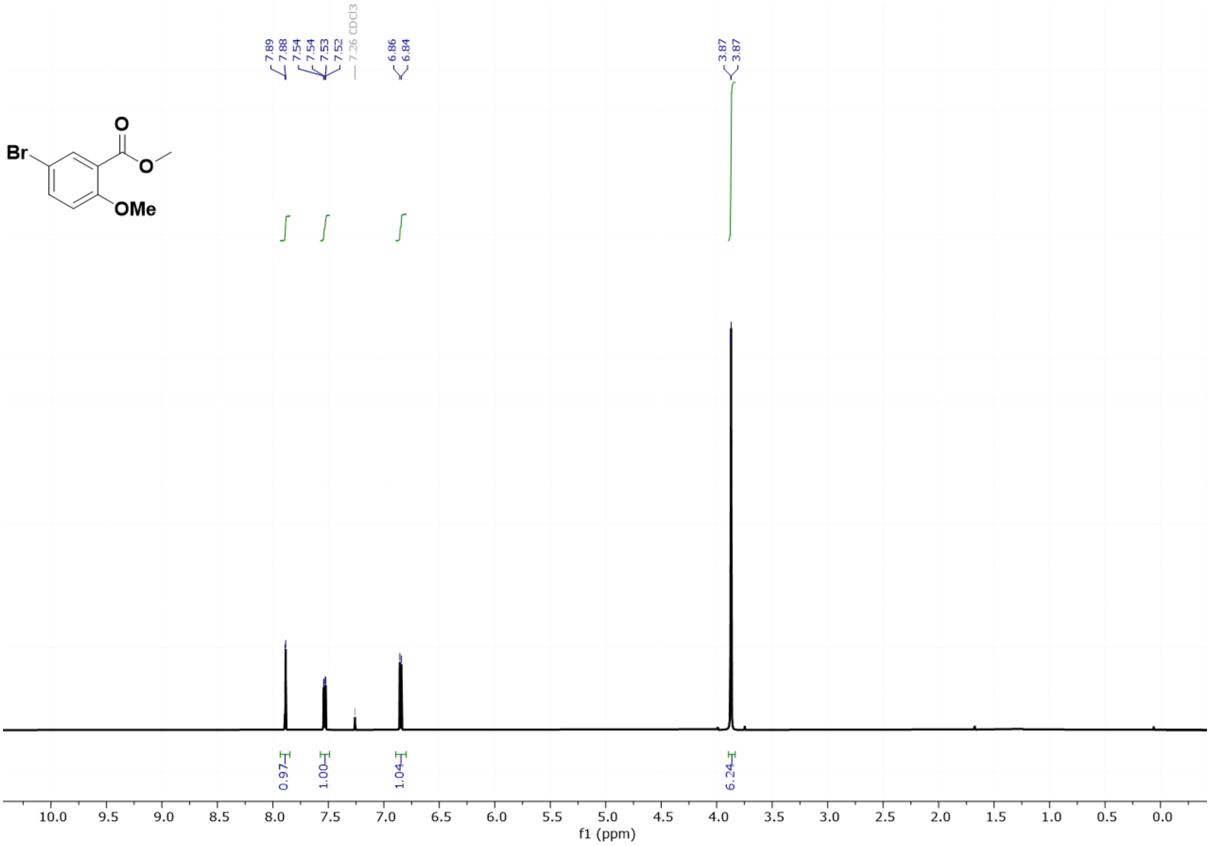

^{13}C-NMR (151 MHz, $CDCl_3$) of **3ma**

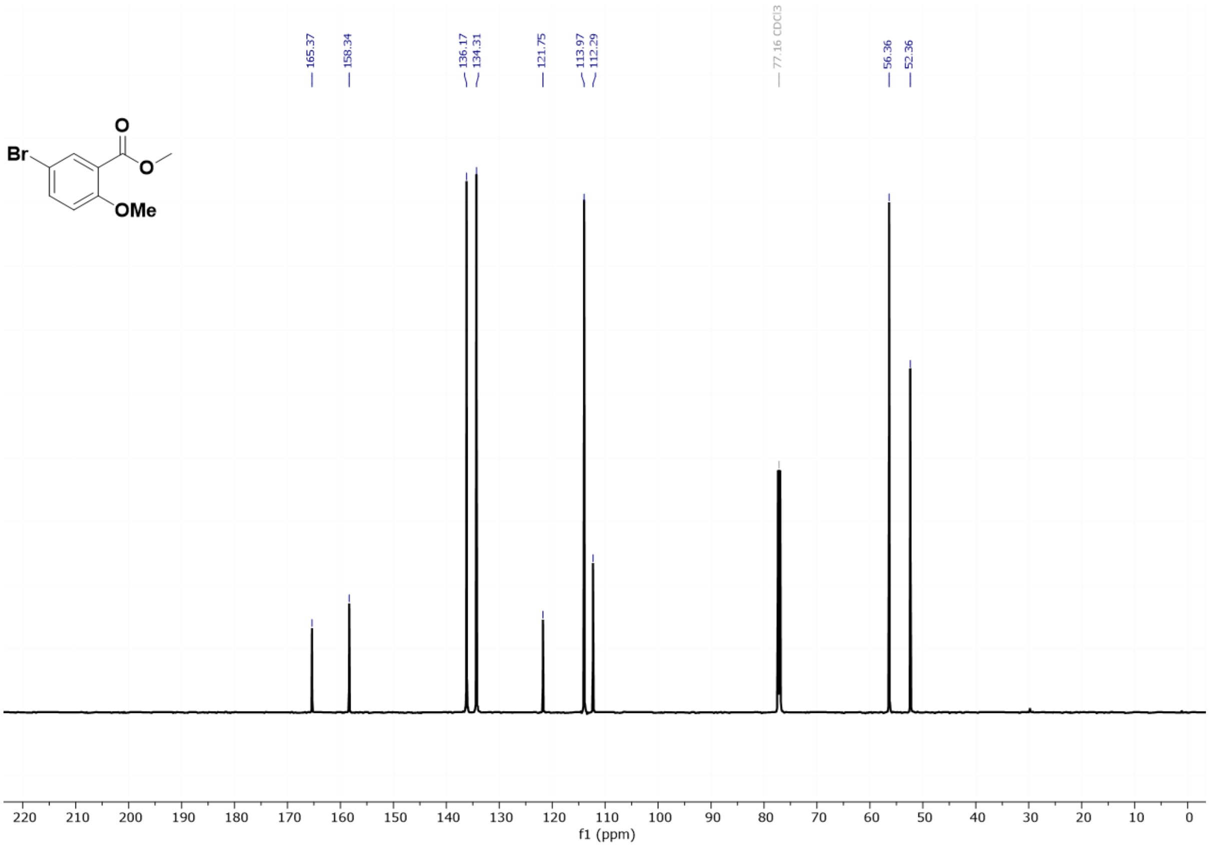

^{1}H-NMR (700 MHz, $CDCl_3$) of **3na**

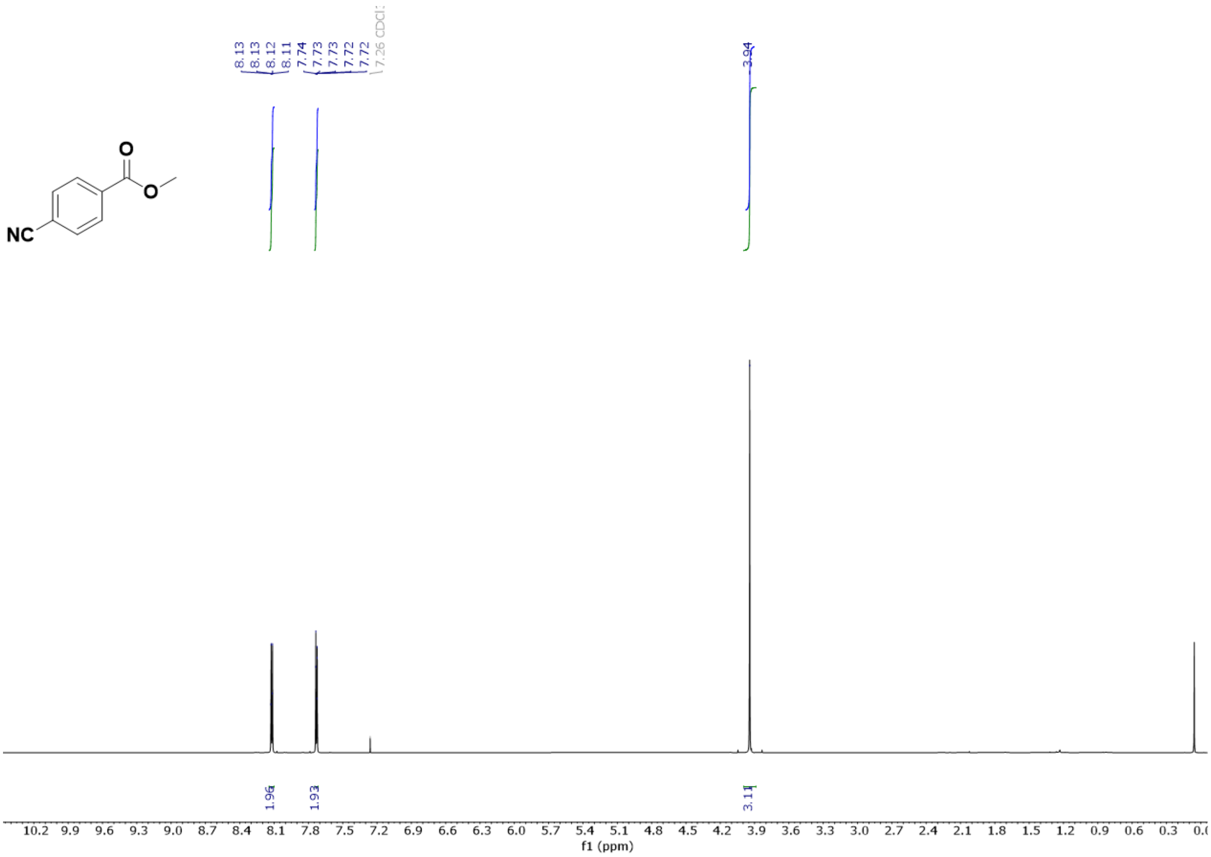

^{13}C-NMR (176 MHz, $CDCl_3$) of **3na**

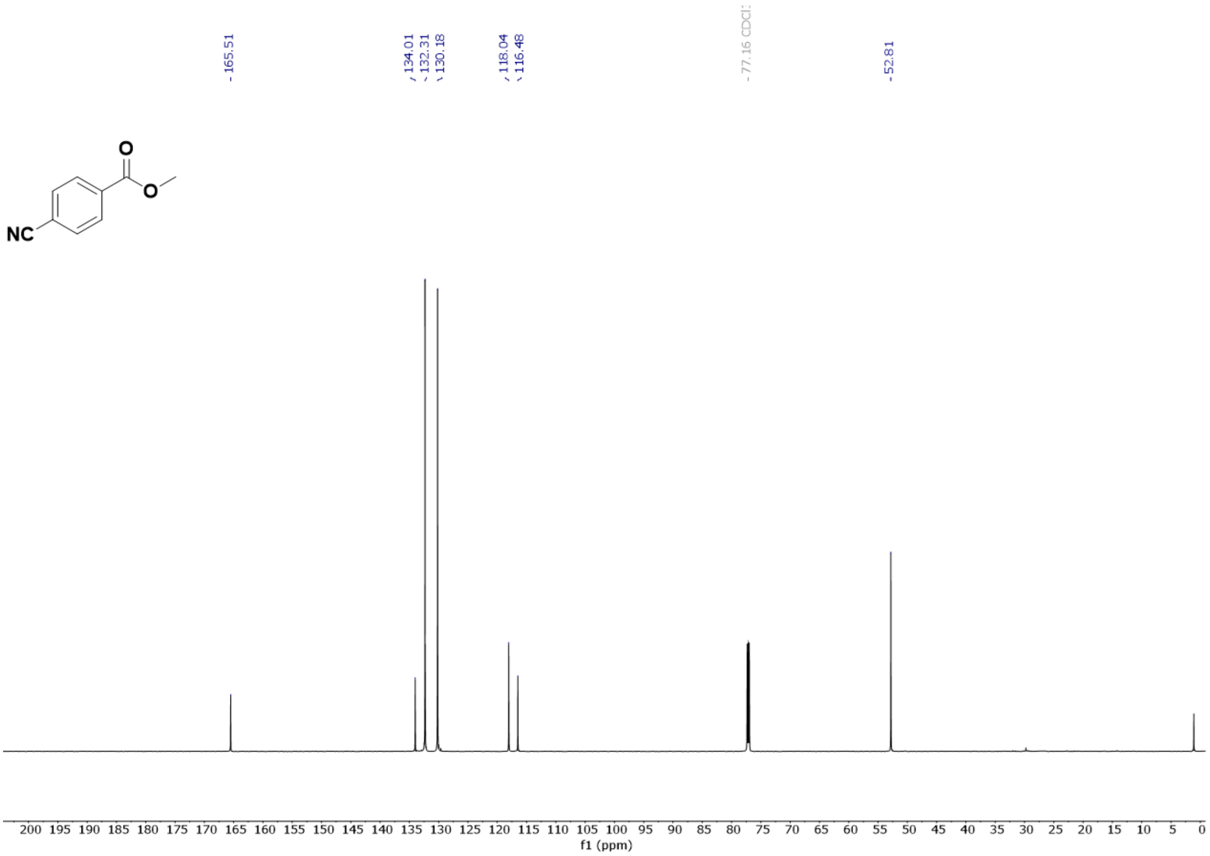

^{1}H-NMR (600 MHz, $CDCl_3$) of **9**

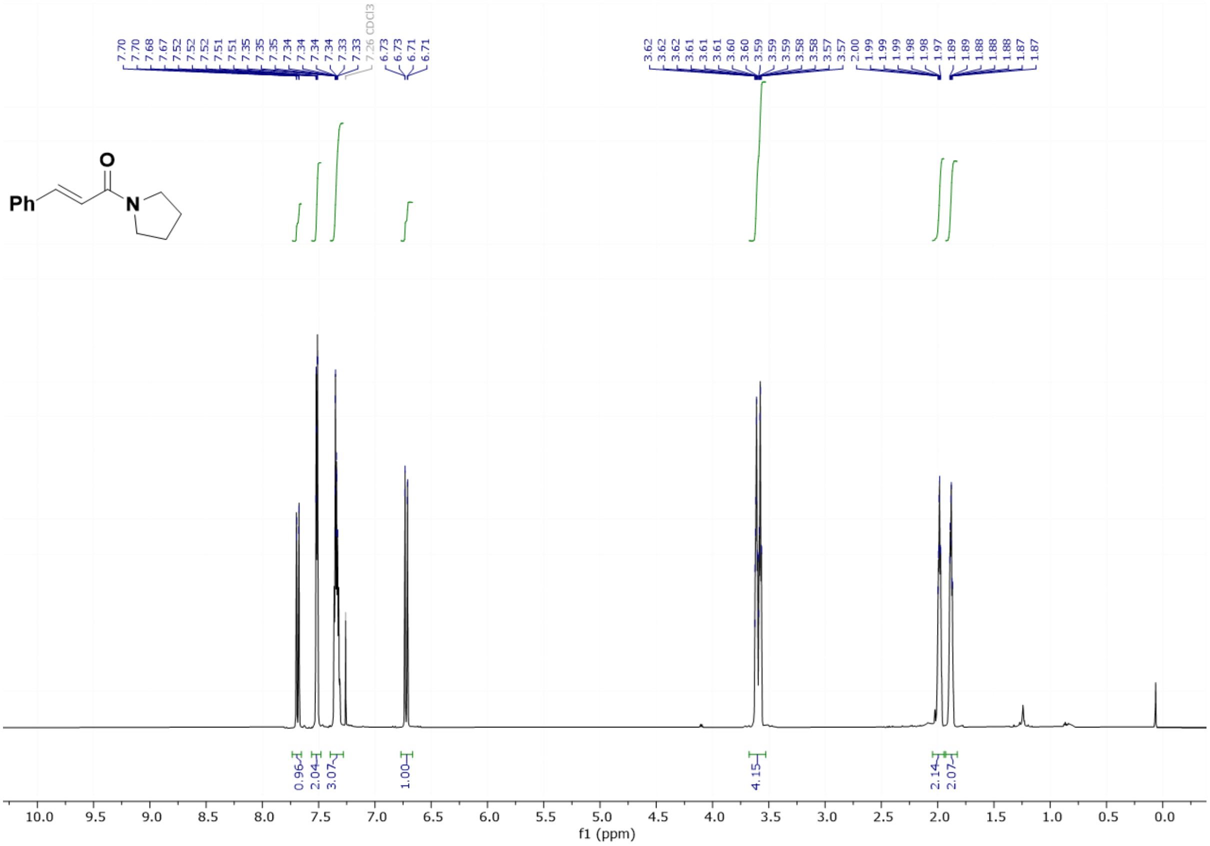

^{13}C-NMR (151 MHz, $CDCl_3$) of **9**

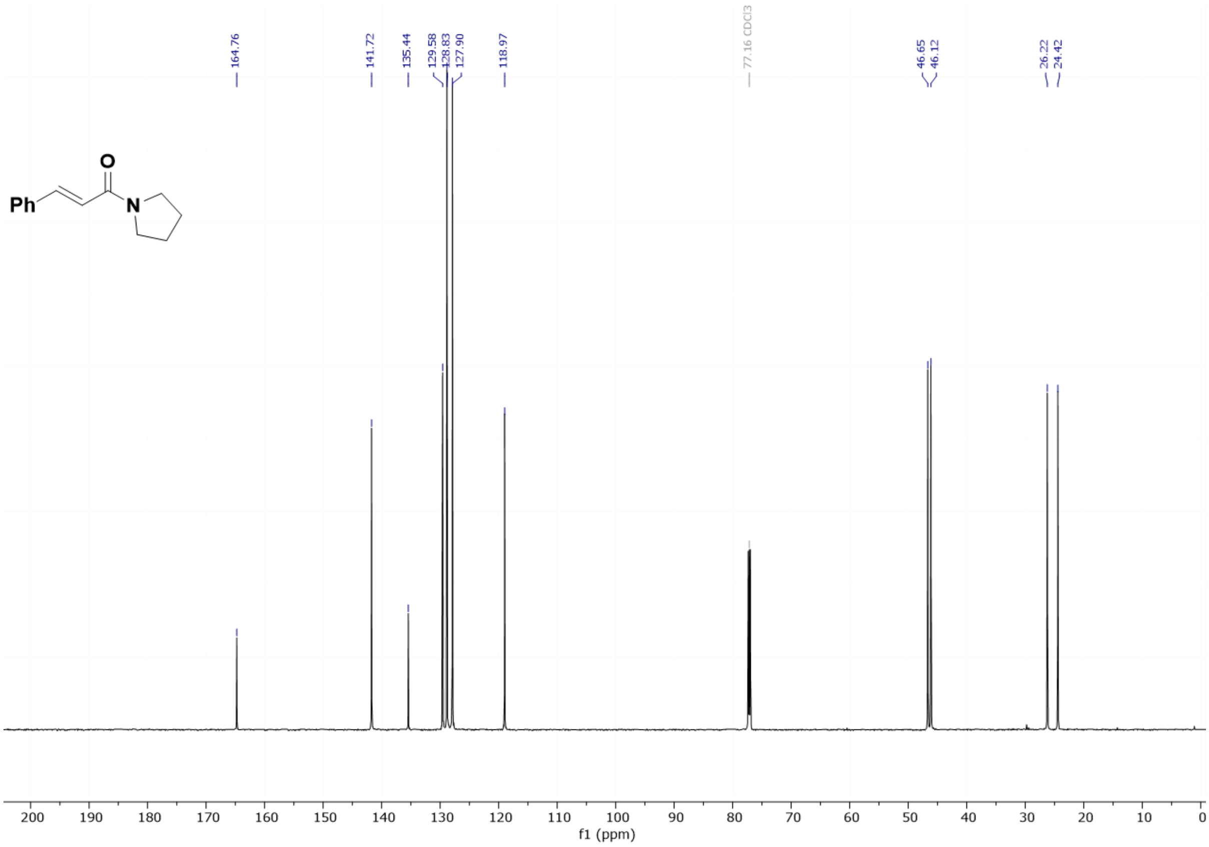

^{1}H-NMR (600 MHz, $CDCl_3$) of **11**

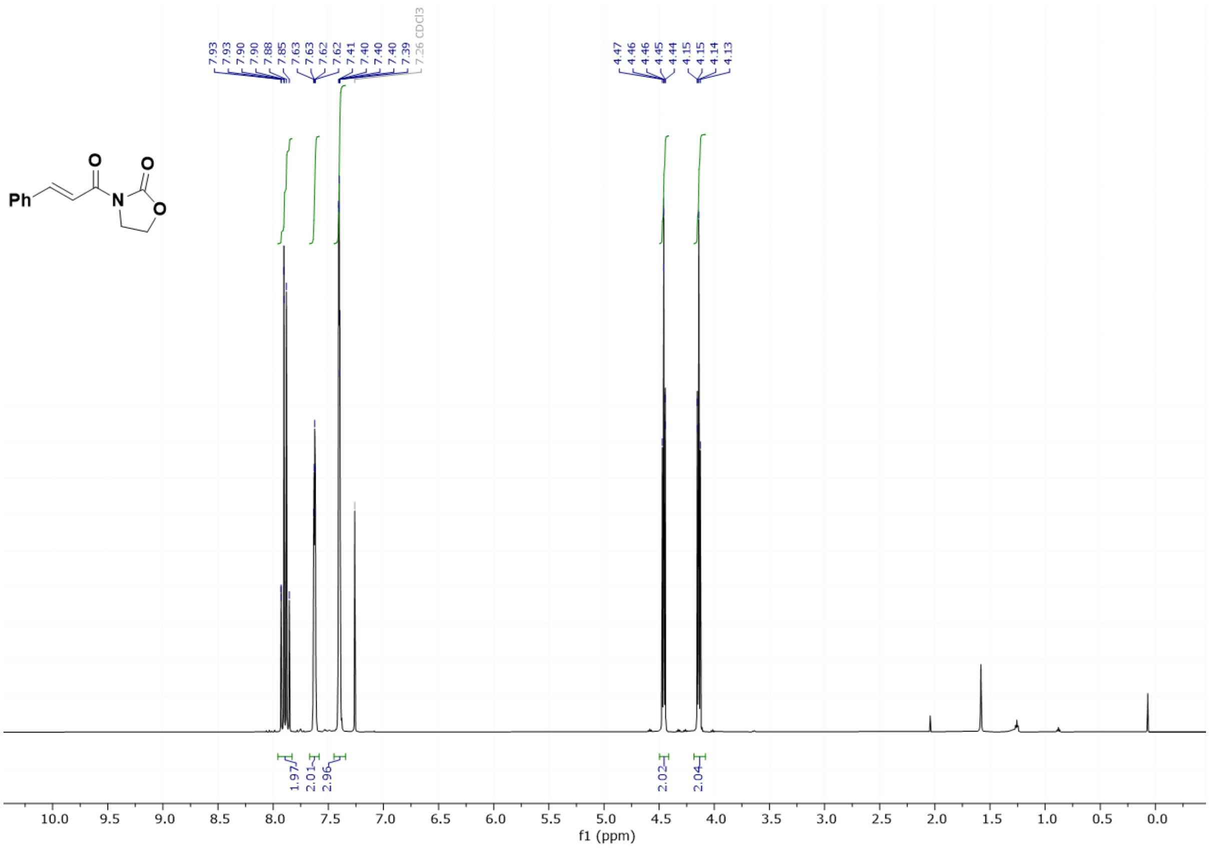

^{13}C-NMR (151 MHz, $CDCl_3$) of **11**

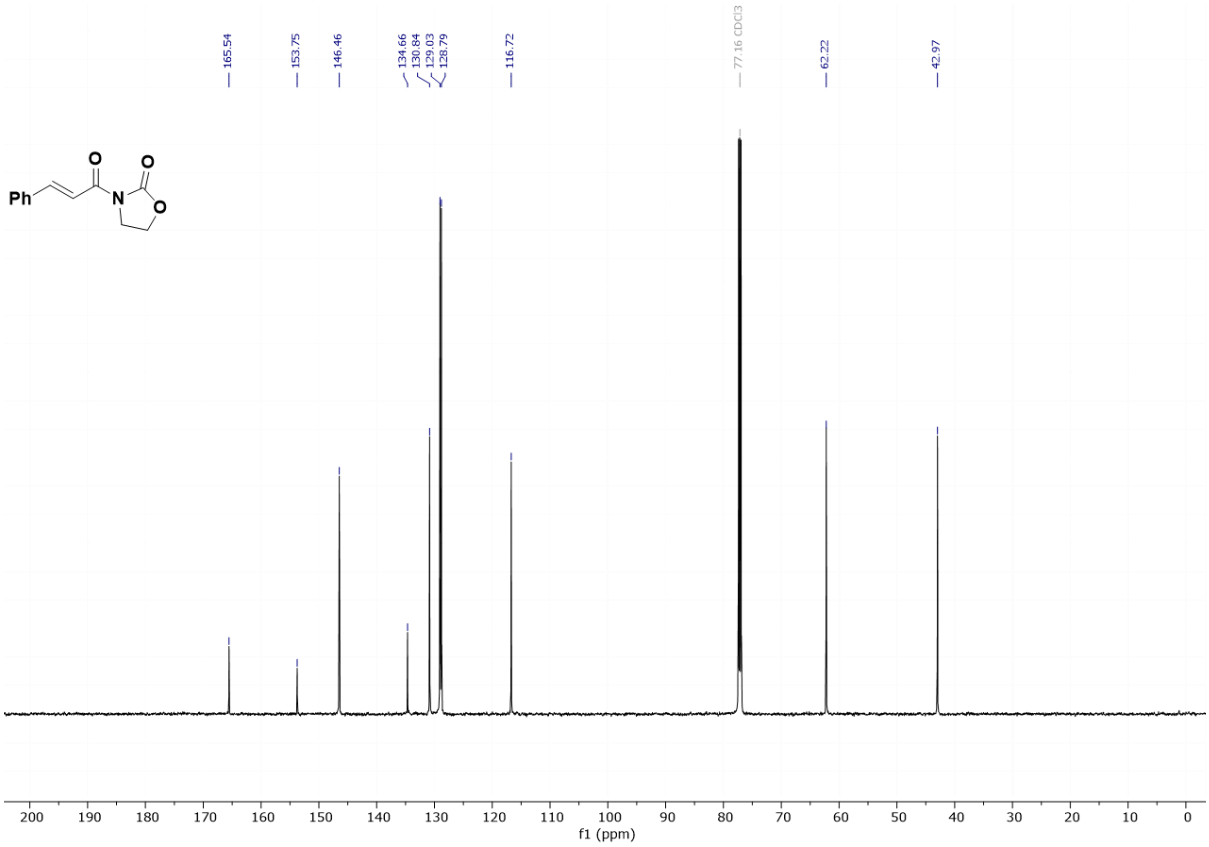

^{1}H-NMR (600 MHz, $CDCl_3$) of **3oa**

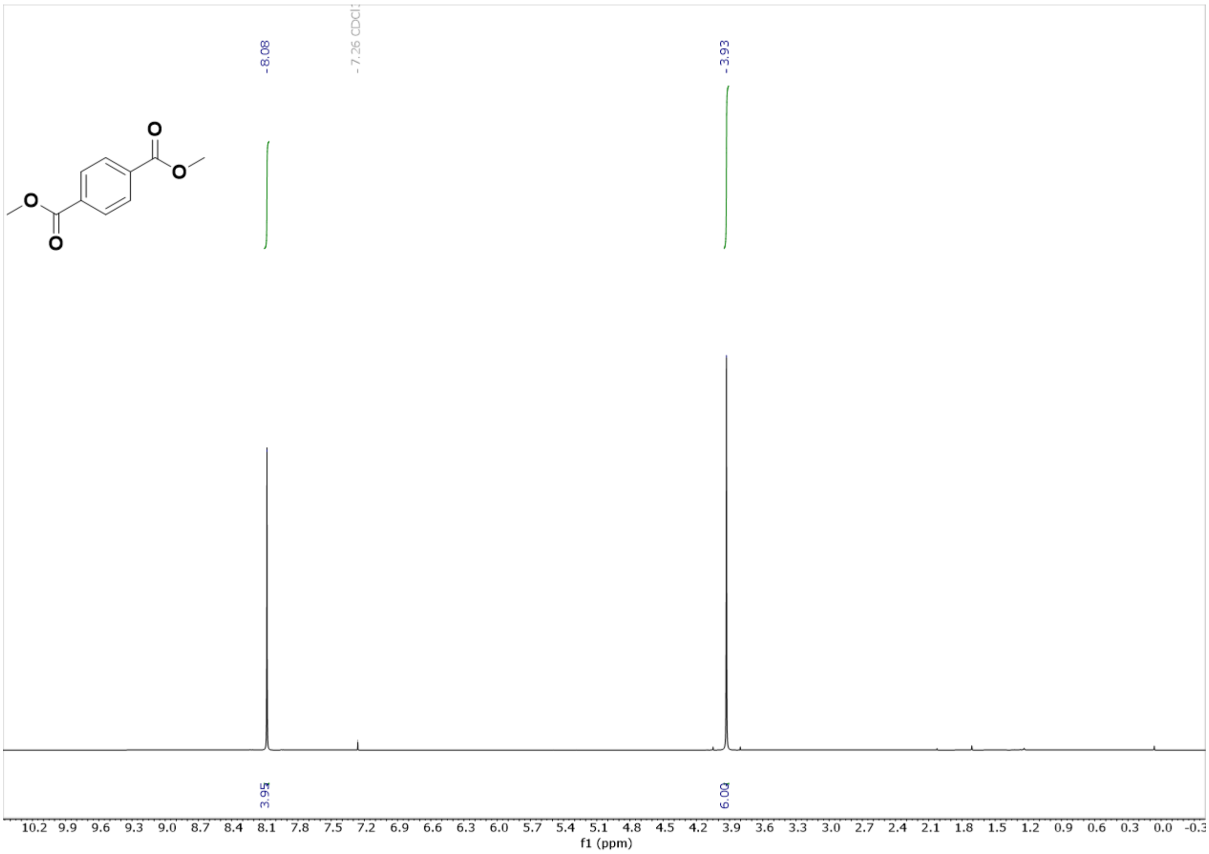

^{13}C-NMR (151 MHz, $CDCl_3$) of **3oa**

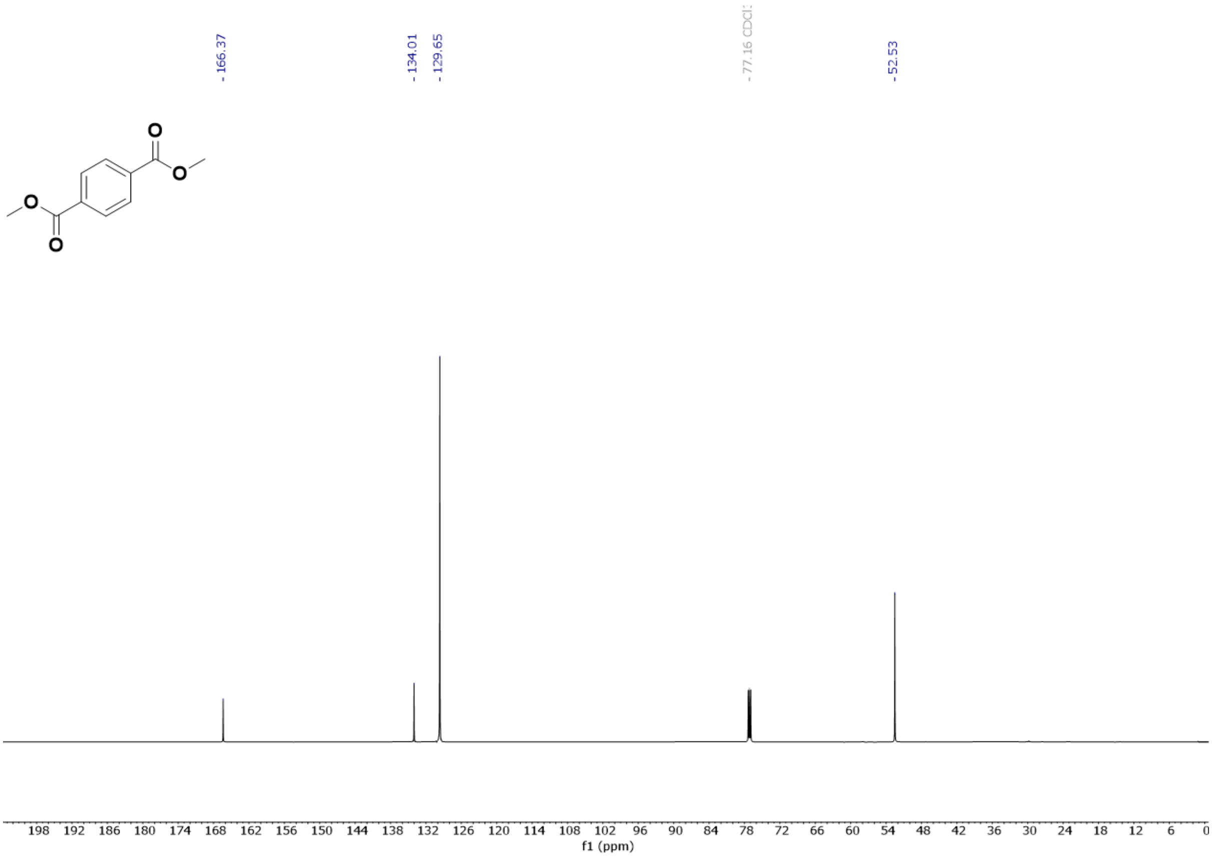

H-NMR (600 MHz, $CDCl_3$:TFA (4:1)) of **3og**

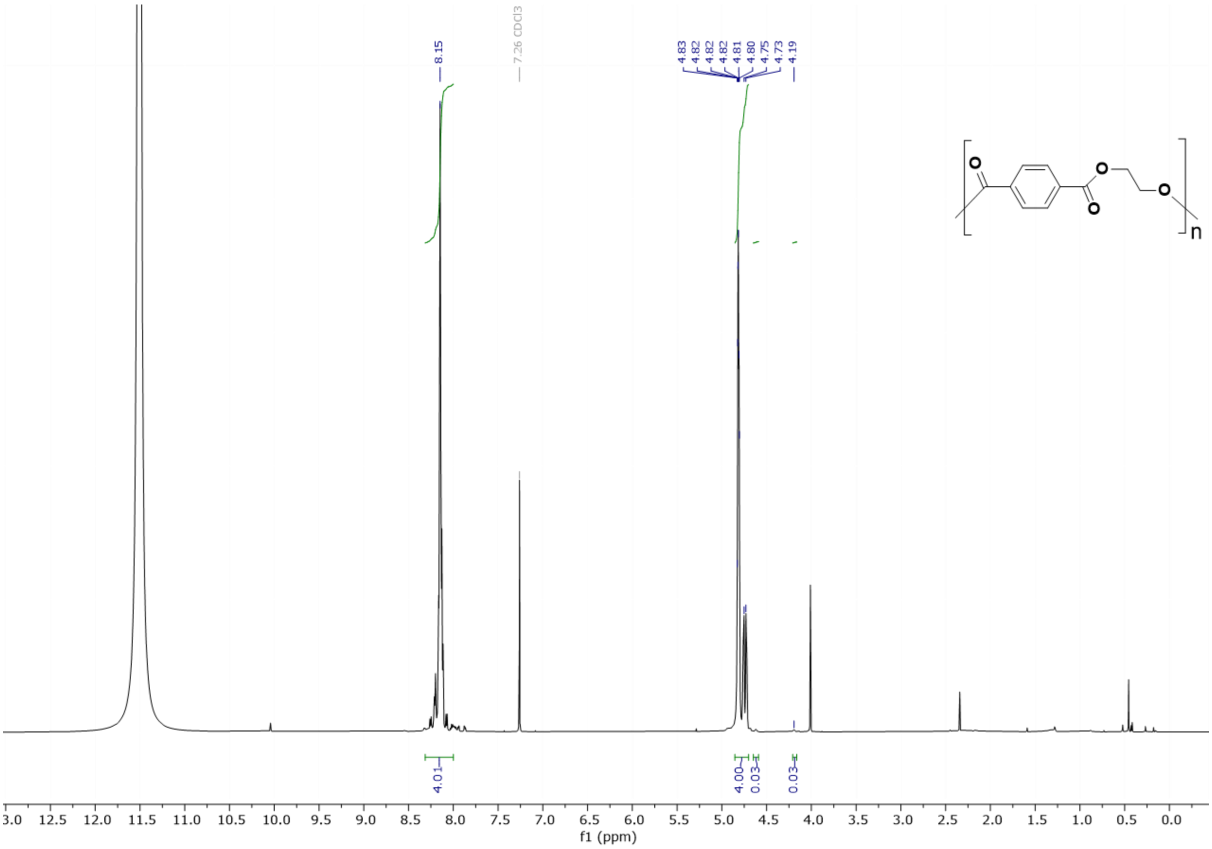

^{3}C-NMR (151 MHz, $CDCl_3$:TFA (4:1)) of **3og**

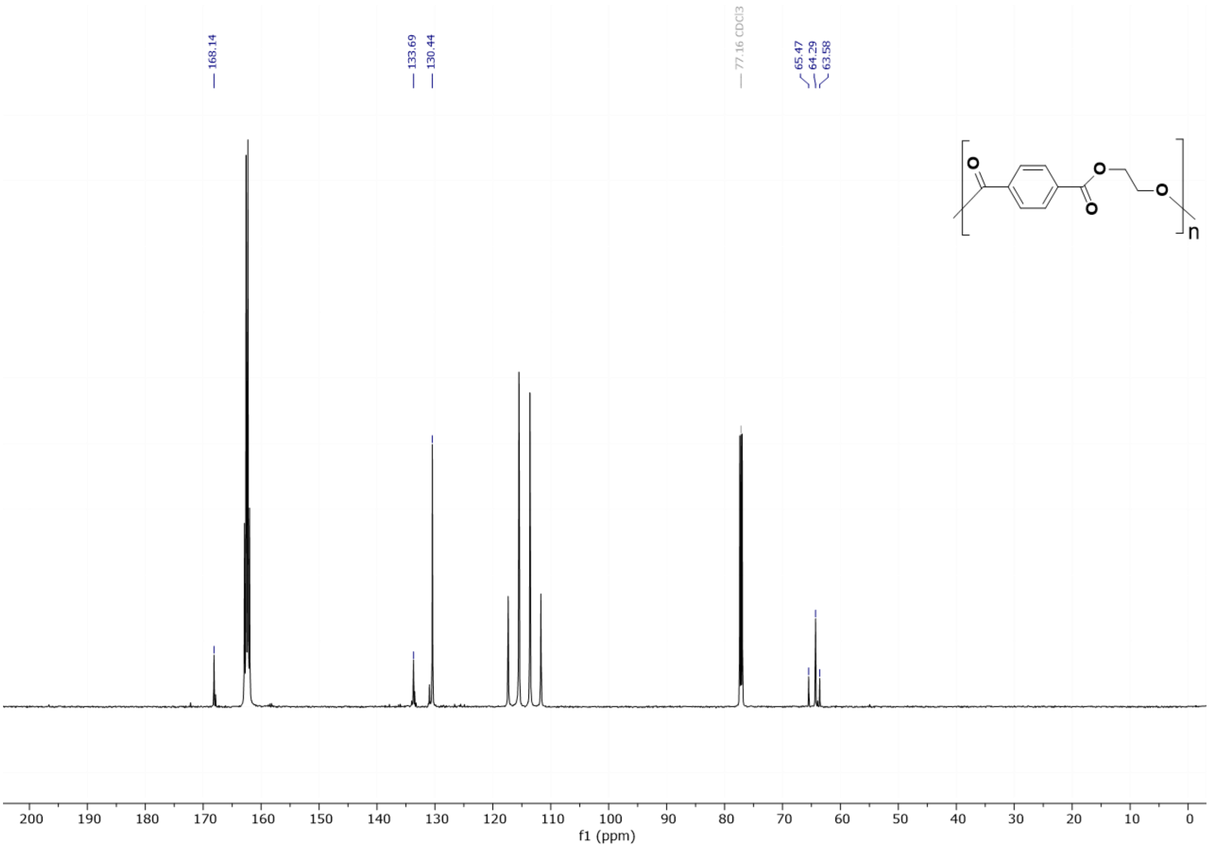

Supplement: Supplementary file 1 — ol4c00731_si_001.pdf [file ol4c00731_si_001.pdf]
